# Pseudogene *MAPK6P4*-encoded functional peptide promotes glioblastoma vasculogenic mimicry development

**DOI:** 10.1038/s42003-023-05438-1

**Published:** 2023-10-18

**Authors:** Mengyang Zhang, Yubo Zhao, Xiaobai Liu, Xuelei Ruan, Ping Wang, Libo Liu, Di Wang, Weiwei Dong, Chunqing Yang, Yixue Xue

**Affiliations:** 1 Department of Neurobiology, School of Life Sciences, China Medical University, Shenyang, 110122 PR China; 2 Key Laboratory of Cell Biology, Ministry of Public Health of China, China Medical University, Shenyang, 110122 PR China; 3 Key Laboratory of Medical Cell Biology, Ministry of Education of China, China Medical University, Shenyang, 110122 PR China; 4 Department of Neurosurgery, Shengjing Hospital of China Medical University, Shenyang, 110004 PR China; 5 Liaoning Research Center for Translational Medicine in Nervous System Disease, Shenyang, 110004 PR China; 6 Key Laboratory of Neuro-oncology in Liaoning Province, Shenyang, 110004 PR China

**Keywords:** CNS cancer, Phosphorylation

## Abstract

Glioma is the most common primary malignancy of the central nervous system. Glioblastoma (GBM) has the highest degree of malignancy among the gliomas and the strongest resistance to chemotherapy and radiotherapy. Vasculogenic mimicry (VM) provides tumor cells with a blood supply independent of endothelial cells and greatly restricts the therapeutic effect of anti-angiogenic tumor therapy for glioma patients. Vascular endothelial growth factor receptor 2 (VEGFR2) and vascular endothelial cadherin (VE-cadherin) are currently recognized molecular markers of VM in tumors. In the present study, we show that pseudogene *MAPK6P4* deficiency represses VEGFR2 and VE-cadherin protein expression levels, as well as inhibits the proliferation, migration, invasion, and VM development of GBM cells. The *MAPK6P4*-encoded functional peptide P4-135aa phosphorylates KLF15 at the S238 site, promoting KLF15 protein stability and nuclear entry to promote GBM VM formation. KLF15 was further confirmed as a transcriptional activator of LDHA, where LDHA binds and promotes VEGFR2 and VE-cadherin lactylation, thereby increasing their protein expression. Finally, we used orthotopic and subcutaneous xenografted nude mouse models of GBM to verify the inhibitory effect of the above factors on GBM VM development. In summary, this study may represent new targets for the comprehensive treatment of glioma.

## Abstract (web summary)

The pseudogene MAPK6P4 encodes a functional peptide, which phosphorylates KLF15 at the S238 site, enhancing KLF15 protein stability and nuclear entry to promote vascular mimicry in glioblastoma.

## Introduction

Glioma is one of the most common primary brain tumors in adults, accounting for more than 70% of malignant brain tumors. Among the gliomas, glioblastoma (GBM) has the highest degree of malignancy (WHO grade IV) and the strongest resistance to chemotherapy and radiotherapy^1^. Anti-angiogenesis therapy is currently a widely used treatment strategy for glioma; however, due to the presence of vasculogenic mimicry (VM), the prognosis of glioma patients remains very poor^2^. VM supplies blood to tumor cells that are independent of endothelial cells, which is distinct from classical tumor angiogenesis^3^. VM is widely present in the tissue of various malignant tumors, including hepatocellular carcinoma, osteosarcoma, lung cancer, and glioma^4–8^. In gliomas, VM promotes the growth, invasion, and metastasis of tumor cells^9^. Vascular endothelial cadherin (VE-cadherin) is an adhesion protein that is highly expressed in malignant tumor tissue and promotes the development of tumor VM^10^. Vascular endothelial growth factor receptor 2 (VEGFR2) is a member of the VEGF receptor family closely related to VM in tumors such as gastric cancer and breast cancer^11,12^. VE-cadherin and VEGFR2 are highly expressed in gliomas, where they promote VM formation and are currently recognized molecular markers of VM in tumors^8,13^.

Pseudogenes have been found in a variety of tumors, and play important roles in transcription and post-transcriptional regulation^14^. A human proteomics study reported 107 peptides encoded by pseudogenes, suggesting that these peptides are an important direction in the study of pseudogene functions^15,16^. In recent years, pseudogenes encoding polypeptides via open reading frames (ORFs) have attracted increasing attention. For example, the pseudogene OCT4-PG1 promotes the malignant progression of chronic myeloid leukemia by encoding polypeptides^17^. Similarly, the pseudogene NANOGP8 drives the occurrence and development of prostate cancer by encoding polypeptides^18^. However, the functions of pseudogene-encoded polypeptides in GBM remain largely unknown.

In the present study, we showed that *MAPK6P4* encodes a functional polypeptide P4-135aa, and explored its role in GBM VM development. We identified physiological substrates of P4-135aa and determined that they are regulated by phosphorylation. We further explored the downstream effects of this regulation and its effects on VEGFR2 and VE-cadherin lactylation. We analyzed the mode of interaction between these molecules, as well as their role and mechanisms during VM development in GBM cells. This research will enable the identification of new mechanisms of VM development in gliomas and new targets for the treatment of GBM.

## Results

### *MAPK6P4* deletion suppresses VM in GBM cells

**Fig. 1:**
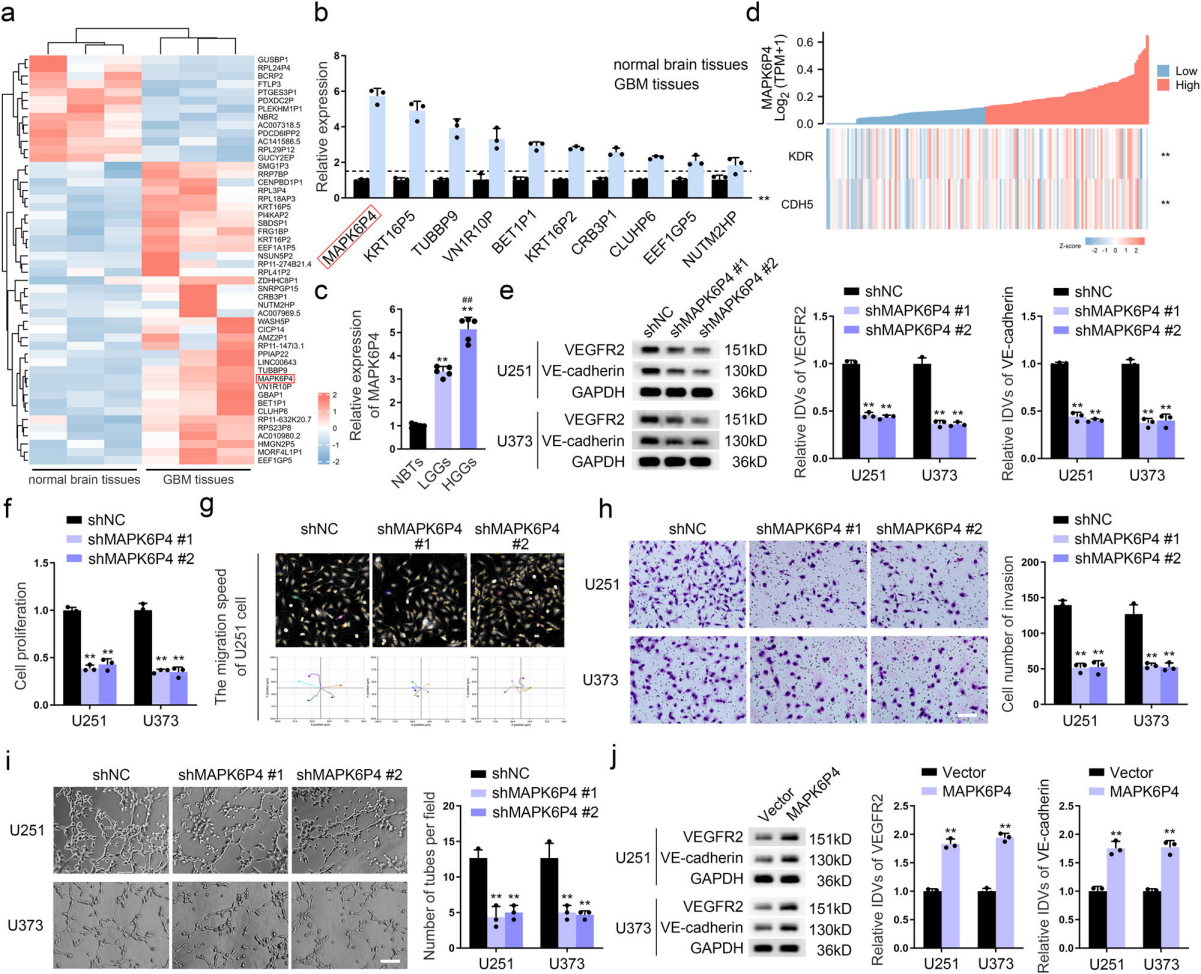
*MAPK6P4* deletion suppresses VM in GBM cells. **a** RNA array analysis screens the differentially expressed pseudogenes in normal brain tissues (*n* = 3) and GBM tissues (*n* = 3). **b** qRT-PCR was used to detect the expression of *MAPK6P4* in normal human astrocytes (NHA) and six strains of malignant glioma cells (A172, U87, U118, U251, U373 and LN229). Values are expressed as mean ± standard deviation (SD) (*n* = 3), **P < 0.01, compared with NHA group. **c** qRT-PCR was used to detect the expression of *MAPK6P4* in normal brain tissues (NBTs), low-grade gliomas (LGGs: Grade I–II), and high-grade gliomas (HGGs: Grade III–IV). Each value represents the mean ± SD (*n* = 5, each group), **P < 0.01, compared with NBTs group; ^##^*P* < 0.01, compared with LGGs group. **d** Co-expression correlation heatmap of *MAPK6P4*, *KDR* and *CDH5*. **e** The VEGFR2 and VE-cadherin protein levels in U251 and U373 cells with *MAPK6P4* deficiency were determined by western blotting. **f** CCK8 assay was used to detect proliferation capacity of U251 and U373 cells with *MAPK6P4* knockdown. **g** The Hstudio M4 system observed the capacity for migration in U251 cells with *MAPK6P4* knockdown. **h** Transwell method was used to detect the capacity for invasion in U251 and U373 cells with *MAPK6P4* knockdown (×200; scale bar represents 100 μm). **i** Three-dimensional cell culture method was used to detect the change of VM in U251 and U373 cells with *MAPK6P4* knockdown (×200; scale bar represents 100 μm). **P < 0.01, compared with shNC (Negative control) group. **j** The VEGFR2 and VE-cadherin protein levels in U251 and U373 cells with *MAPK6P4* overexpression were determined by western blotting. **P < 0.01, compared with the Vector group. The data are the means ± SD of three independent experiments.

To identify GBM-specific pseudogenes, we screened differentially expressed pseudogenes from three normal brain and three GBM tissue samples using RNA microarray analysis (Fig. 1a). We then verified these by Real-Time PCR (qRT-PCR) and confirmed that the difference in expression between normal and GBM tissue was the most pronounced for the pseudogene *MAPK6P4* (Fig. 1b). *MAPK6P4* expression was significantly higher in glioma tissues than in normal brain tissues, especially in high-grade glioma tissues (Fig. 1c). Figure 1d showed coexpression correlation heatmap between *MAPK6P4* and VM formation-related proteins using RNA-seq data accessed via the TCGA (https://portal.gdc.cancer.gov/) database. We performed qRT-PCR and western blotting in six GBM cell lines and found that *MAPK6P4* expression was significantly upregulated in GBM cell lines, especially in U251 and U373 cells (Fig. S1.1 a); however, no significant changes were detected in mRNA and protein expression of the parental gene MAPK6 (Fig. S1.1 b, c). There was also no significant association between MAPK6 and poor prognosis in GBM patients (Fig. S1.1 d). Next, we directly knocked down *MAPK6P4* using two specific shRNAs (Fig. S1.1 e) and found that knockdown of *MAPK6P4* significantly reduced the protein levels of VEGFR2 and VE-cadherin (Fig. 1e). We further found using qRT-PCR and western blot assays that MAPK6P4 knockdown had no significant effect on the mRNA and protein expression of the parental gene MAPK6 (Fig. S1.1f and g). Knockdown of *MAPK6P4* significantly inhibited the proliferation, migration, invasion, and VM capacity of GBM cells (Fig. 1f–i, Fig. S1.1h). We established a GBM cell line stably overexpressing *MAPK6P4* (Fig. S1.1 i). The upregulation of *MAPK6P4* significantly increased the expression of VEGFR2 and VE-cadherin (Fig. 1j), as well as the proliferation, migration, invasion, and VM capacity of GBM cells (Fig. S1.1 j–m). To better model the biology of the tumors, we have additionally performed reproducible experiments using primary patient-derived glioblastoma (PPDG) cells. Results showed that *MAPK6P4* was highly expressed in PPDG cells (Fig. S1.2a). We knocked down *MAPK6P4* (transfection efficiency shown in Fig. S1.2 b) and observed that VEGFR2 and VE-cadherin expression were significantly downregulated as a result (Fig. S1.2c). The proliferation, migration, invasion, and VM formation ability was significantly reduced in *MAPK6P4*-deficient cells (Fig. S1.2d–g). Overexpression of *MAPK6P4* significantly enhanced these abilities (Fig. S1.2h–l).

### *MAPK6P4* promotes VM development in GBM cells via the encoded peptide P4-135aa

**Fig. 2:**
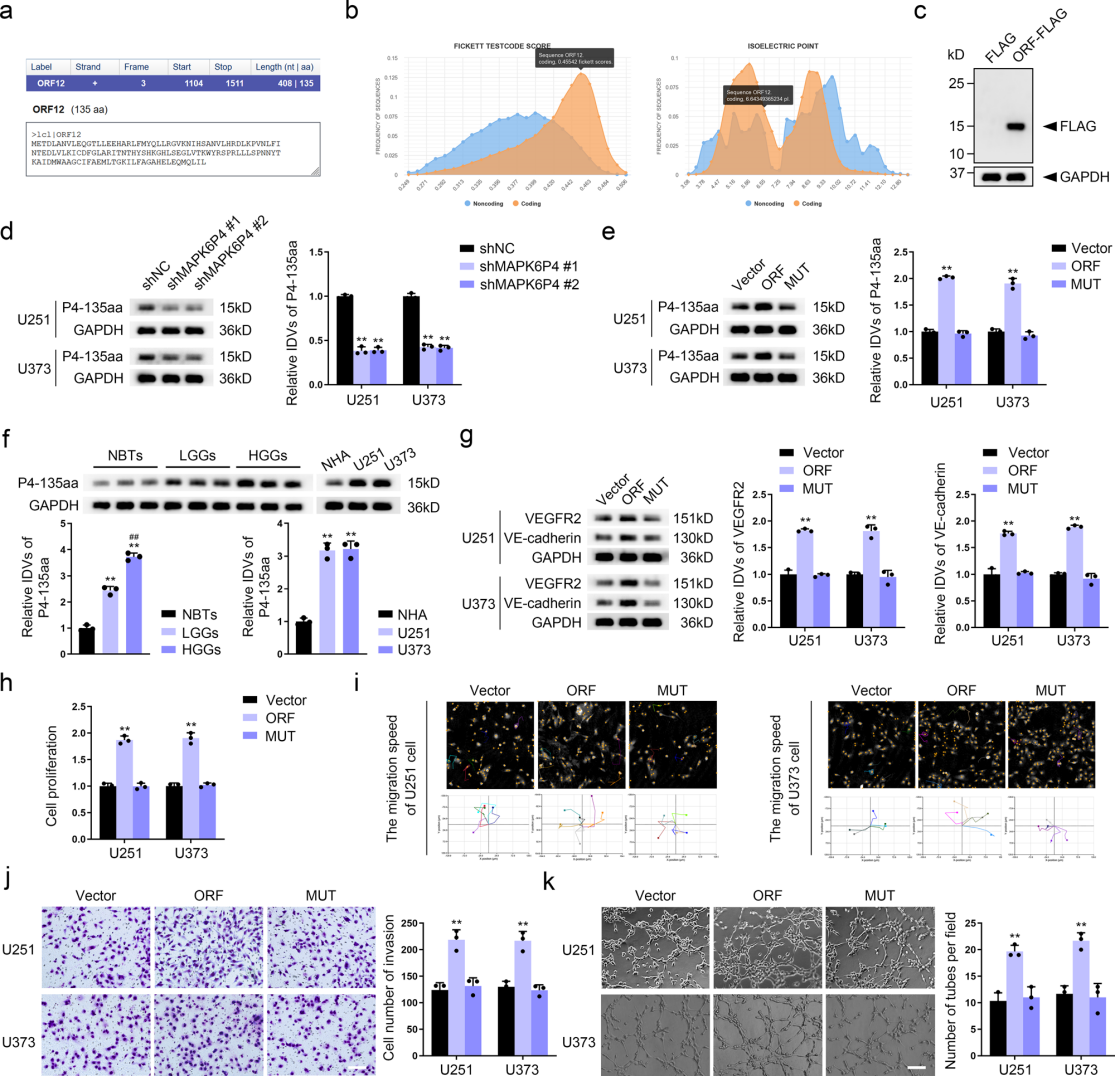
*MAPK6P4* promotes VM development in GBM cells via the encoded peptide P4-135aa. **a** ORFfinder analysis found that ORF12 of *MAPK6P4* can encode P4-135aa. **b** CPC2 analysis results indicate ORF12 with a putative polypeptide. **c** The putative *MAPK6P4* coding region of 135 amino acids was cloned into a pcDNA3.1/FLAG vector and was expressed as a fusion protein with a FLAG tag in HEK-293T cells. The expected 15 kDa fusion protein was detected by western blot using FLAG antibody. **d** Western blot was used to detect the expression of P4-135aa in U251 and U373 cells with *MAPK6P4* knockdown. **e** Western blot was used to detect the expression of P4-135aa in U251 and U373 cells transfected with the indicated constructs. Vector, empty vector; ORF, *MAPK6P4* ORF12; MUT, start codon ATG of *MAPK6P4* ORF12 was mutated to ATT. **f** Western blot was used to detect the expression of P4-135aa in NBTs, LGGs, and HGGs (left), **P < 0.01 compared with NBTs group; ^##^*P* < 0.01 compared with LGGs group (*n* = 3). Western blot was used to detect the expression of P4-135aa in NHA, U251 and U373 cells (right), **P < 0.01 compared with NHA group (*n* = 3). **g** The VEGFR2 and VE-cadherin protein levels in U251 and U373 cells transfected with the indicated constructs were determined by western blotting. **h** CCK8 assay was used to detect proliferation capacity in U251 and U373 cells transfected with the indicated constructs. **i** The Hstudio M4 system observed the capacity for migration in U251 and U373 cells transfected with the indicated constructs. **j** Transwell method was used to detect the capacity for invasion in U251 and U373 cells transfected with the indicated constructs (×200; scale bar represents 100 μm). **k** Three-dimensional cell culture method was used to detect the change of VM in U251 and U373 cells transfected with the indicated constructs (×200; scale bar represents 100 μm). **P < 0.01, compared with the corresponding shNC/Vector group. The data are the means ± SD of three independent experiments.

ORFfinder (https://www.ncbi.nlm.nih.gov/orffinder/) analysis revealed that *MAPK6P4* encodes a polypeptide of 135 aa at ORF12 (Fig. 2a). CPC2 database (http://cpc2.gao-lab.org/) analysis revealed the potential of ORF12 to encode polypeptides (Fig. 2b). To confirm that the *MAPK6P4* transcript can be translated into a peptide, we cloned the coding sequence of *MAPK6P4* into the pcDNA3.1/FLAG expression vector using the FLAG tag antibody. We detected a peptide of ~15 kDa in HEK-293T cells stably transfected with this vector, while this polypeptide was not detected in HEK-293T cells transfected with the empty vector (Fig. 2c). Figure S2.1 a shows the nucleotide sequence of *MAPK6P4*-ORF12 and the amino acid sequence of the human *MAPK6P4* ORF12-encoded polypeptide, which we named P4-135aa and used to customize a specific antibody. To further verify whether the ATG codon of *MAPK6P4*-ORF12 was functional, pcDNA-ORF and pcDNA-ORF-GFP were constructed and transfected into GBM cells, and the expression of the GFP fusion protein was quantified after 24 h in GBM cells (Fig. S2.1 b). The GFP fusion protein was detected using both anti-GFP and P4-135aa antibodies, and no GFP fusion protein was detected when the ORF ATG codon was mutated (ORFmut-GFPmut), indicating that the *MAPK6P4* ORF12 ATG codon was functional (Fig. S2.1 c). We next knocked down *MAPK6P4* in GBM cells and found that the expression of P4-135aa was significantly decreased, while the expression level of P4-135aa was significantly increased in GBM cells overexpressing *MAPK6P4* (Fig. 2d, Fig. S2.1 d). The expression level of P4-135aa increased significantly in GBM cells transfected with *MAPK6P4* ORF12, while no significant change in P4-135aa levels was observed in GBM cells transfected with an ORF with a nonfunctional ATG codon mutation (named MUT) (Fig. 2e). Therefore, we investigated the expression and function of P4-135aa in GBM and found that this peptide was highly expressed in glioma tissue and cells, and is also positively correlated with glioma grade (Fig. 2f, Fig. S2.1 e). The expression of VEGFR2 and VE-cadherin, as well as the proliferation, migration, invasion, and VM formation ability of GBM cells were also significantly increased in GBM cells transfected with the ORF (Fig. 2g–k). Experimental results in PPDG cells showed that the expression level of P4-135aa was significantly reduced after silencing of *MAPK6P4* (Fig. S2.2 a), and the expression level of P4-135aa increased significantly in PPDG cells transfected with *MAPK6P4* ORF12 (Fig. S2.2 b). P4-135aa was highly expressed in PPDG cells (Fig. S2.2 c). The expression of VEGFR2 and VE-cadherin, as well as the proliferation, migration, invasion, and VM formation ability of GBM cells were also significantly increased in PPDG cells transfected with the ORF (Fig. S2.2 d-h).

### P4-135aa phosphorylates KLF15 at S238, promoting VM formation in GBM cells

**Fig. 3:**
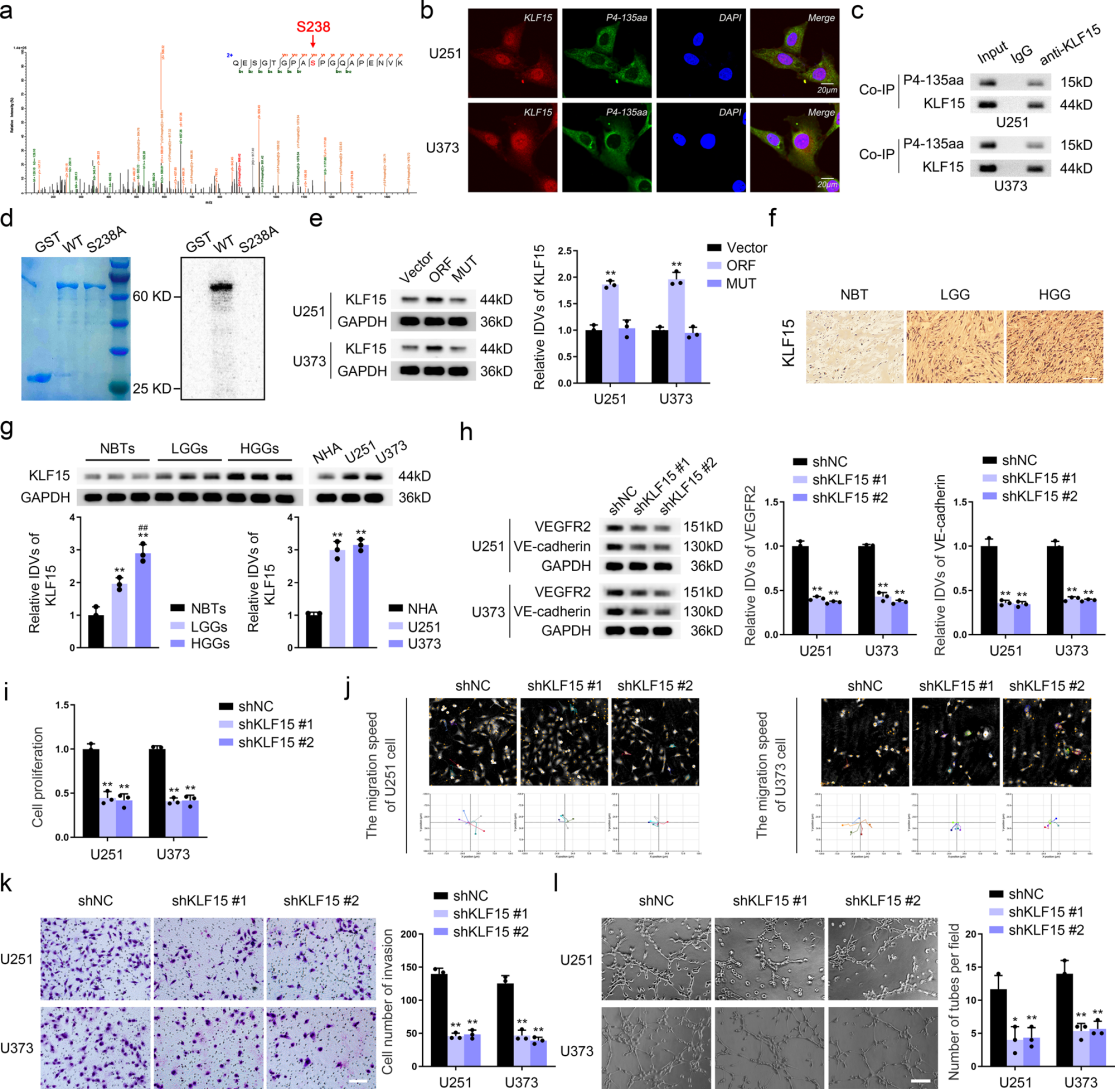
P4-135aa phosphorylates KLF15 at S238, promoting VM formation in GBM cells. **a** Analysis of phosphorylation of KLF15 by mass spectrometry (phosphorylation site at S238). **b** Co-localization of P4-135aa and KLF15 in U251 and U373 cells was determined by IF staining and observed by laser scanning confocal microscopy (Scale bar represents 20 µm). **c** The interaction of P4-135aa and KLF15 in the cytoplasmic proteins of U251 and U373 cells was determined by co-IP assays. **d** Phosphorylation of KLF15 by P4-135aa was detected by in vitro kinase assays. The left image shows the fusion protein added in in vitro kinase system. The right image shows the phosphorylatable state of KLF15-WT and KLF15 with the sited mutations on S238A. **e** Western blot was used to detect the expression of KLF15 in U251 and U373 cells transfected with the indicated constructs. **f** IHC shows the expression and distribution of KLF15 in NBT, LGG and HGG (×200; scale bar represents 150 μm). **g** Western blot was used to detect the expression of KLF15 in NBTs, LGGs, and HGGs (left), **P < 0.01 compared with NBTs group; ^##^*P* < 0.01 compared with LGGs group (*n* = 3). Western blot was used to detect the expression of KLF15 in NHA, U251 and U373 cells (right), **P < 0.01 compared with NHA group (*n* = 3). **h** The VEGFR2 and VE-cadherin protein levels in U251 and U373 cells with KLF15 deficiency were determined by western blotting. **i** CCK8 assay was used to detect the proliferation capacity of U251 and U373 cells with KLF15 knockdown. **j** The Hstudio M4 system observed the capacity for migration in U251 cells with KLF15 knockdown. **k** Transwell method was used to detect the capacity for invasion in U251 and U373 cells with KLF15 knockdown (×200; scale bar represents 100 μm). **l** Three-dimensional cell culture method was used to detect the change of VM in U251 and U373 cells with KLF15 knockdown (×200; scale bar represents 100 μm). **P < 0.01, compared with the corresponding shNC/Vector group. The data are the means ± SD of three independent experiments.

The SMART database (http://smart.embl-heidelberg.de/) revealed the presence of a phosphorylated serine/threonine kinase catalytic (S_TKc) domain in the P4-135aa (Fig. S3.1 a). We next performed immunoprecipitation (IP) assays with the P4-135aa antibody to identify the IP complex by mass spectrometry and found that KLF15 was present and was phosphorylated at serine 238 (S238) (Fig. 3a). KLF15 was predicted to be highly expressed in GBM using TCGA database (Fig. S3.1 b). To further validate the mechanism of KLF15 regulation by P4-135aa, we first observed the localization of P4-135aa and KLF15 in U251 and U373 cells by immunofluorescence (IF) assays. P4-135aa was mainly distributed in the cytoplasm, while KLF15 showed nuclear-cytoplasmic co-distribution and was mainly distributed in the nucleus (Fig. 3b). Since P4-135aa could colocalize with KLF15 in the cytoplasm, we verified the binding of P4-135aa and KLF15 using a co-immunoprecipitation (co-IP) assay (Fig. 3c). Cross-species analysis revealed that the residue S238 was conserved (Fig. S3.1 c). We next used a site-directed mutagenesis kit to mutate S238 into alanine (S238A, alanine, A) to mimic the state of amino acid dephosphorylation. An in vitro kinase assay revealed that wild-type KLF15 was phosphorylated when P4-135aa was present in the system, while S238A-KLF15 was not phosphorylated (Fig. 3d). Next, we overexpressed the P4-135aa ORF and found that the protein level of KLF15 increased significantly (Fig. 3e), but not the mRNA level (Fig. S3.1 d). Based on this, we speculated that KLF15 is regulated by post-translational modifications via P4-135aa.

We next investigated the expression and function of KLF15 in GBM. KLF15 was highly expressed in GBM cells (Fig. 3f, g). We knocked down KLF15 using two specific shRNAs (Fig. S3.1 e) and found that knockdown of KLF15 significantly reduced the protein levels of VEGFR2 and VE-cadherin (Fig. 3h), and inhibited the proliferation, migration, invasion, and VM capacity of GBM cells (Fig. 3i–l). We established stable KLF15-overexpressing GBM cell lines (Fig. S3.1f) and observed an increase in the protein levels of VEGFR2 and VE-cadherin in the KLF15-overexpressing cells, as well as in their proliferation, migration, invasion, and VM capacity (Fig. S3.1 g-l). The results of the experiments performed in PPDG cells are consistent with the above-described results (Fig. S3.2 a–l).

### Phosphorylation of KLF15 at S238 increases the stability of KLF15 and promotes GBM VM development in vivo

**Fig. 4:**
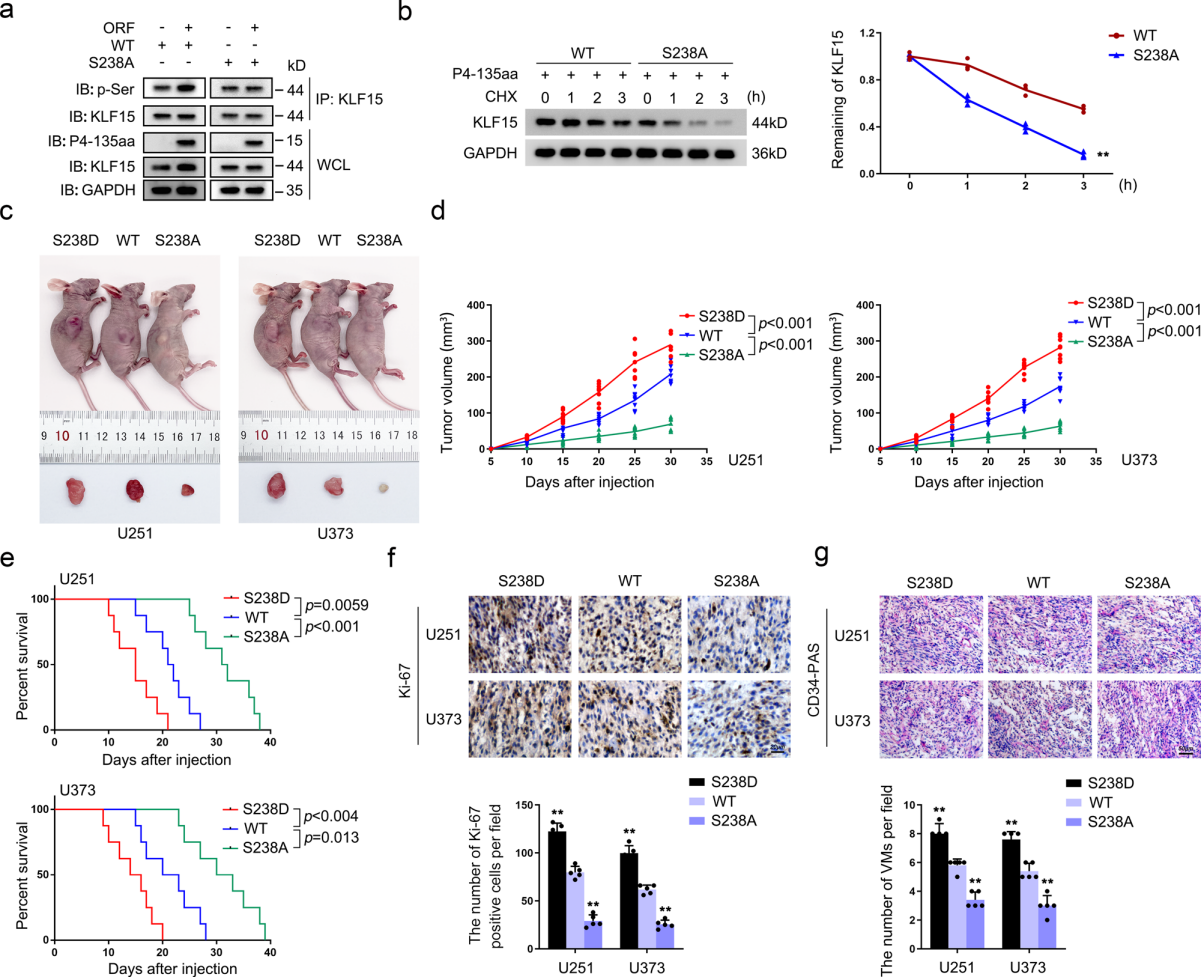
Phosphorylation of KLF15 at S238 increases the stability of KLF15 and promotes GBM VM development in vivo. **a** IP and IB analysis of KLF15 phosphorylation in HEK-293T cells transfected with the indicated constructs. **b** Half-life of KLF15 protein was measured in HEK-293T cells transfected with the indicated constructs after treatment with CHX. **P < 0.01, compared with corresponding the WT group (*n* = 3). **c** The nude mice carrying U251 and U373 cells suspension formed tumors in respective groups are shown. The sample tumors resected from respective groups are shown. **d** Tumor growth curves are shown. Tumor volume was calculated every 5 days after U251 and U373 cells suspension injection, and the tumor was resected at 30 days. Data are presented as the mean ± SD (*n* = 8, each group). **e** Survival curves of nude mice U251 and U373 cells suspension injected into the right striatum are shown (*n* = 8, each group, log-rank test). **f** Ki-67 immunohistochemical staining in orthotopic xenografts (×400; scale bar represents 20 μm). **g** CD34-PAS double staining was applied to detect the VM in orthotopic xenografts (×200; scale bar represents 50 μm). Data are presented as the mean ± SD (*n* = 5, each group). **P < 0.01, compared with WT group.

We investigated the possible biological role of P4-135aa in GBM. First we using phosphorylation (p-Ser) and KLF15 antibody for western blot, result showed that phosphorylation level and protein expression.level of wild-type KLF15 but not KLF15 mutant S238A was increased with overexpression of P4-135aa (Fig. 4a). By treating cells with cycloheximide, we observed that the KLF15 protein half-life was substantially prolonged in P4-135aa-overexpressing cells (Fig. S4.1 a). The S238A mutation significantly shortened the KLF15 protein half-life compared with levels observed in the S238 wild type (Fig. 4b). Furthermore, we found that treatment with the proteasome inhibitor MG132 reversed the reduction in KLF15 protein levels caused by the P4-135aa deletion in U251 and U373 cells (Fig. S4.1b). We then overexpressed P4-135aa in HEK-293T cells, co-transfected them with KLF15 wildtype or KLF15 S238A, and found that KLF15 ubiquitination levels were significantly increased in cells transfected with the mutated phosphorylation site (Fig. S4.1 c), proving that non-phosphorylated KLF15 is more prone to ubiquitin-proteasomal degradation. These results suggest that phosphorylation of KLF15 S238 significantly increases the stability of KLF15. Further detection by western blotting and IF assays showed that KLF15 phosphorylation site mutation decreased the amount of KLF15 in the nucleus and increased it in the cytoplasm (Fig S4.1 d,e). We performed further experiments to verify whether the depletion of KLF15 could suppress the effects of *MAPK6P4* overexpression. The results showed that the depletion of KLF15 could reverse the *MAPK6P4* overexpression-induced promotion of VEGFR2 and VE-cadherin protein expression in GBM cells and their capacities for proliferation, migration, invasion, and VM (Fig.S4.2 a–e). The role of phospho-S238 KLF15 was also examined in vivo. BALB/c nude female mice were subcutaneously injected with GBM cells transfected with KLF15 WT, KLF15 S238D (simulating the phosphorylation of serine), and KLF15 S238A. KLF15 S238D tumor xenografts grew significantly faster than KLF15 WT, and KLF15 S238A tumor xenografts grew significantly slower than KLF15 WT (Fig. 4c, d). Meanwhile, we injected GBM cells into nude mice to establish a model of orthotopic GBM xenograft tumors and observed that KLF15 S238D mice had a shorter survival time, whereas KLF15 S238A mice survived for significantly longer (Fig. 4e). Consistently, immunohistochemical (IHC) analysis in orthotopic xenografts revealed strong Ki-67 staining in KLF15 S238D, whereas S238A displayed weak Ki-67 staining (Fig. 4f). CD34-PAS staining showed that the amount of VM significantly increased in KLF15 S238D orthotopic xenografts and significantly decreased in KLF15 S238A (Fig. 4g). To avoid differences caused by cell clones, we additionally generated stable doxycycline-induced wild type (WT), phosphorylation site mutant (S238A) and phosphorylation site activation (S238D) KLF15 in mice to explore the role of KLF15 phosphorylation sites mutation in PPDG cells. Subcutaneous and orthotopic xenograft tumor nude mice were constructed separately, cells were treated with doxycycline for 3 days before injection. Doxycycline was administered in the animal’s food immediately after surgical implantation of tumor cells. Results showed that KLF15 S238D tumor xenografts grew significantly faster than KLF15 WT, and KLF15 S238A tumor xenografts grew significantly slower than KLF15 WT (Fig. S4.2f, g), and KLF15 S238D mice had a shorter survival time, whereas KLF15 S238A mice survived for significantly longer (Fig. S4.2h).

### LDHA is a target of KLF15 and can promote VM

**Fig. 5:**
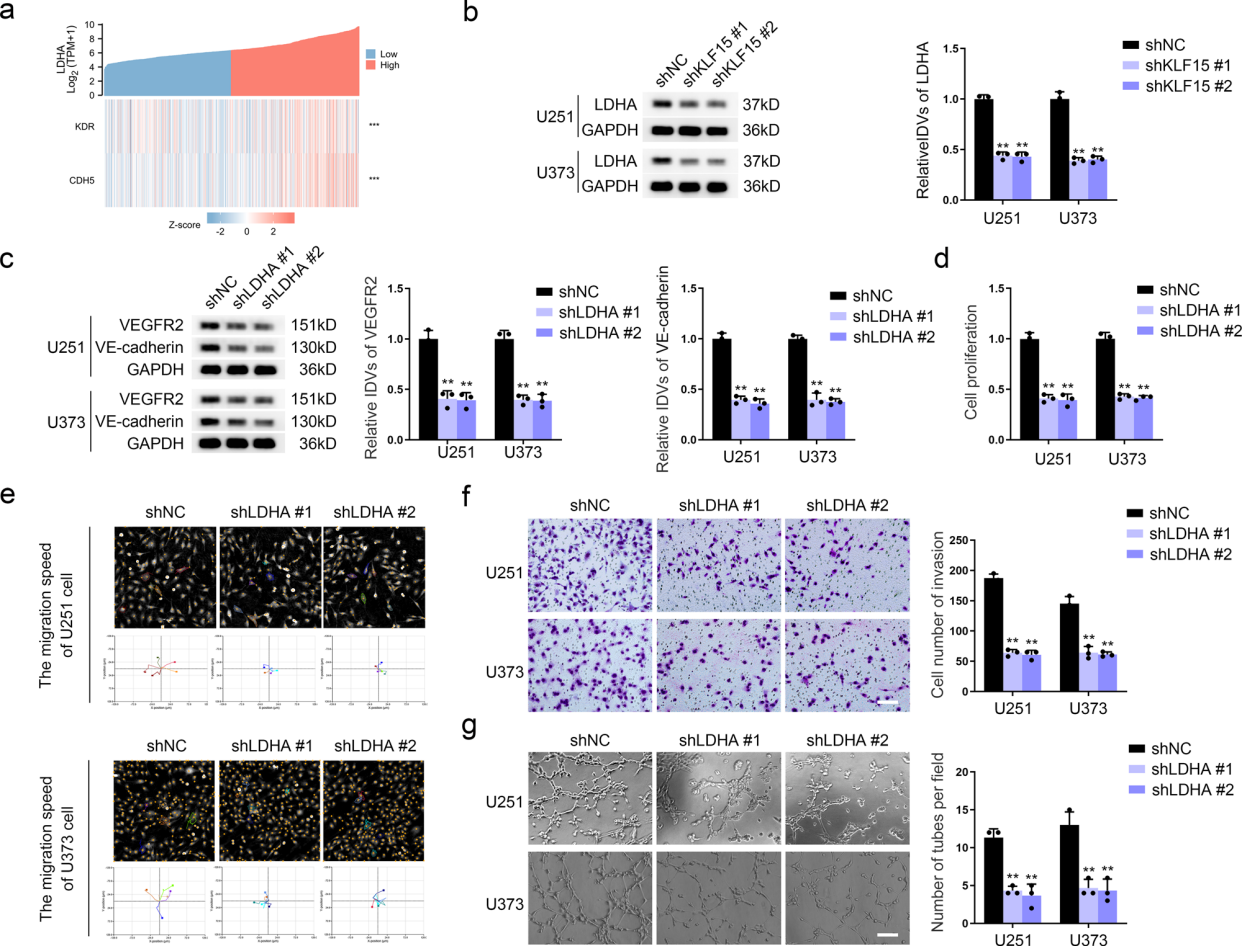
LDHA is a target of KLF15 and can promote VM. **a** Co-expression correlation heatmap of *LDHA*, *KDR* and *CDH5*. **b** The LDHA protein levels in U251 and U373 cells with KLF15 deficiency were determined by western blotting. **c** The VEGFR2 and VE-cadherin protein levels in U251 and U373 cells with LDHA deficiency were determined by western blotting. **d** CCK8 assay was used to detect the proliferation capacity of U251 and U373 cells with LDHA knockdown. **e** The Hstudio M4 system observed the capacity for migration in U251 cells with LDHA knockdown. **f** Transwell method was used to detect the capacity for invasion in U251 and U373 cells with LDHA knockdown (×200; scale bar represents 100 μm). **g** Three-dimensional cell culture method was used to detect the change of VM in U251 and U373 cells with LDHA knockdown (×200; scale bar represents 100 μm). **P < 0.01, compared with the shNC group. The data are the means ± SD of three independent experiments.

We subjected KLF15-deficient U251 and U373 cells to an mRNA PCR array and validated those results by qRT-PCR. LDHA mRNA expression was significantly reduced after KLF15 knockdown (Fig. S5.1 a–c). Figure 5a showed coexpression correlation heatmap between LDHA and VEGFR2 and VE-cadherin using RNA-seq data accessed via the TCGA database. The protein expression level of LDHA was significantly reduced after the KLF15 knockdown (Fig. 5b). LDHA was highly expressed in glioma tissue and cells (Fig. S5.1 d, e). We directly knocked down LDHA using two specific shRNAs (Fig. S5.1f) and observed that VEGFR2 and VE-cadherin expression were significantly downregulated as a result (Fig. 5c). The proliferation, migration, invasion, and VM formation ability of GBM cells was significantly reduced in LDHA-deficient cells (Fig. 5d–g). Overexpression of LDHA significantly enhanced these abilities (Fig. S5.1 g–l). The results of the experiments performed in PPDG cells are consistent with the above-described results (Fig.S5.2 a–m).

### KLF15 enhances LDHA transcription and promotes VM development in GBM cells

**Fig. 6:**
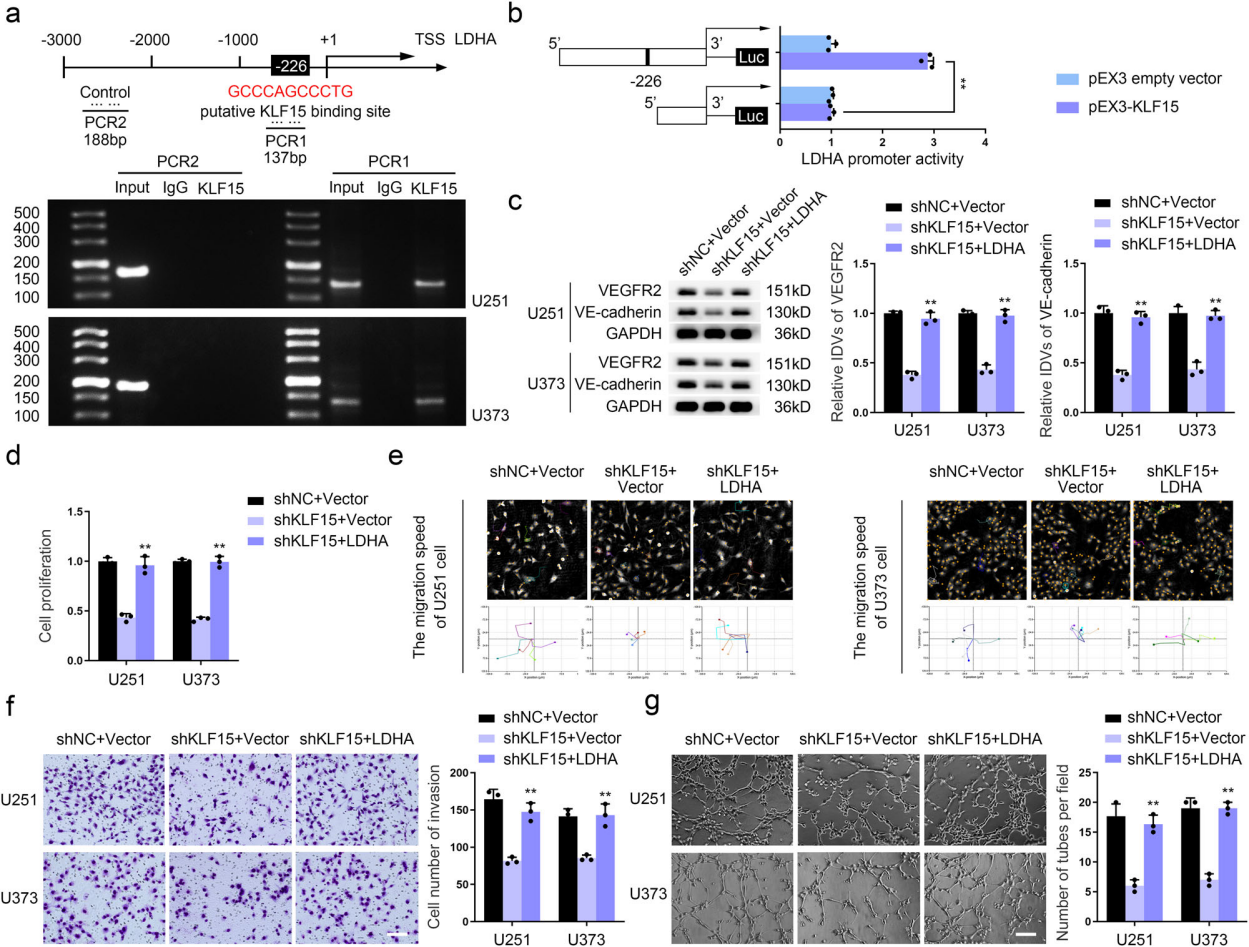
KLF15 enhances LDHA transcription and promotes VM development in GBM cells. **a** ChIP assay detected the binding effect of KLF15 and LDHA promoter region. **b** Schematic depiction of different reporter vectors and relative luciferase activity of LDHA. Data are presented mean ± SD (*n* = 3, each group). **P < 0.01. **c** The VEGFR2 and VE-cadherin protein levels in U251 and U373 cells transfected with the indicated constructs were determined by western blotting. **d** CCK8 assay was used to detect proliferation capacity in U251 and U373 cells transfected with the indicated constructs. **e** The Hstudio M4 system observed the capacity for migration in U251 and U373 cells transfected with the indicated constructs. **f** Transwell method was used to detect the capacity for invasion in U251 and U373 cells transfected with the indicated constructs (×200; scale bar represents 100 μm). **g** Three-dimensional cell culture method was used to detect the change of VM in U251 and U373 cells transfected with the indicated constructs (×200; scale bar represents 100 μm). **P < 0.01, compared with the shKLF15+Vector group. The data are the means ± SD of three independent experiments.

We first predicted the binding sites for KLF15 within the LDHA promoter region using the JASPAR database (https://jaspar.genereg.net/) (Fig. S6a). A chromatin immunoprecipitation (ChIP) assay was used to demonstrate that KLF15 binds directly to the LDHA promoter region (Fig. 6a). Then we measured the relative DNA enrichment of LDHA in KLF15-knockdown GBM cells using ChIP-qPCR (Fig. S6b). The results showed that the specific KLF15 enrichment on the LDHA locus was significantly blunted by KLF15 knockdown. Luciferase reporter assays showed that the LDHA promoter activity of the pEX3-KLF15 group was significantly higher than that of the pEX3 empty vector group (Fig. 6b), indicating transcriptional promotion of KLF15 on LDHA. LDHA could also reverse the inhibition caused by KLF15 deficiency on the VEGFR2 and VE-cadherin protein expression, capacities for proliferation, migration, invasion, and VM of GBM cells (Fig. 6c–g, Fig.S6c–h).

### LDHA promotes VEGFR2 and VE-cadherin lactylation in GBM cells and promotes GBM VM development in vivo

**Fig. 7:**
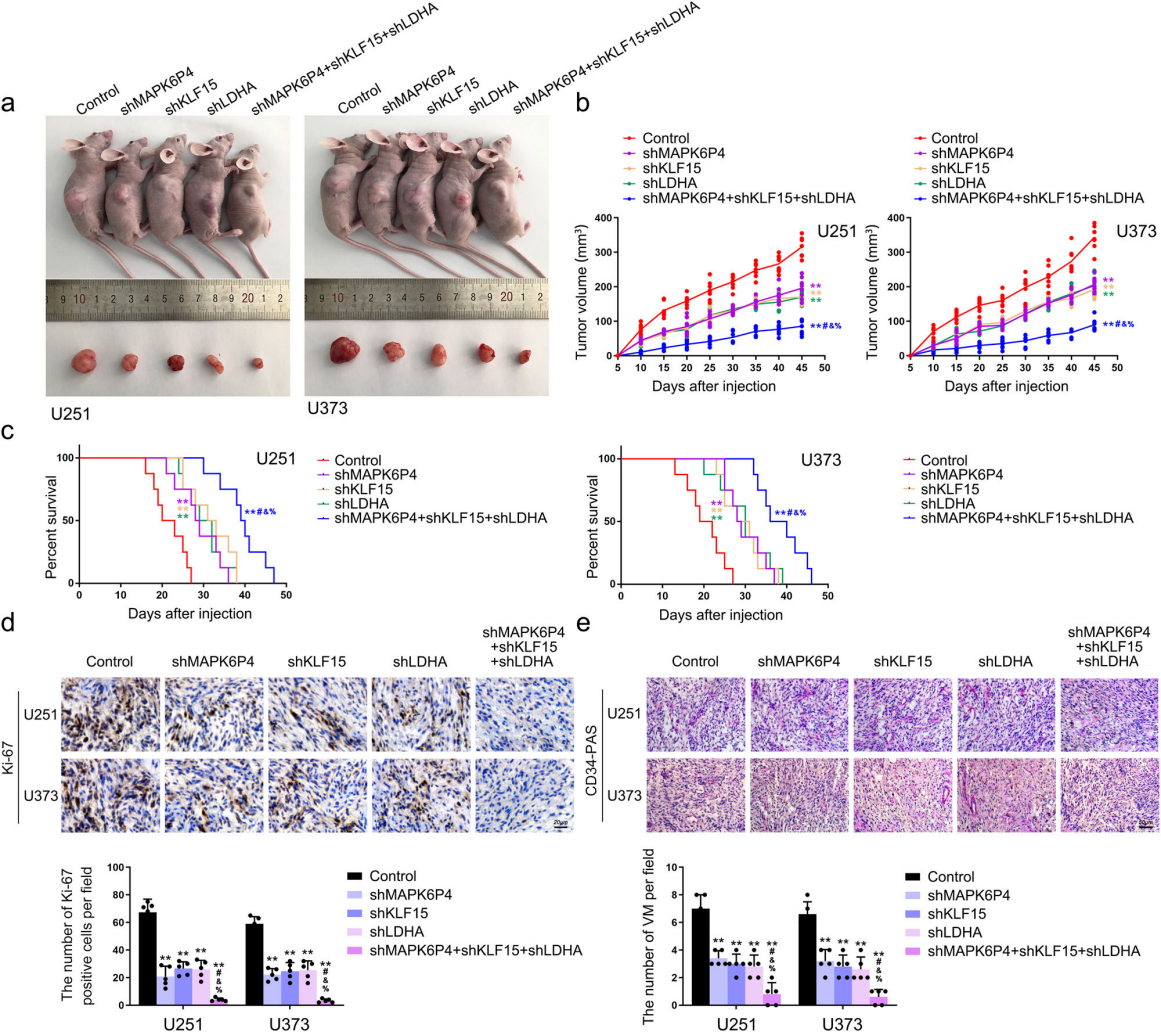
LDHA promotes VEGFR2 and VE-cadherin lactylation in GBM cells and promotes GBM VM development in vivo. **a** The nude mice carrying U251 and U373 cells suspension formed tumors in respective groups are shown. The sample tumors resected from respective groups are shown. **b** Tumor growth curves are shown. Tumor volume was calculated every 5 days after subcutaneous injection of U251 and U373 cell suspensions in nude mice, and the tumor was resected at 45 days. Data are presented as the mean ± SD (*n* = 8, each group). **P < 0.01 compared with Control group, ^##^*P* < 0.01 compared with sh*MAPK6P4* group; ^&^*P* < 0.01 compared with shKLF15 group; ^%^*P* < 0.01 compared with shLDHA group. **c** Kaplan-Meier survival curves of mice bearing orthotopic xenografts (*n* = 8, each group, log-rank test). **P < 0.01 compared with Control group, ^#^*P* < 0.01 compared with sh*MAPK6P4* group; ^&^*P* < 0.01 compared with shKLF15 group; ^%^*P* < 0.01 compared with shLDHA group. **d** Ki-67 immunohistochemical staining in orthotopic xenografts (×400; scale bar represents 20 μm). **e** CD34-PAS double staining was applied to detect the VM in orthotopic xenografts (×200; scale bar represents 50 μm). Data are presented as the mean ± SD (*n* = 5, each group). **P < 0.01 compared with Control group, ^##^*P* < 0.01 compared with sh*MAPK6P4* group; ^&^*P* < 0.05 compared with shKLF15 group; ^%^*P* < 0.05 compared with shLDHA group.

**Fig. 8:**
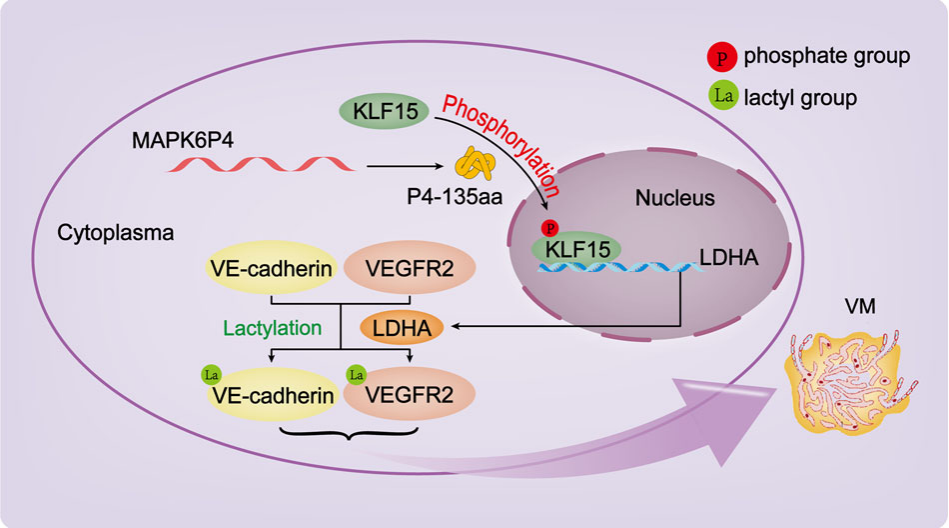
Schematic diagram of the mechanism with which pseudogene *MAPK6P4*-encoded functional peptide promotes GBM VM development. In GBM cells, *MAPK6P4*-encoded peptide P4-135aa promotes KLF15 nuclear entry and upregulates its expression by phosphorylating KLF15. KLF15 directly binds to the promoter region of LDHA and promotes its transcription, which in turn modifies VE-cadherin and VEGFR2 through lactylation, to increase the expression of VE-cadherin and VEGFR2 and promote the development of VM in GBM.

Next, we explored the regulatory effect of LDHA on VEGFR2 and VE-cadherin. While the mRNA expression of VEGFR2 and VE-cadherin after LDHA knockdown did not change significantly (Fig. S7a), LDHA knockdown significantly reduced their protein levels (Fig. 5c). Therefore, we hypothesize that LDHA may regulate the expression of VEGFR2 and VE-cadherin proteins at the post-translational level. We next found that LDHA colocalized with VEGFR2 and VE-cadherin in the cytoplasm by performing IF assays (Fig. S7b), and verified the binding effect of LDHA to VEGFR2 and VE-cadherin by performing co-IP assays (Fig. S7c). Interestingly, knockdown of LDHA strongly decreased VEGFR2 and VE-cadherin protein levels in the lactylation antibody (Kla) immunocomplex (Fig. S7d). Finally, we examined the role of *MAPK6P4*, KLF15, and LDHA deficiency using subcutaneous and orthotopic xenograft nude mouse models of U251 and U373 cells. The sh*MAPK6P4* + shKLF15 + shLDHA group had the best tumor suppressive effect in vivo, with the longest survival time (Fig. 7a–c). Consistently, IHC analysis revealed weak Ki-67 staining in the sh*MAPK6P4*, shKLF15, shLDHA, and especially in the sh*MAPK6P4* + shKLF15 + shLDHA groups (Fig. 7d). CD34-PAS staining showed that VM number significantly decreased in the sh*MAPK6P4*, shKLF15, shLDHA, and especially in the sh*MAPK6P4* + shKLF15 + shLDHA groups (Fig. 7e). A schematic diagram of the mechanism with which pseudogene *MAPK6P4*-encoded functional peptide promotes GBM VM development is provided in Fig. 8.

## Discussion

In recent years, ncRNAs have become the core of human genome research^19^. Dysregulation of ncRNAs can lead to a variety of human diseases, including cancer^20^. There are many types of ncRNA, including microRNA (miRNA), long non-coding RNA (lncRNA), pseudogenes, and circular RNA^21, 22^. With the popularization of proteomics and translation technology, and improvements in the precision and accuracy of associated technology, many ncRNAs have been confirmed to be translatable^23^. Functional peptides are usually encoded by the short open reading frame (sORF) of ncRNA^24–26^. The ORF is a nucleic acid sequence that starts from ATG (or AUG in RNA) and continues with a three-base set to a stop codon^27^. Ribosome analysis has shown that thousands of unannotated ORFs are translated into different species and that longer ORFs are most likely to be encoded^28–32^. Overexpression of a complete ORF can lead to apoptosis of liver cancer cells^33^. The functional peptides encoded by sORFs play an important role in regulating tumor energy metabolism, epithelial to mesenchymal transformation of cancer cells, stability of c-Myc oncoprotein, and ubiquitination and degradation of proliferating cell nuclear antigen, but the function of pseudogenes at the protein level remains unclear^15,34,35^. Recent reports have discovered that a small number of pseudogenes also retain or restore their protein-coding properties and the resulting pseudogene proteins/peptides function in tumorigenesis^36–39^. For example, in U2OS cells, the pseudogene UBB4 can encode functional ubiquitin variations^40^. In the present study, we determined that the pseudogene *MAPK6P4* is highly expressed in GBM cells, whereas the expression of its parental gene MAPK6 in GBM cells was not significantly different from that in normal human astrocytes (NHAs). *MAPK6P4* encodes the 135 amino acid functional polypeptide P4-135aa with an S_TKc domain via ORF12. Here, we confirmed that P4-135aa exerts kinase activity, however, the key residues in the kinase active site still need to be validated through additional mutational analysis, and we will continue to conduct in-depth research in this area in the future. Our study shows that, P4-135aa phosphorylates KLF15, promotes its nuclear entry, and upregulates its expression, thereby promoting proliferation, migration, invasion, and VM development of GBM.

Krüppel-like factors (KLFs) are transcription factors that are involved in many biological processes, including proliferation, apoptosis, differentiation, and development. The biological role of KLFs has received extensive attention^41^. KLF15 is highly expressed in colorectal cancer tissue and promotes tumor growth^42^. KLF15 can promote the transcription of SLC27A1 and KLF3^43,44^; and TTN-AS1 promotes the proliferation and invasion of colorectal cancer through the miR-376a-3p/KLF15 axis^42^. In the present study, we determined that KLF15 was highly expressed in GBM tissue and cells. Mass spectrometry analysis revealed that KLF15 had a phosphorylation site at S238. After mutation of S238A, KLF15 phosphorylation was reduced, also resulted in reduced KLF15 expression levels and inhibited KLF15 entry into the nucleus, and significantly reduced development of VM in GBM. Other studies have made similar observations, phosphorylation of transcription factors can increase their stability and upregulate their expression^45^. Transcription factors in the cytoplasm can be activated by phosphorylation, which causes them to move to the nucleus, where they play a role in transcription regulation^46^. Phosphorylation of STAT3 in liver cancer cells causes it to be transported into the nucleus and promotes the malignant progression of liver cancer^47^. Therefore, we concluded that phosphorylation of KLF15 S238 is necessary to maintain the stability of KLF15 and nuclear import of KLF15, and P4-135aa promotes the development of VM through phosphorylation of the KLF15 S238 site.

LDHA is a key enzyme that converts pyruvate to lactic acid during glycolysis^48–50^. Studies have shown that LDHA is strongly associated with various human cancers, including pancreatic, head and neck, gastric, prostate, breast, hepatocellular carcinoma, and cervical cancer, etc^51–54^. Inhibition of LDHA reduces proliferation, migration, invasion, angiogenesis and cancer metastasis of various cancer cells. Furthermore, LDHA inhibition increases the sensitivity of resistant cancer cells to chemotherapy and radiation therapy^55^. The concept of lactylation (la) was first proposed in 2019, LDHA in M1 macrophages is involved in regulating lactylation and thus changing the polarization state of macrophages^56^. Lactylation is attracting attention as a new post-translational modification that can regulate cell function, and may therefore play an important role in many cancers^57^. In the present study, we confirmed that LDHA promoted VEGFR2 and VE-cadherin lactylation in GBM cells, upregulated their protein expression, and promoted GBM proliferation, migration, invasion, and VM development. Further investigations utilizing mass spectrometry and site mutation assessment are needed to reveal the specific lactylation sites in VEGFR2 and VE-cadherin that are involved in GBM. Orthotopic and subcutaneous xenografted nude mouse models of GBM revealed that knockdown of *MAPK6P4*, KLF15, LDHA, and S238A mutations inhibited the growth of transplanted tumors, prolonged the survival time of nude mice, and inhibited VM formation in transplanted tumors. *MAPK6P4*, KLF15, and LDHA joint knockdown had the best antitumor effect in vitro, and the nude mice bearing joint knockdown tumors had the longest survival time.

In conclusion, in the present study, we found that the *MAPK6P4*-encoded peptide P4-135aa promotes KLF15 nuclear entry and upregulates its expression by phosphorylating KLF15. KLF15 directly binds to the promoter region of LDHA and promotes its transcription, which in turn modifies VE-cadherin and VEGFR2 through a newly discovered post-translational modification, lactylation, to increase the expression of VE-cadherin and VEGFR2 and promote the development of VM in GBM. This study began with the innovative functional study of the pseudogene *MAPK6P4*, which encodes P4-135aa. This research reveals a new molecular mechanism by which the pseudogene *MAPK6P4* regulates VM development in GBM, and also provides new targets for the treatment of glioma in the future.

## Methods

### Clinical specimens

All human glioma specimens and NBTs were obtained from the Department of Neurosurgery of the Shengjing Hospital, China Medical University. All specimens were immediately frozen and preserved in liquid nitrogen. Two experienced clinical pathologists classified glioma specimens into low-grade glioma (LGG, WHO grades I–II) and high-grade glioma (HGG, WHO grades III–IV). All participants signed and provided informed consent, and this study was approved by the Institutional Review Board of the Shengjing Hospital of China Medical University.

### Cell culture and selection

An NHA cell line, as well as human GBM cell lines U251 and U373, were purchased from Shanghai Institutes for Biological Sciences Cell Resource Center and authenticated by short tandem repeat DNA profiling. NHA was grown in RPMI-1640 culture medium (Gibco, Grand Island, NY, USA) with 10% fetal bovine serum (FBS, Gibco, Carlsbad, CA, USA). Human glioma cells and human embryonic kidney (HEK) 293 T cells were grown in Dulbecco’s modified Eagle medium (DMEM) with 10% FBS. Primary patient-derived glioblastoma (PPDG) cells were cultured as follows: Glioblastoma samples were collected within 10 min after surgery and immediately transported to the laboratory under sterile conditions. Then, within 30 min, the tissue was dissociated into single cells using 2% collagenase IV and 0.15% trypsin lyophilized powder. Subsequently, the single cells were cultured on DMEM containing 10% FBS in poly-l-lysine (#P1524-25MG; Sigma) tissue culture plates. All cells were maintained in a humidified incubator at 37 °C with 5% CO_2_. All experiments were performed with mycoplasma-free cells.

### In vitro kinase assay

Recombinant GST-tagged wild-type, sited-mutant KLF15, and GST-tagged P4-135aa were produced in *E. coli* and purified using glutathione-Sepharose beads (Beyotime Institute of Biotechnology, Jiangsu, China). Recombinant proteins were incubated in kinase buffer (20 mM Tris–HCl, pH 7.4, 20 mM NaCl, 10 mM MgCl2, and 1 mM DTT) supplemented with 50 mM ATP (Beyotime Institute of Biotechnology, Jiangsu, China) and 5 μCi [γ-32P] ATP (PerkinElmer, MA, USA) for 20 min at 30 °C in the presence of 500 ng of active P4-135aa. The reaction products were analyzed by SDS-PAGE, transferred to PVDF membranes (Millipore, MA, USA), and autoradiographed. Finally, the PVDF membranes were stained with Coomassie Brilliant Blue Fast Staining Solution (Solarbio, Beijing, China).

### Cell proliferation assay

Cell Proliferation was evaluated by Cell Counting Kit-8 (CCK-8, Beyotime Institute of Biotechnology, China). Cells were seeded in 96-well plates at the density of 2000 cells per well. After 72 h, 10 μl CCK-8 solution was added and cells were incubated at 37 °C for 2 h. The absorbance was measured at 450 nm on the SpectraMax M5 microplate reader (Molecular Devices, USA).

### Cell migration assay

Cell migration assays were performed using the HoloMonitor M4 culture system (Phase Holographic Imaging PHI AB, SE) according to the manufacturer’s protocols. Cells were seeded in a six-well plate, and then placed on the HoloMonitor M4 culture system after being adhered to the wall, and photographed every hour for 6 h. Select five cells in each experimental group for tracking. We showed the last image frame and the cell movements were displayed in spatial *X*–*Y* plots.

### Cell invasion assay

Polycarbonate membrane (Costar, Corning, NY, USA) with an 8 μm pore size was pre-coated with Matrigel and incubated at 37 °C for 30 min. The cells were resuspended in serum-free medium at a density of 2 × 10^5^ cells/ml and inoculated into the upper chamber of the polycarbonate membrane, put 600 μl of 10% FBS medium into the lower chamber. After culturing at 37 °C for 36 h, the cells invaded the lower surface of the membrane from the upper chamber, fixed with methanol and glacial acetic acid at a ratio of 3:1, and stained with Giemsa. Five fields of view were randomly selected for counting and photographed under the microscope.

### Cells VM formation assay

Add 100 μl Matrigel Basement Membrane Matrix (BD Biosciences, Bedford, MA, USA) to a 96-well culture plate and place it in an incubator at 37 °C for 30 min. The cells were resuspended in 100 μl serum-free medium at a density of 6 × 10^5^ cells/ml, inoculated on the surface of Matrigel and incubated for 8 h. Observe the blood vessel structure and take pictures. Calculate the total number of tubular structures in each image.

### Immunofluorescence (IF) assays

Cell slides were fixed with paraformaldehyde in the dark for 30 min and washed three times with PBST. After penetrating the membrane with Trixton-100 for 10 min, the slides were washed with PBST. After blocking with 5% BSA for 2 h, it was incubated with the primary antibody overnight. After reheating at room temperature for half an hour, wash the primary antibody with PBST and then apply its corresponding secondary antibody at room temperature for 2 h. Wash three times with PBST for 10 min each time. The nuclei were stained with DAPI for 5 min. After the staining was completed, PBST was washed three times, and the slides were sealed with 50% glycerol. Observe and take pictures under a confocal microscope. Antibodies used are provided in Supplementary Table 4.

### Chromatin immunoprecipitation (ChIP) assays

ChIP assays were performed using the Simple ChIP Enzymatic Chromatin IP Kit (Cell Signaling Technology, Danvers, MA, USA) according to the manufacturer’s protocols. The cells were cross-linked with EBM-2 containing 1% formaldehyde and collected in a lysis buffer containing 1% PMSF. The chromatin was digested by micrococcal nuclease, and 2% aliquot of the lysate was used as an input control. The lysate was incubated with 3 μg anti-KLF15 antibody (ab167192, Abcam) or normal rabbit IgG, and then immunoprecipitated with protein G agarose beads and incubated overnight at 4 °C with gentle shaking. The DNA cross-linking was reversed by adding 5 mol/L NaCl and proteinase K to react at 65 °C for 2 h, and finally the DNA was purified. Use specific primers to amplify immunoprecipitated DNA by qRT-PCR. The primers are provided in Supplementary Table 1. Relative enrichment of DNA was quantified by ChIP-qPCR analysis of DNA immunoprecipitation.

### Xenograft mouse model in vivo

Female 4-week-old BALB/c nude mice were purchased from HFK Biosciences (Beijing, China). The stably transfected GBM cells (U251 and U373) were xenografted into immunodeficient nude mice for in vivo experiments. All experiments were performed strictly in accordance with the Animal Welfare Act and were approved by the Ethics Committee of China Medical University. For the subcutaneous tumor model, each nude mouse was injected with 6 × 10^5^ cells subcutaneously on the right abdomen. The tumor was measured every 7 days and calculated according to the formula: volume (mm^3^) = length × width^2^/2. After indicated days, the mice were sacrificed and the tumors were isolated. For orthotopic inoculations, cells were injected into the right striatum of nude mice, and the number of surviving nude mice was recorded and the Kaplan-Meier survival curve was used for survival analysis.

### Immunohistochemistry (IHC)

Tissue sections were prepared according to the Ultrasensitive SP kit (Fuzhou MaiXin Biotech, Fujian, China). The sections were then incubated with primary antibodies overnight at 4 °C, and the secondary antibody was incubated with the samples for 30 min at 37 °C. This was followed by visualization using a DAB kit (Fuzhou MaiXin Biotech, China). The antibodies used are listed in Supplementary Table 4.

### CD34 endothelial marker periodic acid-Schiff dual staining (CD34-PAS)

IHC was performed using rabbit anti-human CD34 primary monoclonal antibody (Beijing Zhongshan Golden Bridge, China). The slides were then exposed to periodic acid solution for 10 min, incubated with Schiff solution for 10 min in the dark, and counterstained with Mayer’s hematoxylin (Zhuhai Baso, Guangdong, China). Finally, the slides were viewed under a light microscope to detect CD34 and PAS signals, and the pipe structure of CD34(−) + PAS (+) was counted.

### Statistics and reproducibility

Statistical analysis was performed using Student’s *t* test or one-way ANOVA in GraphPad Prism7, and data are presented as the mean ± standard deviation (SD). *P* < 0.05 was considered significant. The number of samples per independent experiment is described in the legends.

### Other methods

qRT-PCR assay, cell transfection, western blot assay and co-IP assay were performed according to the manufacturer’s protocol. See Supplementary Materials and Methods for further details. The target sequences and vectors of plasmids are shown in Supplementary Table 2 and Supplementary Table 3.

### Reporting summary

Further information on research design is available in the Nature Portfolio Reporting Summary linked to this article.

### Supplementary information

Supplementary Information

Description of Additional Supplementary Files

Supplementary Data 1

Reporting Summary

Uncropped original western blots

## Data Availability

The numerical data that make up the graphs in the paper are shown in Supplementary Data 1. All other data that support the findings of this study are available from the corresponding author upon reasonable request.
