# Supplementary material for: Pseudogene MAPK6P4-encoded functional peptide promotes glioblastoma vasculogenic mimicry development
Source: Commun Biol. 2023 Oct 18;6:1059. doi: 10.1038/s42003-023-05438-1 (PMC10584926; doi:10.1038/s42003-023-05438-1)
Supplement: Supplementary file 1 — Supplementary Information [file 42003_2023_5438_MOESM1_ESM.docx]

**Supplementary Information**

**Pseudogene *MAPK6P4*-encoded Functional Peptide Promotes Glioblastoma Vasculogenic Mimicry Development**

This file includes:

Supplementary Figures

Supplementary Materials and Methods

Supplementary Tables

**Supplementary Figure**

**Supplementary figure 1.1**

**
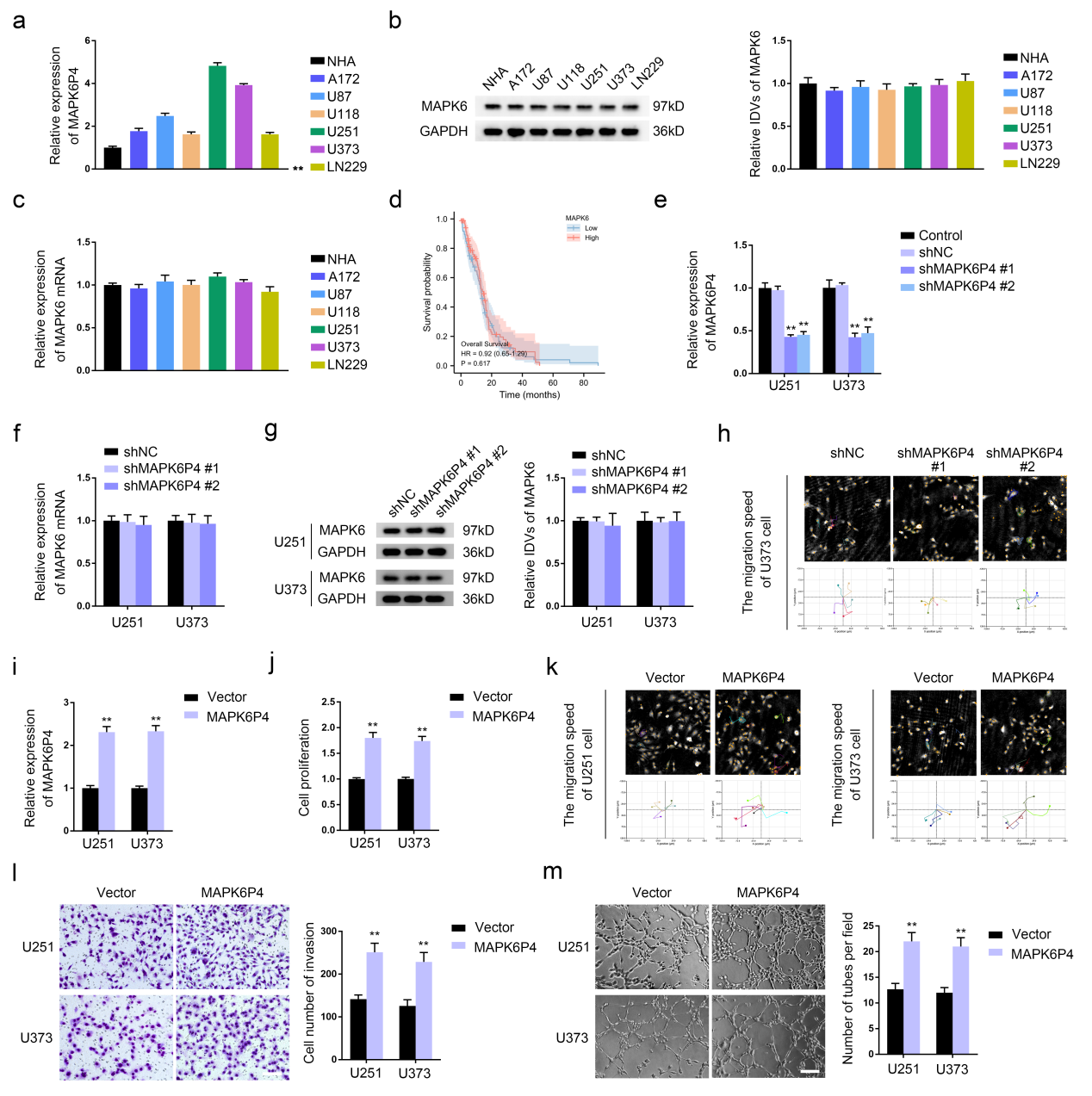
**

**Figure S1.1 *MAPK6P4* overexpression promotes VM in GBM cells. Related to Figure 1.** (a) qRT-PCR was used to detect the *MAPK6P4* expression level in NHA, A172, U87, U118, U251, U373 and LN229 cells. (b-c) qRT-PCR and western blot were used to detect the mRNA and protein expression of MAPK6 in NHA, A172, U87, U118, U251, U373 and LN229 cells. (d) Kaplan-Meier OS analysis of MAPK6 expression in patients with GBM (p = 0.617, log-rank test). (e) The *MAPK6P4* knockdown efficiency was verified in U251 and U373 cells by qRT-PCR. (f, g) qRT-PCR and western blot were used to detect the expression of mRNA and protein expression of MAPK6 in U251 and U373 cells with *MAPK6P4* knockdown. (h) The Hstudio M4 system observed the capacity for migration in U373 cells with *MAPK6P4* knockdown. (i) The *MAPK6P4* overexpression efficiency was verified in U251 and U373 cells by qRT-PCR. (j) CCK8 assay was uesd to detect proliferation capacity of U251 and U373 cells with *MAPK6P4* overexpression. (k) The Hstudio M4 system observed the capacity for migration in U251 cells with *MAPK6P4* overexpression. (l) Transwell method was used to detect the capacity for invasion in U251 and U373 cells with *MAPK6P4* overexpression (×200; scale bar represents 100 μm). (m) Three-dimensional cell culture method was used to detect the change of VM in U251 and U373 cells with *MAPK6P4* overexpression (×200; scale bar represents 100 μm). ***P* < 0.01, compared with corresponding shNC/Vector group. The data are the means±SD of three independent experiments.

**Supplementary figure 1.2**


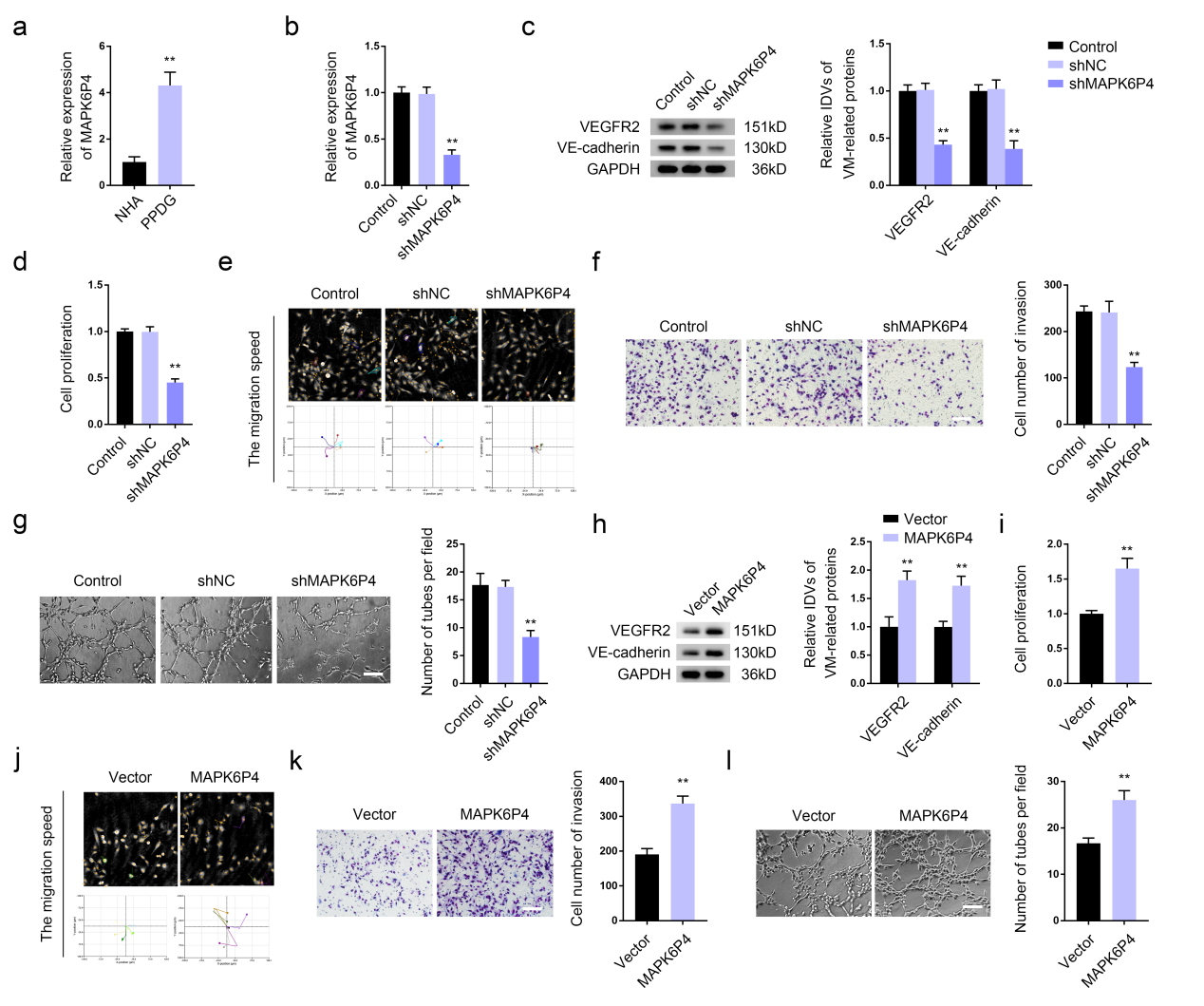


**Figure S1.2 *MAPK6P4* promotes VM in PPDG cells. Related to Figure 1.** (a) qRT-PCR was used to detect the *MAPK6P4* expression level in NHA and PPDG cells, ***P* < 0.01 compared with NHA group. (b) The *MAPK6P4* knockdown efficiency was verified in PPDG cells by qRT-PCR. (c) The VEGFR2 and VE-cadherin protein levels in PPDG cells with *MAPK6P4* knockdown were determined by western blot. (d) CCK8 assay was uesd to detect proliferation capacity of PPDG cells with *MAPK6P4* knockdown. (e) The Hstudio M4 system observed the capacity for migration in PPDG cells with *MAPK6P4* knockdown. (f) Transwell method was used to detect the capacity for invasion in PPDG cells with *MAPK6P4* knockdown (×200; scale bar represents 100 μm). (g) Three-dimensional cell culture method was used to detect the change of VM in PPDG cells with *MAPK6P4* knockdown (×200; scale bar represents 100 μm). (h) The VEGFR2 and VE-cadherin protein levels in PPDG cells with *MAPK6P4* overexpression were determined by western blot. (i) CCK8 assay was uesd to detect proliferation capacity of PPDG cells with *MAPK6P4* overexpression. (j) The Hstudio M4 system observed the capacity for migration in PPDG cells with *MAPK6P4* overexpression. (k) Transwell method was used to detect the capacity for invasion in PPDG cells with *MAPK6P4* overexpression (×200; scale bar represents 100 μm). (l) Three-dimensional cell culture method was used to detect the change of VM in PPDG cells with *MAPK6P4* overexpression (×200; scale bar represents 100 μm). ***P* < 0.01, compared with corresponding shNC/Vector group. The data are the means±SD of three independent experiments.

**Supplementary figure 2.1**

**
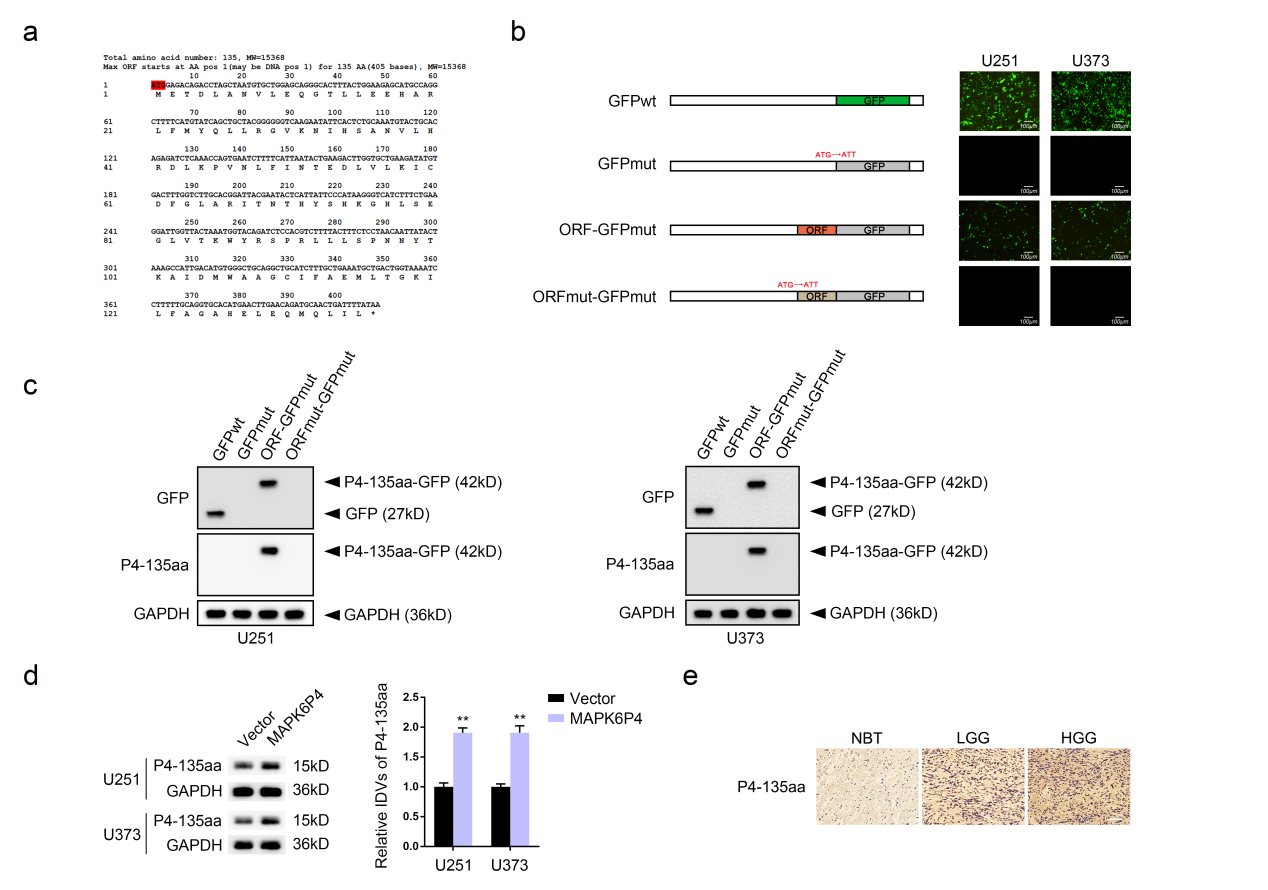
**

**Figure S2.1 *MAPK6P4* promotes VM development in GBM cells via the encoded peptide P4-135aa. Related to Figure 2.** (a) The nucleotide sequence of *MAPK6P4*-ORF12 and amino acid sequence of human *MAPK6P4* ORF12-encoded polypeptide. ATG codons region are highlighted in red. (b) Constructs were transfected into the U251 and U373 cells. Then, at 24 h after transfection, the GFP fluorescence was detected using a fluorescence microscope (scale bars, 100 μm). (c) The expression of the fusion protein was detected by western blot with anti-GFP and P4-135aa antibody in U251 and U373 cells. (d) Western blot was used to detect the expression of P4-135aa in U251 and U373 cells with ORF expression. Each value represents the mean±SD (n = 3), ***P* < 0.01, compared with the Vector group. (e) IHC shows the expression and distribution of P4-135aa in NBT, LGG and HGG (×200; scale bar represents 150 μm).

**Supplementary figure 2.2**


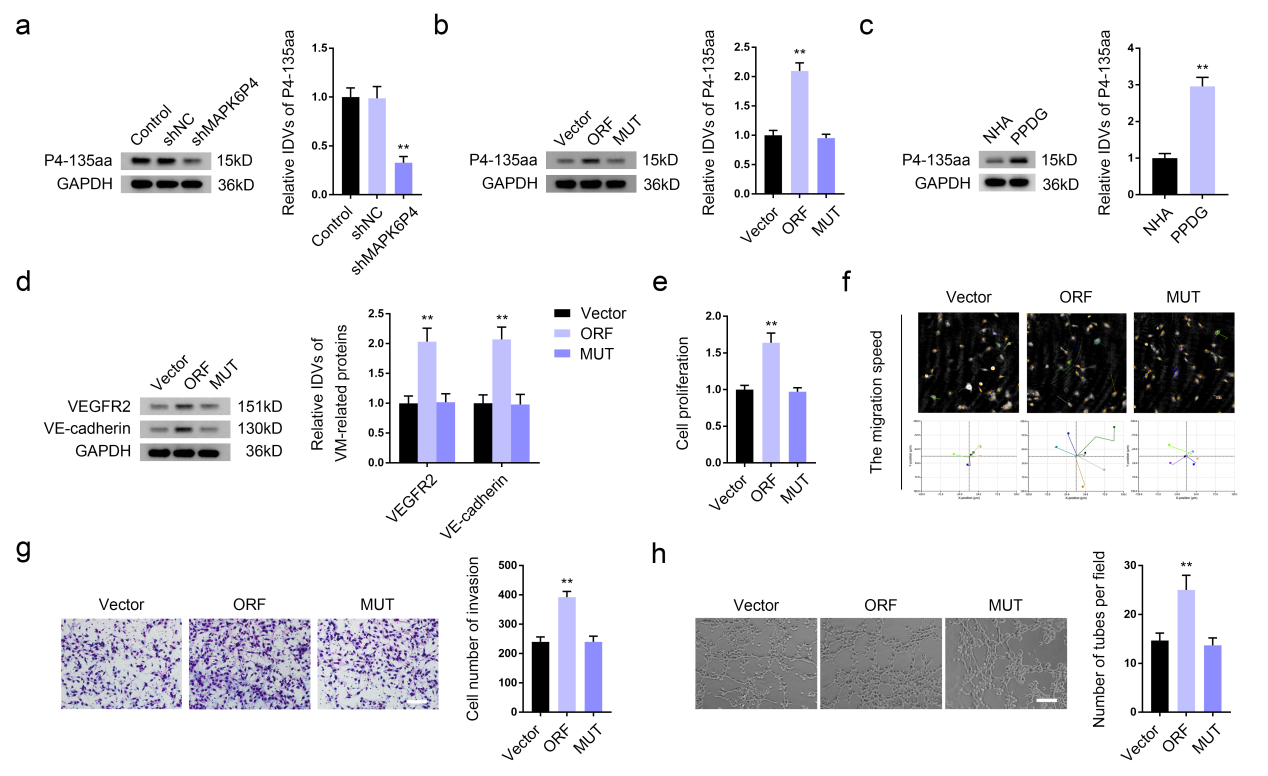


**Figure S2.2 *MAPK6P4* promotes VM development in PPDG cells via the encoded peptide P4-135aa. Related to Figure 2.** (a) Western blot was used to detect the expression of P4-135aa in PPDG cells with *MAPK6P4* knockdown. (b) Western blot was used to detect the expression of P4-135aa in PPDG cells transfected with the indicated constructs. Vector, empty vector; ORF, *MAPK6P4* ORF12; MUT, start codon ATG of *MAPK6P4* ORF12 was mutated to ATT. (c) Western blot was used to detect the expression of P4-135aa in NHA and PPDG cells, ***P* < 0.01 compared with NHA group. (d) The VEGFR2 and VE-cadherin protein levels in PPDG cells transfected with the indicated constructs were determined by western blotting. (e) CCK8 assay was uesd to detect proliferation capacity in PPDG cells transfected with the indicated constructs. (f) The Hstudio M4 system observed the capacity for migration in PPDG cells transfected with the indicated constructs. (g) Transwell method was used to detect the capacity for invasion in PPDG cells transfected with the indicated constructs (×200; scale bar represents 100 μm). (h) Three-dimensional cell culture method was used to detect the change of VM in PPDG cells transfected with the indicated constructs (×200; scale bar represents 100 μm). ***P* < 0.01, compared with corresponding shNC/Vector group.The data are the means±SD of three independent experiments.

**Supplementary figure 3.1**


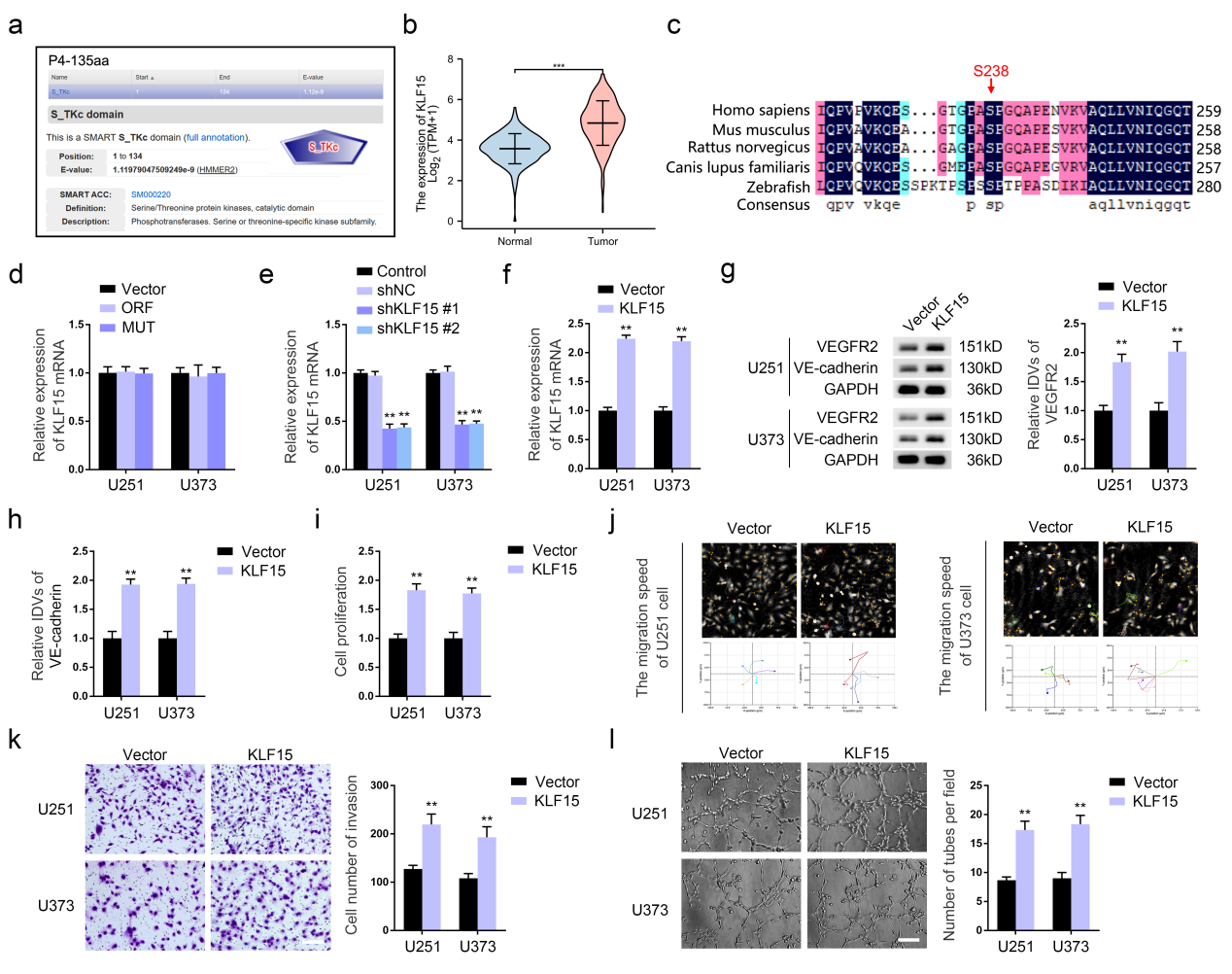


**Figure S3.1 P4-135aa phosphorylates KLF15 at S238, promoting VM formation in GBM cells. Related to Figure 3.** (a) Kinase domains S_TKc was predicted in P4-135aa by SMART database. (b) The expression level of *KLF15* in GBM was analyzed by TCGA database. (c) Alignment of Serine 238 and adjacent amino acids of KLF15 among different species. (d) qRT-PCR was used to detect the expression of KLF15 mRNA in U251 and U373 cells transfected with the indicated constructs. (e) The KLF15 knockdown efficiency was verified in U251 and U373 cells by qRT-PCR. (f) The KLF15 overexpression efficiency was verified in U251 and U373 cells by qRT-PCR. (g, h) The VEGFR2 and VE-cadherin protein levels in U251 and U373 cells with KLF15 overexpression were determined by western blot. (i) CCK8 assay was uesd to detect proliferation capacity of U251 and U373 cells with KLF15 overexpression. (j) The Hstudio M4 system observed the capacity for migration in U251 cells with KLF15 overexpression. (k) Transwell method was used to detect the capacity for invasion in U251 and U373 cells with KLF15 overexpression (×200; scale bar represents 100 μm). (l) Three-dimensional cell culture method was used to detect the change of VM in U251 and U373 cells with KLF15 overexpression (×200; scale bar represents 100 μm). ***P* < 0.01, compared with corresponding shNC/Vector group. The data are the means±SD of three independent experiments.

**Supplementary figure 3.2**


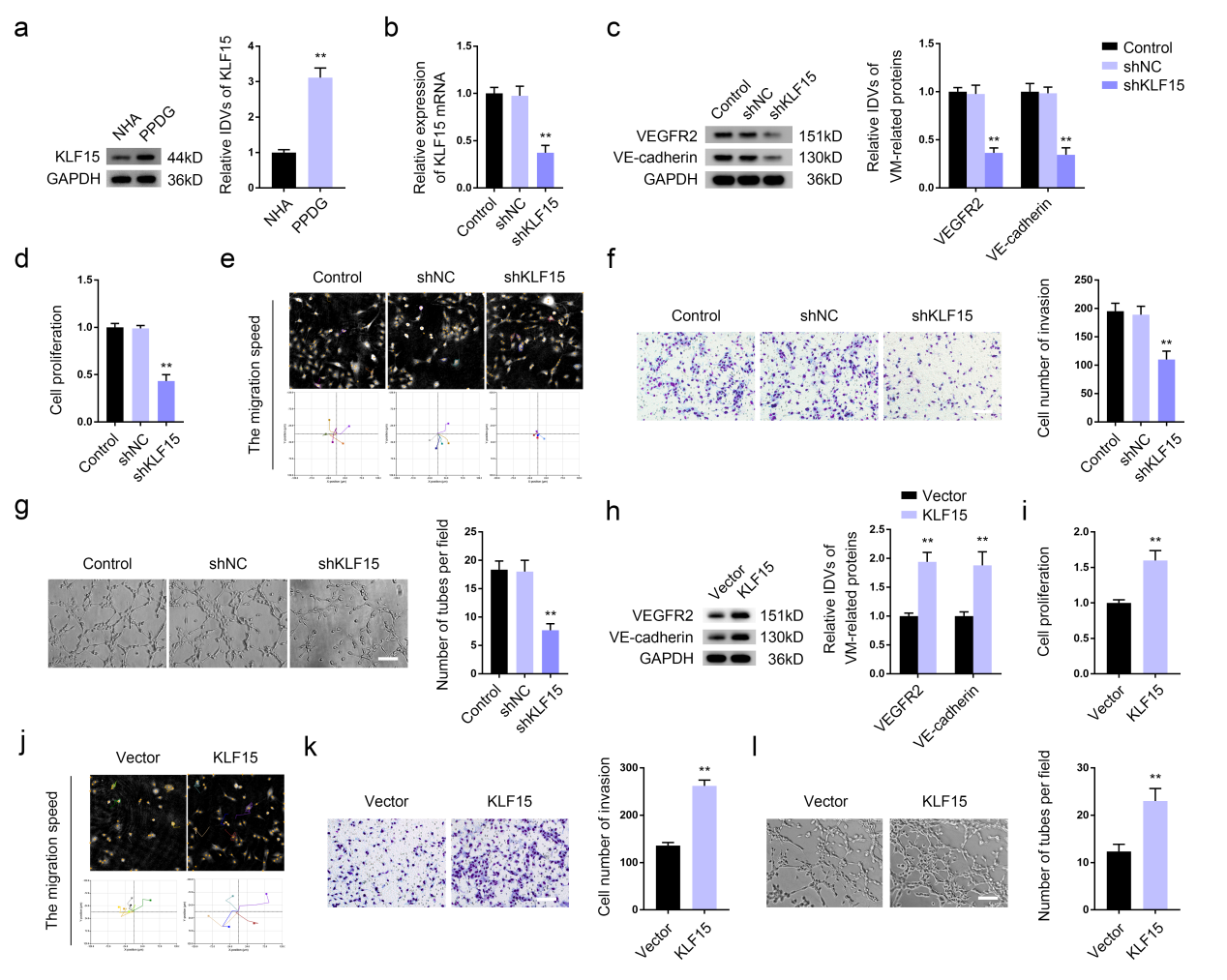


**Figure S3.2 KLF15 promotes VM in PPDG cells. Related to Figure 3.** (a) Western blot was used to detect the expression of KLF15 in NHA and PPDG cells, ***P* < 0.01 compared with NHA group. (b) The KLF15 knockdown efficiency was verified in PPDG cells by qRT-PCR. (c) The VEGFR2 and VE-cadherin protein levels in PPDG cells with KLF15 knockdown were determined by western blot. (d) CCK8 assay was uesd to detect proliferation capacity of PPDG cells with KLF15 knockdown. (e) The Hstudio M4 system observed the capacity for migration in PPDG cells with KLF15 knockdown. (f) Transwell method was used to detect the capacity for invasion in PPDG cells with KLF15 knockdown (×200; scale bar represents 100 μm). (g) Three-dimensional cell culture method was used to detect the change of VM in PPDG cells with KLF15 knockdown (×200; scale bar represents 100 μm). (h) The VEGFR2 and VE-cadherin protein levels in PPDG cells with KLF15 overexpression were determined by western blot. (i) CCK8 assay was uesd to detect proliferation capacity of PPDG cells with KLF15 overexpression. (j) The Hstudio M4 system observed the capacity for migration in PPDG cells with KLF15 overexpression. (k) Transwell method was used to detect the capacity for invasion in PPDG cells with KLF15 overexpression (×200; scale bar represents 100 μm). (l) Three-dimensional cell culture method was used to detect the change of VM in PPDG cells with KLF15 overexpression (×200; scale bar represents 100 μm). ***P* < 0.01, compared with corresponding shNC/Vector group. The data are the means±SD of three independent experiments.

**Supplementary figure 4.1**


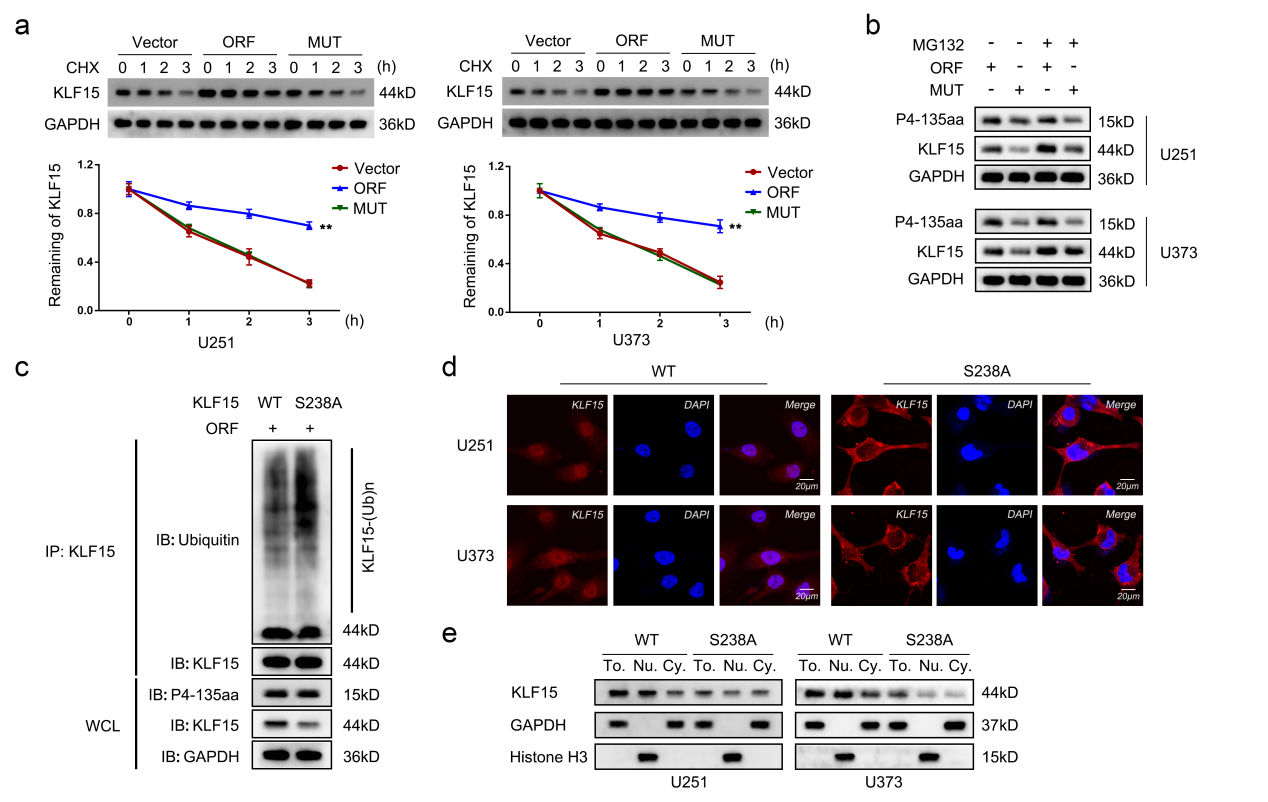


**Figure S4.1 Phosphorylation of KLF15 at S238 inhibites ubiquitination of KLF15 and increases KLF15 translocation to nucleus. Related to Figure 4.** (a, b) Half-life of KLF15 protein was measured in U251 and U373 cells transfected with the indicated constructs after treatment with CHX. ***P* < 0.01, compared with corresponding the Vector group (n = 3). (b) Expression levels of KLF15 in U251 and U373 cells with the indicated constructs after treatment with 20μM MG132 for 8h. (c) IP and IB analysis of KLF15 ubiquitination in HEK-293T cells transfected with the indicated constructs in the presence of MG132. (d) The distribution of KLF15 in U251 amd U373 cells was determined by IF assay. (e) The expression level of KLF15 was determined by western blotting after separating the nucleoplasm of U251 amd U373 cells (To., total cell lysates; Nu., nucleus lysates; Cy., cytoplasmic lysates).

**Supplementary figure 4.2**


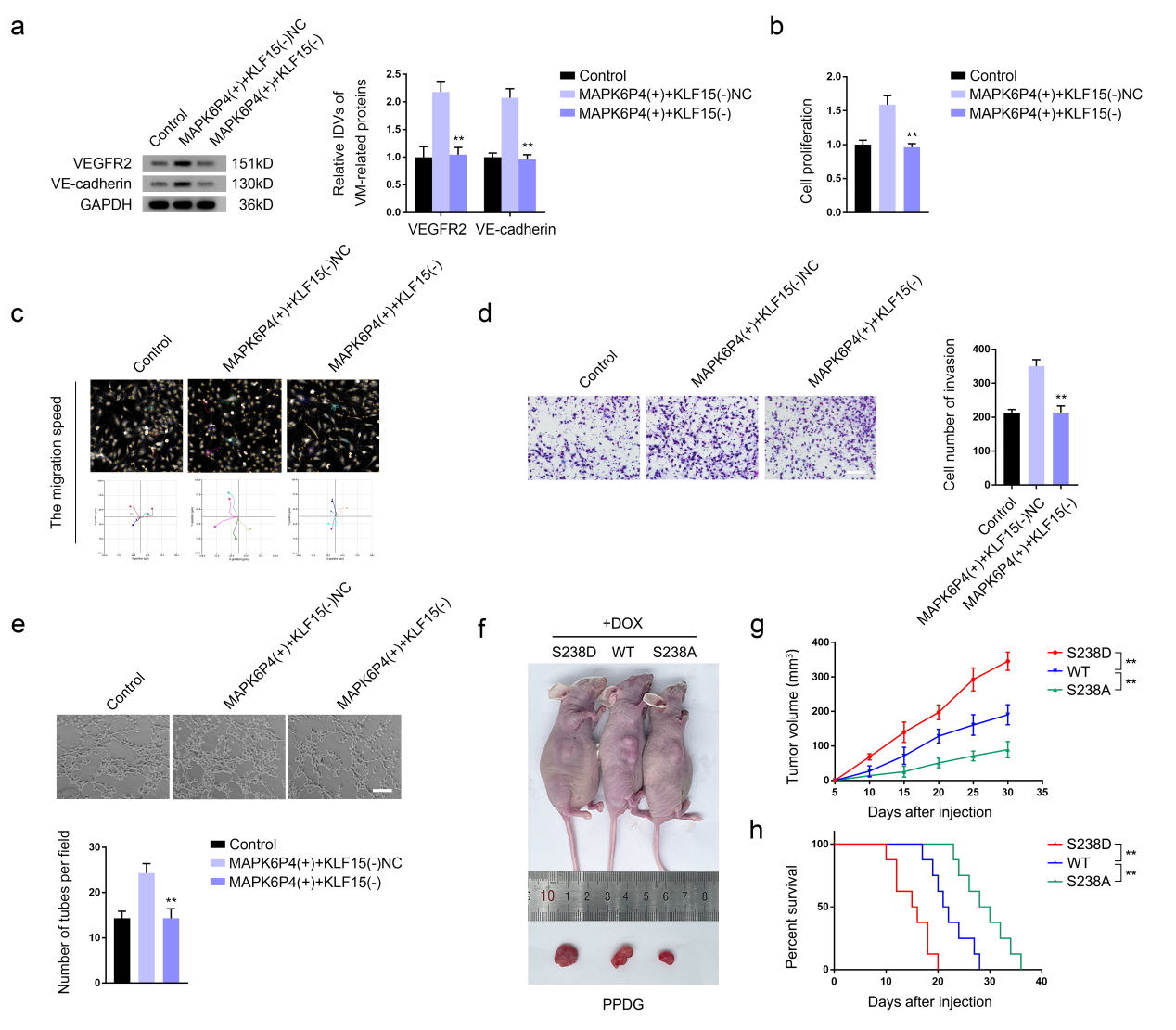


**Figure S4.2 Phosphorylation of KLF15 at S238 increases the stability of KLF15 and promotes GBM VM development *in vivo*. Related to Figure 4.** (a) The VEGFR2 and VE-cadherin protein levels in PPDG cells transfected with the indicated constructs were determined by western blotting. (b) CCK8 assay was uesd to detect proliferation capacity in PPDG cells transfected with the indicated constructs. (c) The Hstudio M4 system observed the capacity for migration in PPDG cells transfected with the indicated constructs. (d) Transwell method was used to detect the capacity for invasion in PPDG cells transfected with the indicated constructs (×200; scale bar represents 100 μm). (e) Three-dimensional cell culture method was used to detect the change of VM in PPDG cells transfected with the indicated constructs (×200; scale bar represents 100 μm). ***P* < 0.01, compared with the *MAPK6P4*(+)+KLF15(-)NC group. The data are the means±SD of three independent experiments. (f) The nude mice carrying PPDG cells suspension formed tumors in respective groups are shown. The sample tumors resected from respective groups are shown. (g) Tumor growth curves are shown. Tumor volume was calculated every 5 days after PPDG cells suspension injection, and the tumor was resected at 30 days. Data are presented as the mean ± SD (n = 8, each group). (h) Survival curves of nude mice PPDG cells suspension injected into the right striatum are shown (n = 8, each group, log-rank test).

**Supplementary figure 5.1**


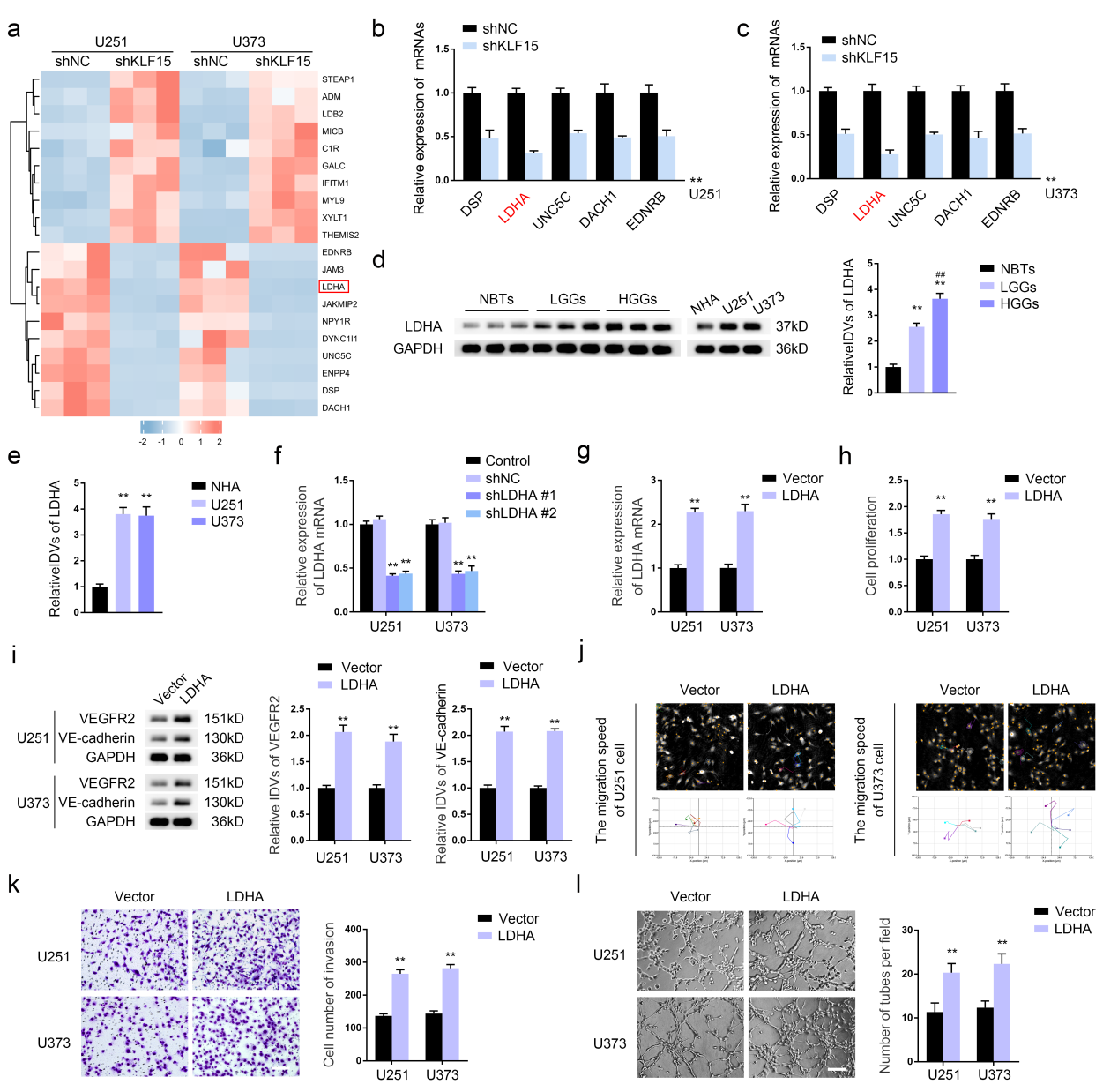


**Figure S5.1. LDHA is a target of KLF15 and can promote VM. Related to Figure 5.** (a) mRNA PCR array screened out the different genes in U251 and U373 knocked down KLF15, and compared with NC group respectively. (b, c) qRT-PCR was used to detect the top5 downregulated mRNA in U251 and U373 cells. (d, e) Western blot was used to detect the expression of LDHA in NBTs, LGGs, and HGGs (left), ***P* < 0.01 compared with NBTs group; ^##^*P* < 0.01 compared with LGGs group (n = 3). Western blot was used to detect the expression of LDHA in NHA, U251 and U373 cells (right), ***P* < 0.01 compared with NHA group (n = 3). (f) The LDHA knockdown efficiency was verified in U251 and U373 cells by qRT-PCR. (g) The LDHA overexpression efficiency was verified in U251 and U373 cells by qRT-PCR. (h) CCK8 assay was uesd to detect proliferation capacity of U251 and U373 cells with LDHA overexpression. (i) The VEGFR2 and VE-cadherin protein levels in U251 and U373 cells with LDHA overexpression were determined by western blotting. (j) The Hstudio M4 system observed the capacity for migration in U251 and U373 cells with LDHA overexpression. (k) Transwell method was used to detect the capacity for invasion in U251 and U373 cells with LDHA overexpression (×200; scale bar represents 100 μm). (l) Three-dimensional cell culture method was used to detect the change of VM in U251 and U373 cells with LDHA overexpression (×200; scale bar represents 100 μm). ***P* < 0.01, compared with corresponding shNC/Vector group. The data are the means±SD of three independent experiments.

**Supplementary figure 5.2**

**
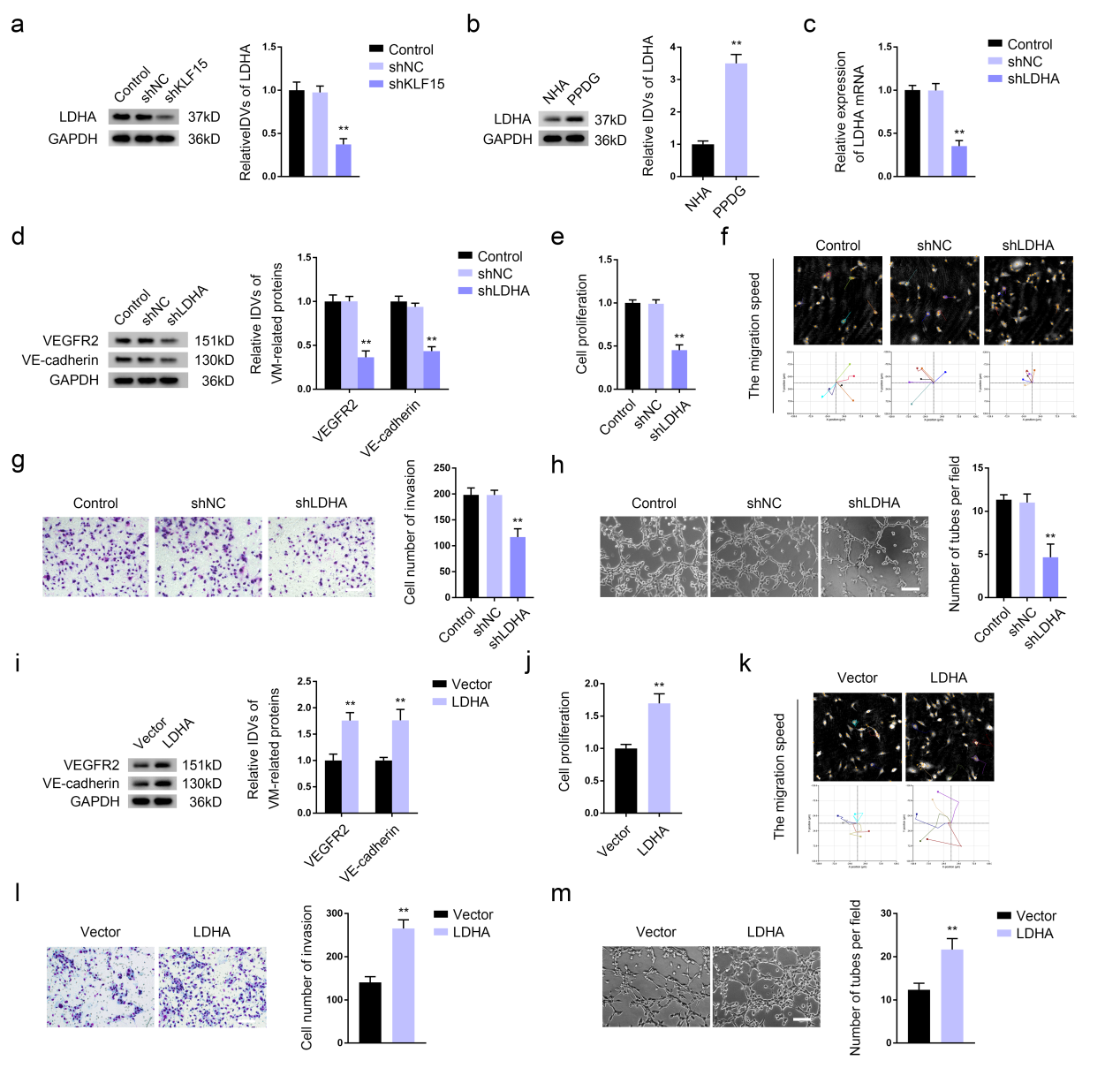
**

**Figure S5.2. LDHA is a target of KLF15 and can promote VM. Related to Figure 5.** (a) The LDHA protein levels in PPDG cells with KLF15 deficiency were determined by western blotting. (b) Western blot was used to detect the expression of LDHA in NHA and PPDG cells, ***P* < 0.01 compared with NHA group. (c) The LDHA knockdown efficiency was verified in PPDG cells by qRT-PCR. (d) The VEGFR2 and VE-cadherin protein levels in PPDG cells with LDHA deficiency were determined by western blotting. (e) CCK8 assay was uesd to detect proliferation capacity of PPDG cells with LDHA deficiency. (f) The Hstudio M4 system observed the capacity for migration in PPDG cells with LDHA deficiency. (g) Transwell method was used to detect the capacity for invasion in PPDG cells with LDHA deficiency (×200; scale bar represents 100 μm). (h) Three-dimensional cell culture method was used to detect the change of VM in PPDG cells with LDHA deficiency (×200; scale bar represents 100 μm). (i) The VEGFR2 and VE-cadherin protein levels in PPDG cells with LDHA overexpression were determined by western blotting. (j) CCK8 assay was uesd to detect proliferation capacity of PPDG cells with LDHA overexpression. (k) The Hstudio M4 system observed the capacity for migration in PPDG cells with LDHA overexpression. (l) Transwell method was used to detect the capacity for invasion in PPDG cells with LDHA overexpression (×200; scale bar represents 100 μm). (m) Three-dimensional cell culture method was used to detect the change of VM in PPDG cells with LDHA overexpression (×200; scale bar represents 100 μm). ***P* < 0.01, compared with corresponding shNC/Vector group. The data are the means±SD of three independent experiments.

**Supplementary figure 6**

**
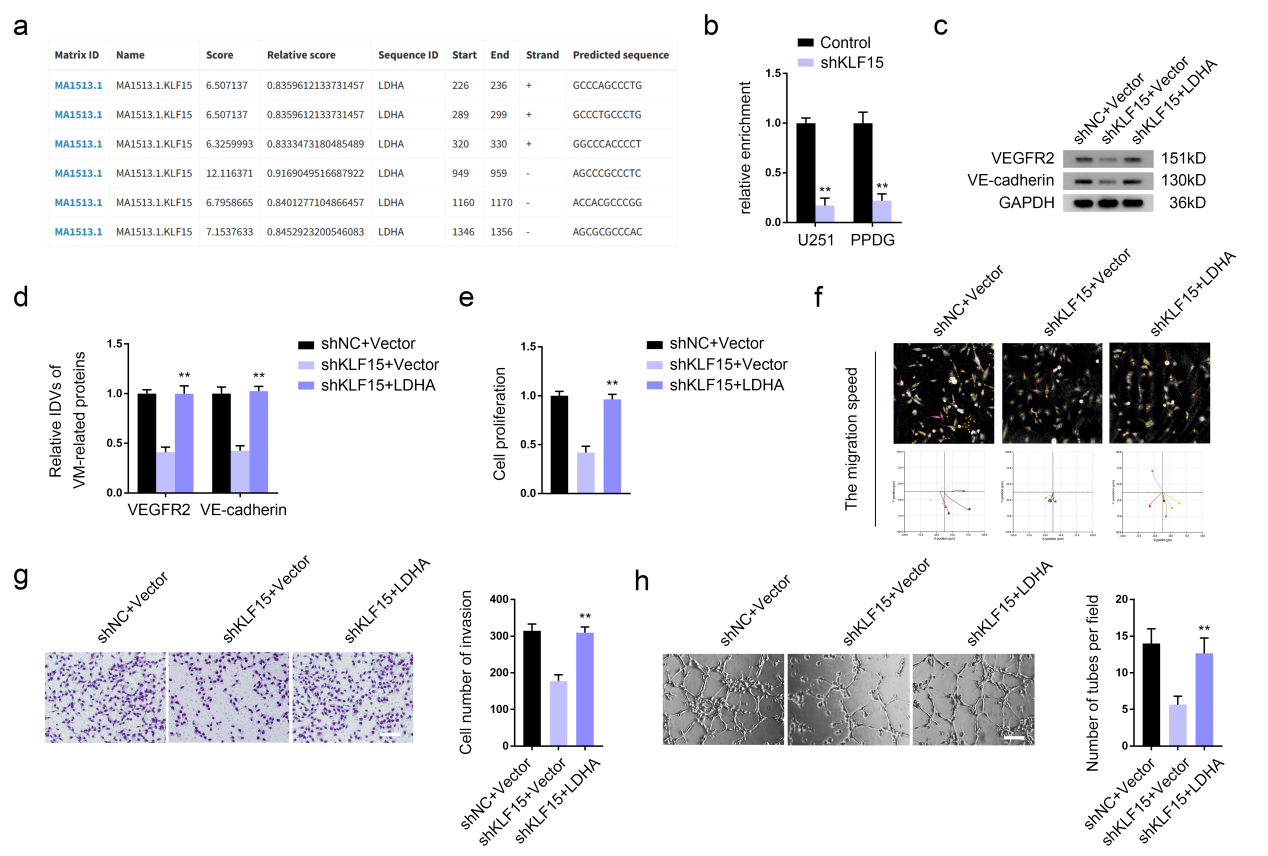
**

**Figure S6. Potential binding site of KLF15 to the LDHA promoter region. Related to Figure 6.** (a) Analysis of potential binding site of KLF15 to the LDHA promoter region by JASPAR database. (b) ChIP-qPCR analysis of DNA immunoprecipitated was used to quantify the relative DNA enrichment of LDHA. ***P* < 0.01, compared with the Control group. The data are the means±SD of three independent experiments. (c, d) The VEGFR2 and VE-cadherin protein levels in PPDG cells transfected with the indicated constructs were determined by western blotting. (e) CCK8 assay was uesd to detect proliferation capacity in PPDG cells transfected with the indicated constructs. (f) The Hstudio M4 system observed the capacity for migration in PPDG cells transfected with the indicated constructs. (g) Transwell method was used to detect the capacity for invasion in PPDG cells transfected with the indicated constructs (×200; scale bar represents 100 μm). (h) Three-dimensional cell culture method was used to detect the change of VM in PPDG cells transfected with the indicated constructs (×200; scale bar represents 100 μm). ***P* < 0.01, compared with the shKLF15+Vector group. The data are the means±SD of three independent experiments.

**Supplementary figure 7**


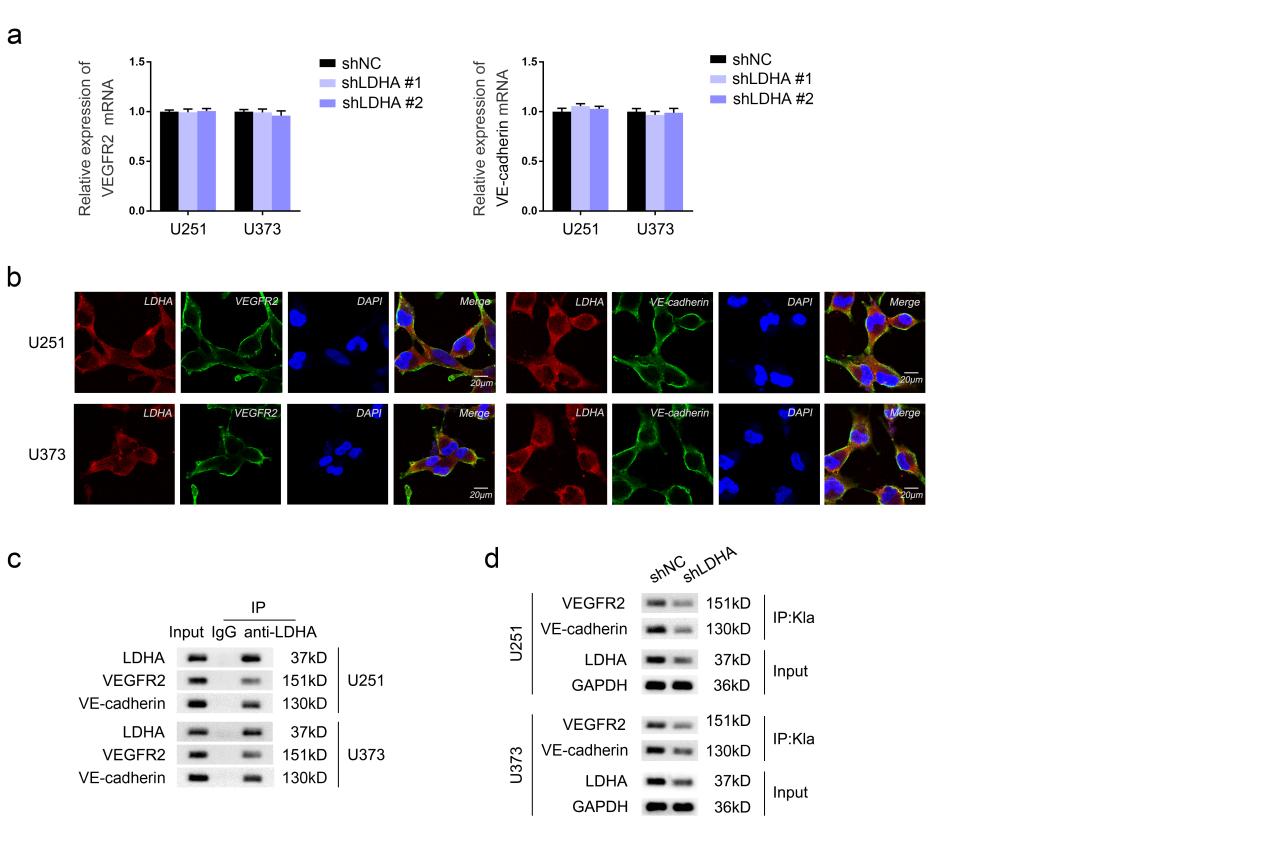


**Figure S7. LDHA promotes VEGFR2 and VE-cadherin lactylation in GBM cells and promotes GBM VM development *in vivo.* Related to Figure 7.** (a) The VEGFR2 and VE-cadherin mRNA levels in U251 and U373 cells with LDHA deficiency were determined by qRT-PCR. (b) Colocalization of LDHA, VEGFR2 and VE-cadherin in U251 and U373 cells was determined by IF staining and observed by laser scanning confocal microscopy. (c) The interaction of LDHA, VEGFR2 and VE-cadherin in U251 and U373 cells were determined by co-IP assays. (d) IP and IB analysis of VEGFR2 and VE-cadherin lactylation in U251 and U373 cells transfected with the indicated constructs.

**Supplementary Materials and Methods**

**Real-Time PCR (qRT-PCR) assay**

Total RNAs were extracted by TRIzol reagent (Life Technologies, Carlsbad, CA, USA). SYBR PrimeScript primary RT-PCR kit (Takara Bio, Beijing, China) was used to evaluate the expression levels of RNA, and the reaction was carried out by the 7500 Fast RT-PCR system (Applied Biosystems, Foster City, CA, USA). The endogenous control was glyceraldehyde 3-phosphate dehydrogenase (GAPDH). Relative expression values were calculated using the relative quantification (2-^△△^Ct) method. The primers are provided in Supplementary Table 1.

**Cell transfection**

Cells were seeded in 24-well plates and transfected using transfection reagent Lipofectamine 3000 (Life Technologies, CA, USA) under approximately 80% fusion conditions according to the instructions. Stable cell lines were selected by geneticin (G418) or purinomycin. After 4 weeks of application, G418-resistant (or purinomycin-resistant) clones were obtained. Plasmids and corresponding empty vectors are constructed by the GenePharma (Shanghai, China). The target sequences and vectors of plasmids are shown in Supplementary Table 2 and Supplementary Table 3.

**Construction of the doxycycline-inducible system**

Firstly, the wild-type KLF15-containing plasmid was altered using the QuikChange site-directed mutagenesis kit (Agilent Technologies) according to manufacturer instructions. The constructed plasmids carrying KLF15-S238D and KLF15-S238A were inserted into the complementary site of pLVX-TetOne-Puro vector by which the mutated KLF15 were expressed under the Lentiviral Tet-One inducible expression system (Clontech, CA, USA). The recombinant vectors containing KLF15-WT, KLF15-S238D and KLF15-S238A coding sequences were transfected into 293T cells to produce lentivirus. PPDG cells were incubated with virus-containing supernatant in the presence of 6µg/ml polybrene for transduction and the transfected PPDG cells were selected by puromycin. The selected PPDG cells were cultured in the medium with 0 or 2 µg/ml of doxycyclineand incubated for 24h, and detected the KLF15 gene expression using qRT-PCR to examine the efficiency of the doxycycline induction system.

**Western blot assay**

The total protein of the cells was extracted with RIPA buffer (Beyotime Institute of Biotechnology, Jiangsu Province) supplemented with protease inhibitors (10 mg/mL aprotinin, 10 mg/mL PMSF and and 50 mM sodium orthovanadate). The protein concentration was then determined using the BCA protein assay kit (Jiangsu Beiyang Institute of Biotechnology, China). The same amount of protein (40ng) was loaded for SDS-PAGE electrophoresis and then transferred to Millipore (Shanghai, China) and sealed in Tris buffer/Tween 20 (TBST) containing 5% skimmed milk powder for 2 hours at room temperature. Incubate primary antibody overnight at 4℃. The membrane was washed three times with TBST and then incubated at room temperature with conjugated HRP secondary antibodies for 2 hours. ECL (enhanced chemiluminescence kit, Santa Cruz Biotechnology, Dallas, TX) detection system (Thermo Scientific, Beijing, China) was used for detection. Scan with Chemi Imager 5500 V2.03. GAPDH was used as internal reference to determine the expression level of the target protein. Antibodies used are provided in Supplementary Table 3.

**Co-immunoprecipitation (Co-IP) assay**

Cells were lysed in RIPA (Beyotime Institute of Biotechnology) buffer on ice for 60 min and incubated with the antibody-coupled beads overnight at 4°C. The beads were washed three times with high-salt buffer, boiled for 10 min in SDS sample buffer, and then subjected to western blotting.

**Supplementary Table**

**Supplementary Table 1.** Primers

| **Gene** | **Sequence (5’ to 3’)** | **Use** |
| --- | --- | --- |
| **HUMAN** | | |
| *MAPK6P4* Fw | GATCGTGGTGAGCTAGAGCC | qPCR |
| *MAPK6P4* Rv | CAGTAAAGTGCCCTGCTCCA | qPCR |
| *KRT16P5* Fw | TCTCCTCCTCCTGCTTCTCC | qPCR |
| *KRT16P5* Rv | CTGCTGAAGCTACCGCCATA | qPCR |
| *TUBBP9* Fw | AGAGAACTGTGACTGCCTGC | qPCR |
| *TUBBP9* Rv | CCTGTAGGTTGGTGTGGTCC | qPCR |
| *VN1R10P* Fw | CATTGGGCTCATGGGGAACT | qPCR |
| *VN1R10P* Rv | GTTTGAGCCATGCCCACTTG | qPCR |
| *BET1P1* Fw | GCCCACCTTTTCCCTCTCTC | qPCR |
| *BET1P1* Rv | CACAGCAGCTTTGTTTGGCT | qPCR |
| *KRT16P2* Fw | TTTTTCAGCCAACTGCTCGC | qPCR |
| *KRT16P2* Rv | CTCTGTGGTTGTGCTTTGCC | qPCR |
| *CRB3P1* Fw | ACCCTGGGCGCAAATATCAA | qPCR |
| *CRB3P1* Rv | CCACTGCCAGGAACAAGACA | qPCR |
| *CLUHP6* Fw | AGCAGAAACAGGGATGAGGC | qPCR |
| *CLUHP6* Rv | ATGGAAGAAGAGGGCCTTGC | qPCR |
| *EEF1GP5* Fw | ATGCGTCACAACAAACAGGC | qPCR |
| *EEF1GP5* Rv | GACAGTGCAGAACTGAGGCT | qPCR |
| *NUTM2HP* Fw | GGAGGGAGGGAAGACTGGAT | qPCR |
| *NUTM2HP* Rv | AGAGGACAGGCCATGAGCTA | qPCR |
| *MAPK6* Fw | CAGGCTTTTCATGTATCAGCTG | qPCR |
| *MAPK6* Rv | AGCACCAAGTCTTCCGTATTAA | qPCR |
| *KLF15* Fw | GACAGCATCTTGGACTTCCTAT | qPCR |
| *KLF15* Rv | CATCAGGATCACCCAAAGGAAA | qPCR |
| *LDHA* Fw | AGGTGATCAAACTCAAAGGCTA | qPCR |
| *LDHA* Rv | CCCAAAATGCAAGGAACACTAA | qPCR |
| *DSP* Fw | GGCTACCGGGCTCAGATAGA | qPCR |
| *DSP* Rv | AACCGCGTGATATCCTGGTC | qPCR |
| *UNC5C* Fw | TTGCATGCAGACTGCTCCTG | qPCR |
| *UNC5C* Rv | CCCATTGAGTGCCGAAGAGT | qPCR |
| *DACH1* Fw | TCAAGTGTCGGACTGGAACT | qPCR |
| *DACH1* Rv | GGGGGACTCTGAACTTGTGC | qPCR |
| *EDNRB* Fw | GCTTTTGCAAACCGCAGAGA | qPCR |
| *EDNRB* Rv | CAGAACCACAGAGACCACCC | qPCR |
| *KDR* Fw | CGGTCAACAAAGTCGGGAGA | qPCR |
| *KDR* Rv | CAGTGCACCACAAAGACACG | qPCR |
| *CDH5* Fw | TCACGATAACACGGCCAACA | qPCR |
| *CDH5* Rv | CGTCAGAGTCGGATGAGTCG | qPCR |
| *GAPDH* Fw | GGACGTGCAGGGCAACTACC | qPCR |
| *GAPDH* Rv | AGCCGACGATGAGAAAGGGG | qPCR |
| *LDHA* Fw1 | ACATTTGGTAGGCAGGCAGG | ChIP |
| *LDHA* Rv1 | GGGCTCTCACCTCAAACACA | ChIP |
| *LDHA* Fw2 | CCAGCCCTGACTGAGAACAC | ChIP |
| *LDHA* Rv2 | GGGCTCTCACCTCAAACACA | ChIP |

**Supplementary Table 2.** shRNA used for transfection

| **Gene** | **Target Sequence (5’ to 3’)** |
| --- | --- |
| **HUMAN** | |
| MAPK6P4 shRNA1 | GCCAGCACCTTAGAACAATGC |
| MAPK6P4 shRNA2 | GCTCAGGACTAGTCCAACACT |
| KLF15 shRNA1 | CTACCCTGGAGGAGATTGAAG |
| KLF15 shRNA2 | CAGTTGGGTATCTGGGGTGATA |
| LDHA shRNA1 | CCAAAGATTGTCTCTGGCAAA |
| LDHA shRNA2 | CCACCATGATTAAGGGTCTTT |

**Supplementary Table 3.** plasmid sequences

| Gene | plasmid sequences |
| --- | --- |
| MAPK6P4-ORF | ATGGAGACAGACCTAGCTAATGTGCTGGAGCAGGGCACTTTACTGGAAGAGCATGCCAGGCTTTTCATGTATCAGCTGCTACGGGGGGTCAAGAATATTCACTCTGCAAATGTACTGCACAGAGATCTCAAACCAGTGAATCTTTTCATTAATACTGAAGACTTGGTGCTGAAGATATGTGACTTTGGTCTTGCACGGATTACGAATACTCATTATTCCCATAAGGGTCATCTTTCTGAAGGATTGGTTACTAAATGGTACAGATCTCCACGTCTTTTACTTTCTCCTAACAATTATACTAAAGCCATTGACATGTGGGCTGCAGGCTGCATCTTTGCTGAAATGCTGACTGGTAAAATCCTTTTTGCAGGTGCACATGAACTTGAACAGATGCAACTGATTTTATAA |
| MAPK6P4-Mut | ATTGAGACAGACCTAGCTAATGTGCTGGAGCAGGGCACTTTACTGGAAGAGCATGCCAGGCTTTTCATGTATCAGCTGCTACGGGGGGTCAAGAATATTCACTCTGCAAATGTACTGCACAGAGATCTCAAACCAGTGAATCTTTTCATTAATACTGAAGACTTGGTGCTGAAGATATGTGACTTTGGTCTTGCACGGATTACGAATACTCATTATTCCCATAAGGGTCATCTTTCTGAAGGATTGGTTACTAAATGGTACAGATCTCCACGTCTTTTACTTTCTCCTAACAATTATACTAAAGCCATTGACATGTGGGCTGCAGGCTGCATCTTTGCTGAAATGCTGACTGGTAAAATCCTTTTTGCAGGTGCACATGAACTTGAACAGATGCAACTGATTTTATAA |
| KLF15-WT | CCACGCCTGATGGCCCCATCCCAGTGTTGCTGCAGATCCAGCCCGTGCCTGTGAAGCAGGAATCGGGCACAGGGCCTGCCTCCCCTGGGCAAGCCCCAGAGAATGTCAAGGTTGCCCAGCTCCTGGTCAACATCCAGGGGCAGACCTTCGCACTCGTGCCCCAGGTGGTACCCTCCTCCAACTTGAACCTGCCCTCCAAGTTTGTGCGC |
| KLF15-S238A | CCACGCCTGATGGCCCCATCCCAGTGTTGCTGCAGATCCAGCCCGTGCCTGTGAAGCAGGAATCGGGCACAGGGCCTGCCGCCCCTGGGCAAGCCCCAGAGAATGTCAAGGTTGCCCAGCTCCTGGTCAACATCCAGGGGCAGACCTTCGCACTCGTGCCCCAGGTGGTACCCTCCTCCAACTTGAACCTGCCCTCCAAGTTTGTGCGC |
| KLF15-S238D | CCACGCCTGATGGCCCCATCCCAGTGTTGCTGCAGATCCAGCCCGTGCCTGTGAAGCAGGAATCGGGCACAGGGCCTGCCGACCCTGGGCAAGCCCCAGAGAATGTCAAGGTTGCCCAGCTCCTGGTCAACATCCAGGGGCAGACCTTCGCACTCGTGCCCCAGGTGGTACCCTCCTCCAACTTGAACCTGCCCTCCAAGTTTGTGCGC |

**Supplementary Table 4.** Antibodies

| **Protein** | **Antibody** | **Origin** | **Dilution** |
| --- | --- | --- | --- |
| MAPK6 | sc-365234, Santa | Mouse | 1:500 for WB |
| P4-135aa | 135-Pep-KLH, Beijing Protein Invovation | Rabbit | 1:500 for WB; 1:50 for IF |
| KLF15 | ab167192, Abcam | Mouse | 1:500 for WB; 1:50 for IF |
| LDHA | 66287-1-Ig, Proteintech | Mouse | 1:500 for WB; 1:50 for IF |
| VE-cadherin | 66804-1-Ig, Proteintech | Mouse | 1:500 for WB; 1:50 for IF |
| VEGFR2 | 26415-1-AP, Proteintech | Rabbit | 1:500 for WB; 1:50 for IF |
| GAPDH | 60004-1-lg, Proteintech | Mouse | 1:10000 for WB |
| Histone H3 | 17168-1-AP, Proteintech | Rabbit | 1:10000 for WB |
| FLAG | 80010-1-RR, Proteintech | Rabbit | 1:2000 |
| GFP | 50430-2-AP | Rabbit | 1:2000 |
| Phosphoserine (p-Ser) | ab9332, Abcam | Rabbit | 1:500 |
| Ubiquitin | sc-8017, Santa Cruz Biotechnolog | Mouse | 1:1000 |
| Kla | PTM-1401RM, PTM Biolabs | Rabbit | 1:500 |
| IgG | ab18413, Abcam | Mouse | 1:10 |
| CD34 | 14486-1-AP Proteintech | Rabbit | 1:50 for IHC |
| Goat anti-mouse | SA00001-1, Proteintech |  | 1:10000 |
| Goat anti-rabbit | SA00001-2, Proteintech |  | 1:10000 |
| Donkey anti-Goat | SA00001-3, Proteintech |  | 1:10000 |
| Alexa-Fluor-488-labeled Goat anti-Mouse IgG (H+L) | A0428, Proteintech | Goat | 1:500 |
| Alexa-Fluor-488-labeled Goat anti-Rabbit IgG (H+L) | A0423, Proteintech | Goat | 1:500 |
| Alexa-Fluor-555-labeled Donkey anti-Mouse IgG (H+L) | A0460, Proteintech | Donkey | 1:500 |
| Alexa-Fluor-555-labeled Donkey anti-Rabbit IgG (H+L) | A0453, Proteintech | Donkey | 1:500 |
