# Supplementary material for: Pseudogene MAPK6P4-encoded functional peptide promotes glioblastoma vasculogenic mimicry development
Source: Commun Biol. 2023 Oct 18;6:1059. doi: 10.1038/s42003-023-05438-1 (PMC10584926; doi:10.1038/s42003-023-05438-1)
Supplement: Supplementary file 4 — Reporting Summary [file 42003_2023_5438_MOESM4_ESM.pdf]

nature portfolio

Corresponding author(s): Yixue Xue

Last updated by author(s): Jul 3, 2023

# Reporting Summary

Nature Portfolio wishes to improve the reproducibility of the work that we publish. This form provides structure for consistency and transparency in reporting. For further information on Nature Portfolio policies, see our Editorial Policies and the Editorial Policy Checklist.

## Statistics

For all statistical analyses, confirm that the following items are present in the figure legend, table legend, main text, or Methods section.

| n/a | Confirmed | |
|---|---|---|
| ☐ | ☒ | The exact sample size (*n*) for each experimental group/condition, given as a discrete number and unit of measurement |
| ☒ | ☐ | A statement on whether measurements were taken from distinct samples or whether the same sample was measured repeatedly |
| ☐ | ☒ | The statistical test(s) used AND whether they are one- or two-sided<br>*Only common tests should be described solely by name; describe more complex techniques in the Methods section.* |
| ☒ | ☐ | A description of all covariates tested |
| ☒ | ☐ | A description of any assumptions or corrections, such as tests of normality and adjustment for multiple comparisons |
| ☐ | ☒ | A full description of the statistical parameters including central tendency (e.g. means) or other basic estimates (e.g. regression coefficient) AND variation (e.g. standard deviation) or associated estimates of uncertainty (e.g. confidence intervals) |
| ☐ | ☒ | For null hypothesis testing, the test statistic (e.g. *F*, *t*, *r*) with confidence intervals, effect sizes, degrees of freedom and *P* value noted<br>*Give P values as exact values whenever suitable.* |
| ☒ | ☐ | For Bayesian analysis, information on the choice of priors and Markov chain Monte Carlo settings |
| ☒ | ☐ | For hierarchical and complex designs, identification of the appropriate level for tests and full reporting of outcomes |
| ☒ | ☐ | Estimates of effect sizes (e.g. Cohen's *d*, Pearson's *r*), indicating how they were calculated |

*Our web collection on statistics for biologists contains articles on many of the points above.*

## Software and code

Policy information about availability of computer code

| | |
|---|---|
| Data collection | No software was used. |
| Data analysis | No software was used. |

For manuscripts utilizing custom algorithms or software that are central to the research but not yet described in published literature, software must be made available to editors and reviewers. We strongly encourage code deposition in a community repository (e.g. GitHub). See the Nature Portfolio guidelines for submitting code & software for further information.

## Data

Policy information about availability of data

All manuscripts must include a data availability statement. This statement should provide the following information, where applicable:

- Accession codes, unique identifiers, or web links for publicly available datasets
- A description of any restrictions on data availability
- For clinical datasets or third party data, please ensure that the statement adheres to our policy

Data supporting the present study are available from the corresponding author upon reasonable request.

## Human research participants

Policy information about studies involving human research participants and Sex and Gender in Research.

| | |
|---|---|
| Reporting on sex and gender | N/A |
| Population characteristics | N/A |
| Recruitment | N/A |
| Ethics oversight | N/A |

Note that full information on the approval of the study protocol must also be provided in the manuscript.

# Field-specific reporting

Please select the one below that is the best fit for your research. If you are not sure, read the appropriate sections before making your selection.

☒ Life sciences ☐ Behavioural & social sciences ☐ Ecological, evolutionary & environmental sciences

For a reference copy of the document with all sections, see nature.com/documents/nr-reporting-summary-flat.pdf

# Life sciences study design

All studies must disclose on these points even when the disclosure is negative.

| | |
|---|---|
| Sample size | All experiments were repeated at least three times independently. Statistical analysis of the data was performed using Student's t-test and one-way ANOVA in GraphPad Prism v5.01. Data are presented as mean ± SD. P < 0.05 was considered significant. |
| Data exclusions | No data were excluded from the analyses. |
| Replication | Findings were reliably reproduced according to the methods. |
| Randomization | Samples were allocated into experimental groups randomly. |
| Blinding | N/A |

# Reporting for specific materials, systems and methods

We require information from authors about some types of materials, experimental systems and methods used in many studies. Here, indicate whether each material, system or method listed is relevant to your study. If you are not sure if a list item applies to your research, read the appropriate section before selecting a response.

## Materials & experimental systems

| n/a | Involved in the study | |
|---|---|---|
| ☐ | ☒ | Antibodies |
| ☐ | ☒ | Eukaryotic cell lines |
| ☒ | ☐ | Palaeontology and archaeology |
| ☐ | ☒ | Animals and other organisms |
| ☒ | ☐ | Clinical data |
| ☒ | ☐ | Dual use research of concern |

## Methods

| n/a | Involved in the study | |
|---|---|---|
| ☒ | ☐ | ChIP-seq |
| ☒ | ☐ | Flow cytometry |
| ☒ | ☐ | MRI-based neuroimaging |

## Antibodies

Antibodies used

MAPK6 sc-365234, Santa
P4-135aa 135-Pep-KLH, Beijing Protein Invovation
KLF15 ab167192, Abcam
LDHA 66287-1-Ig, Proteintech
VE-cadherin 66804-1-Ig, Proteintech
VEGFR2 26415-1-AP, Proteintech
GAPDH 60004-1-Ig, Proteintech

| | |
|---|---|
| | Histone H3 17168-1-AP, Proteintech<br>Phosphoserine (p-Ser) ab9332, Abcam<br>Ubiquitin sc-8017, Santa Cruz Biotechnolog<br>Kla PTM-1401RM, PTM Biolabs<br>IgG ab18413, Abcam<br>CD34 14486-1-AP Proteintech<br>Goat anti-mouse SA00001-1, Proteintech<br>Goat anti-rabbit SA00001-2, Proteintech |
| Validation | All antibodys were KD/KO validated. |

## Eukaryotic cell lines

Policy information about cell lines and Sex and Gender in Research

| | |
|---|---|
| Cell line source(s) | The human GBM cell lines (U251, U373) and 293T cells were purchased from the Shanghai Institutes for Biological Sciences Cell Resource Center. |
| Authentication | All cell lines have STR authentication. |
| Mycoplasma contamination | All cell lines tested negative for mycoplasma contamination. |
| Commonly misidentified lines<br>(See ICLAC register) | N/A |

## Animals and other research organisms

Policy information about studies involving animals; ARRIVE guidelines recommended for reporting animal research, and Sex and Gender in Research

| | |
|---|---|
| Laboratory animals | Female 4-week-old BALB/c nude mice. |
| Wild animals | N/A |
| Reporting on sex | N/A |
| Field-collected samples | N/A |
| Ethics oversight | All experiments were carried out strictly following the Animal Welfare Act and approved by the Ethics Committee of China Medical University. |

Note that full information on the approval of the study protocol must also be provided in the manuscript.
