# Supplementary material for: Pseudogene MAPK6P4-encoded functional peptide promotes glioblastoma vasculogenic mimicry development
Source: Commun Biol. 2023 Oct 18;6:1059. doi: 10.1038/s42003-023-05438-1 (PMC10584926; doi:10.1038/s42003-023-05438-1)
Supplement: Supplementary file 5 — Uncropped original western blots [file 42003_2023_5438_MOESM5_ESM.docx]

**Uncropped original western blots**

Figure 1e

U251


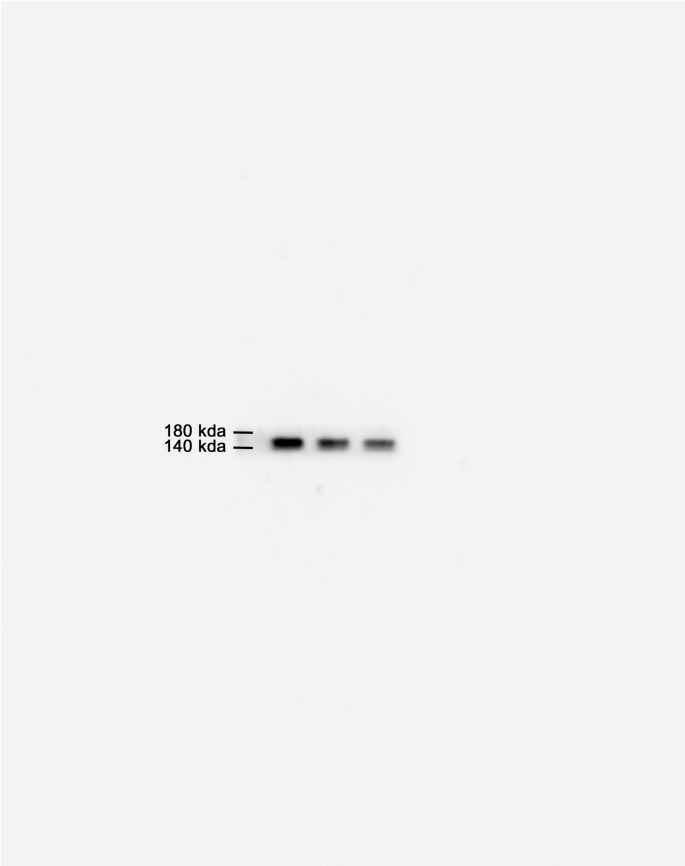
VEGFR2


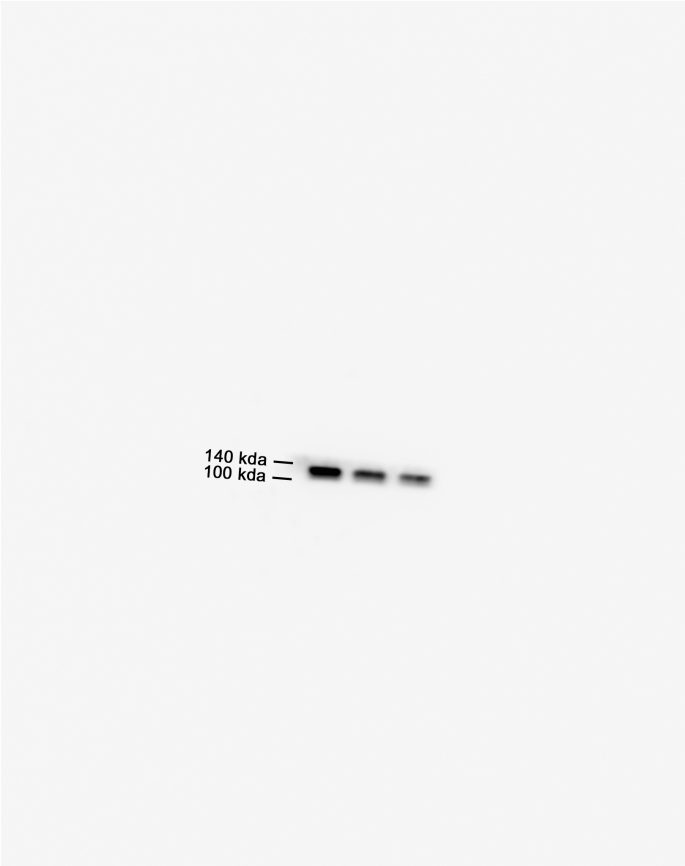
VE-cadherin


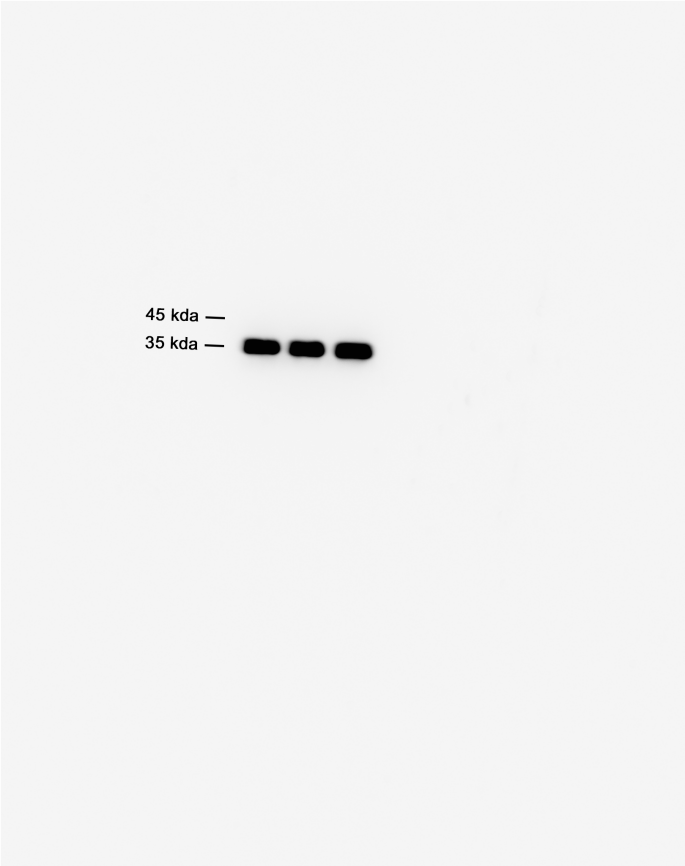
GAPDH

U373


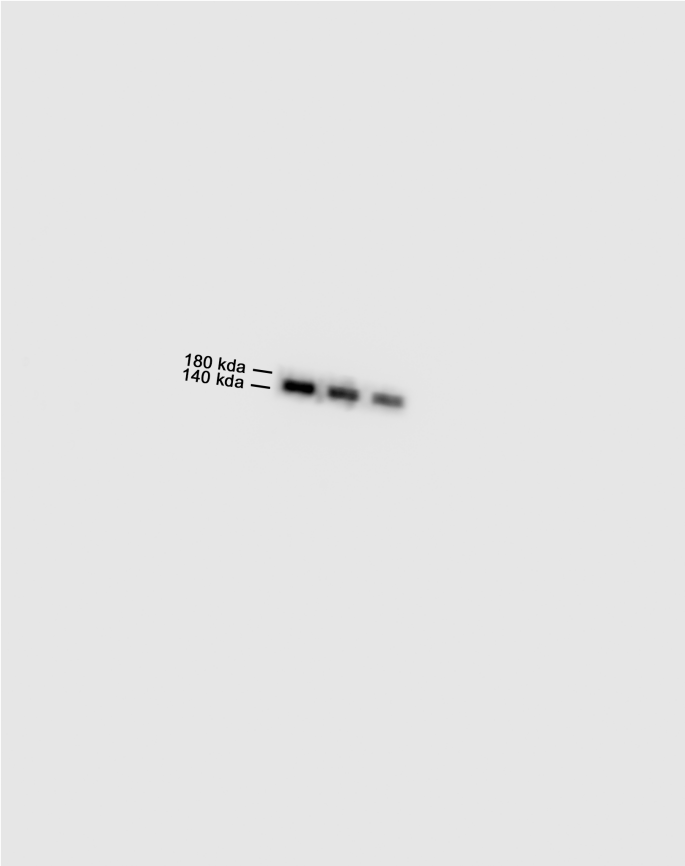
VEGFR2


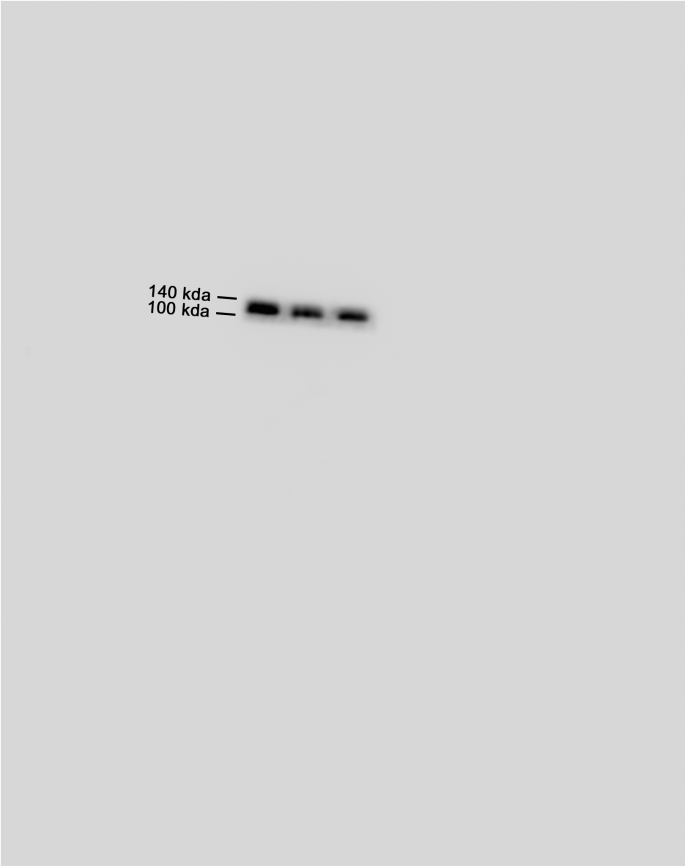
VE-cadherin


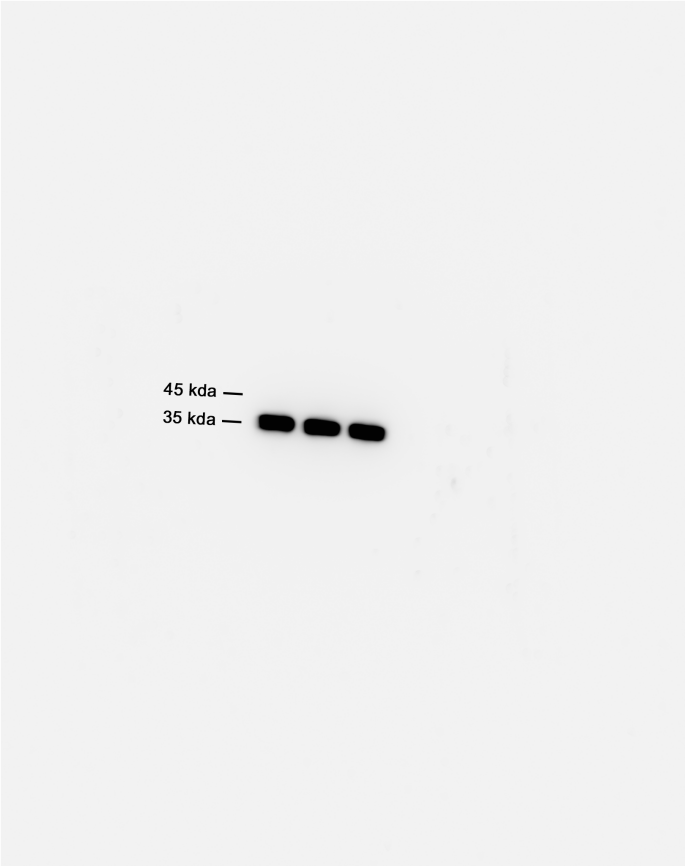
GAPDH

Figure 1e

U251


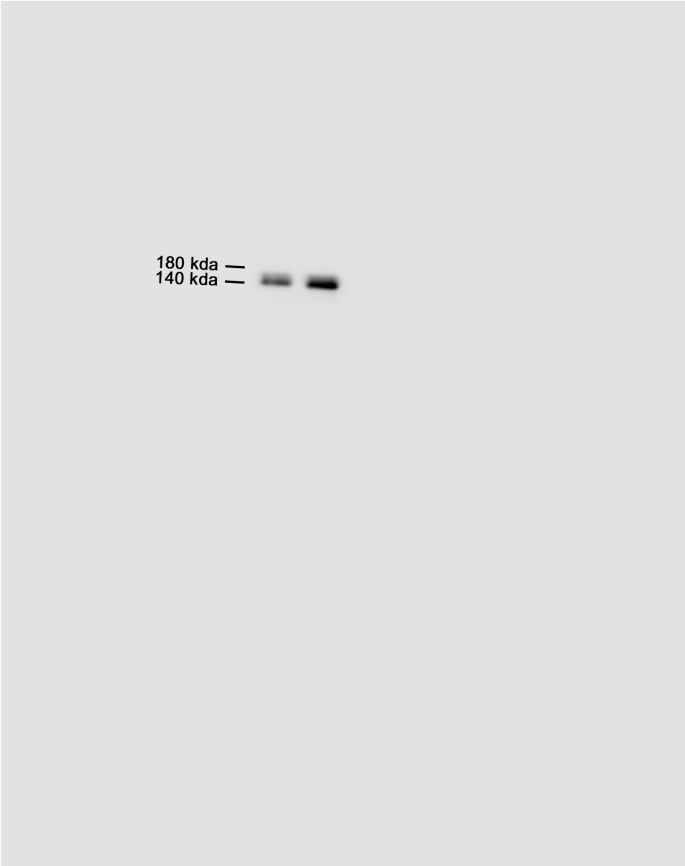
VEGFR2


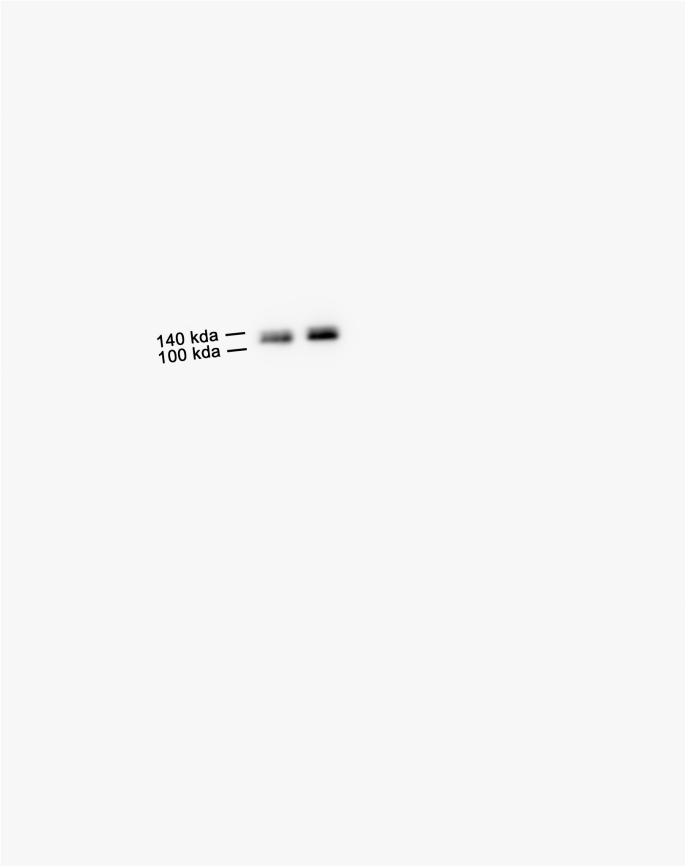
VE-cadherin


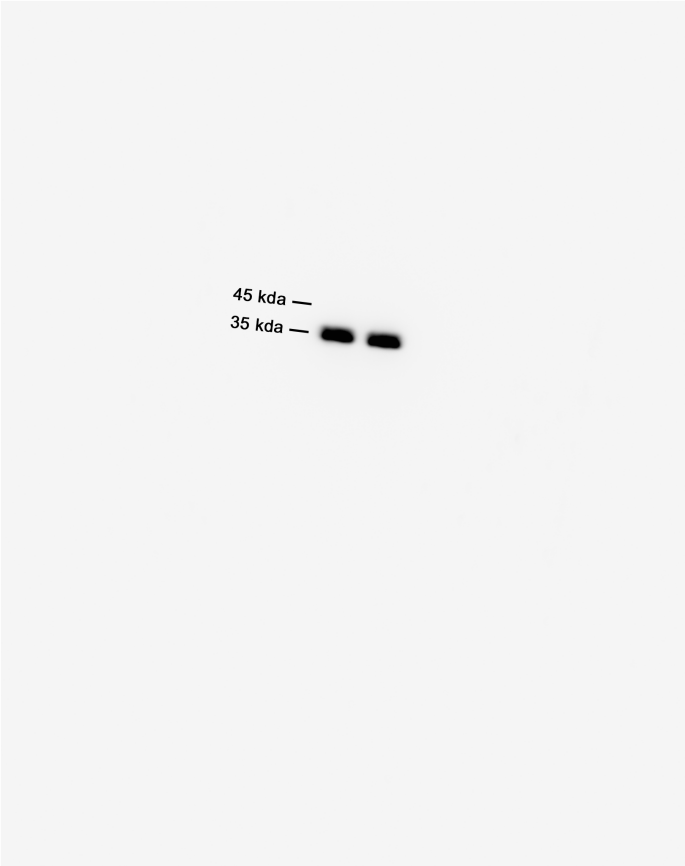
GAPDH

U373


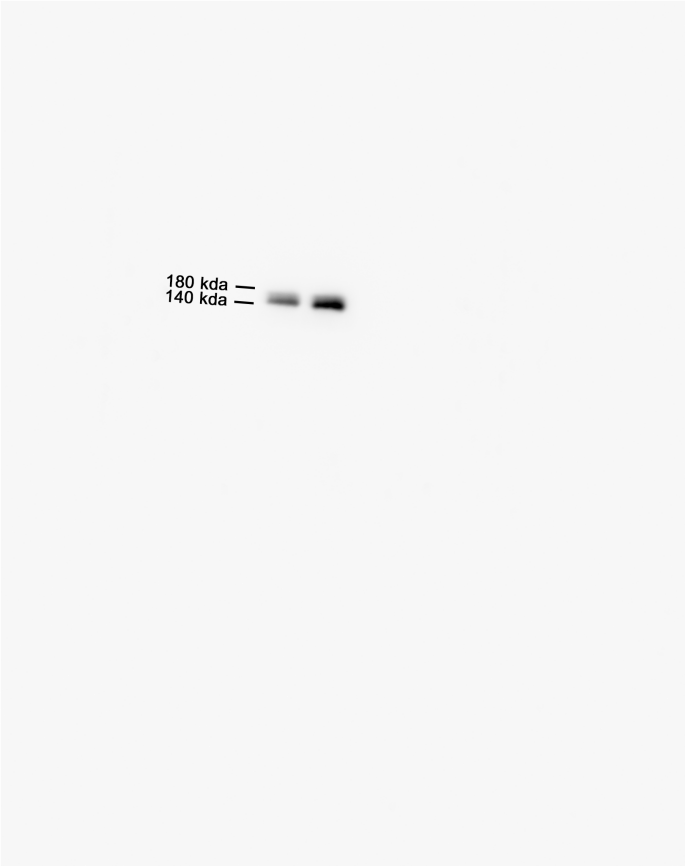
VEGFR2


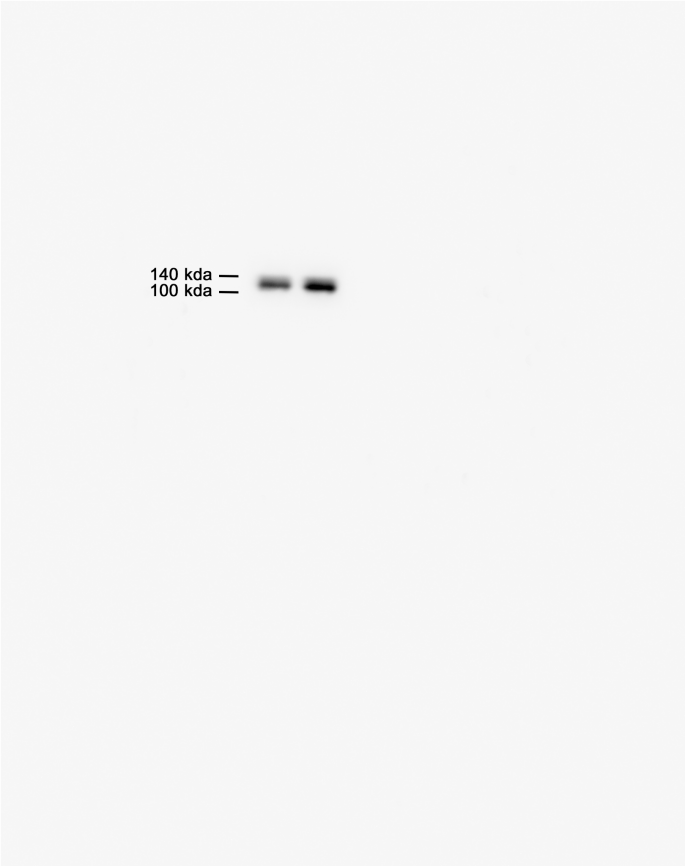
VE-cadherin


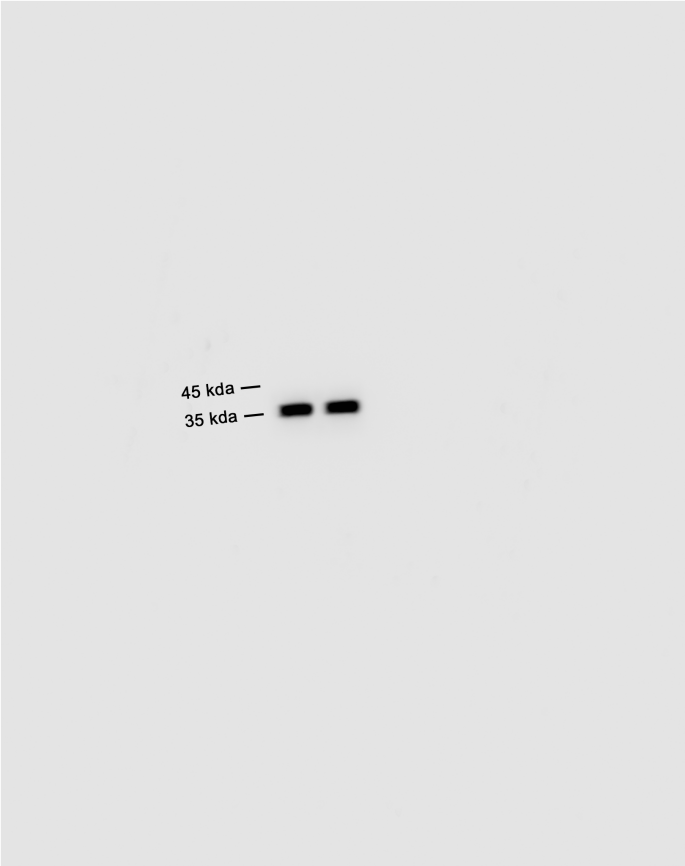
GAPDH

Figure 2c


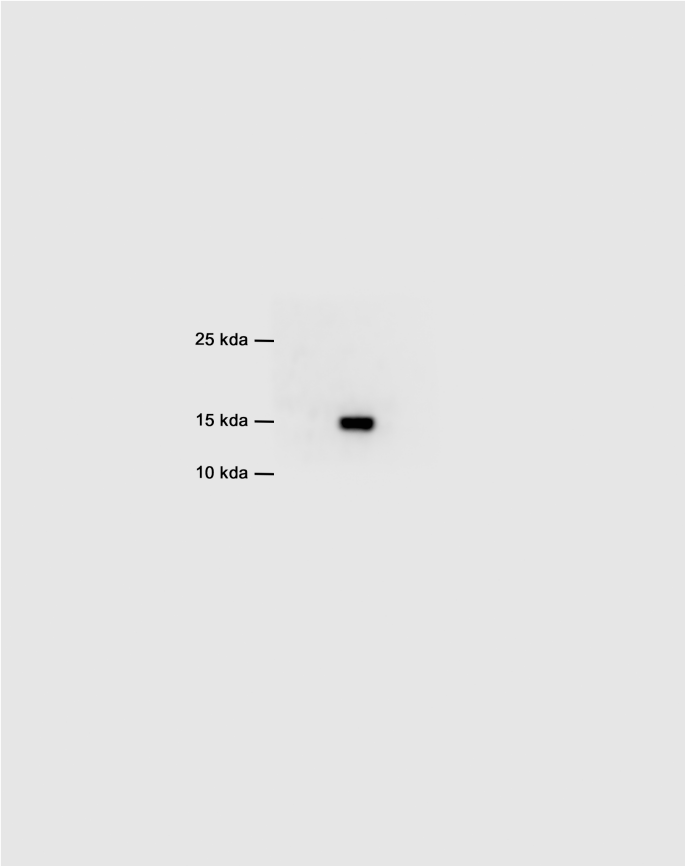
FLAG


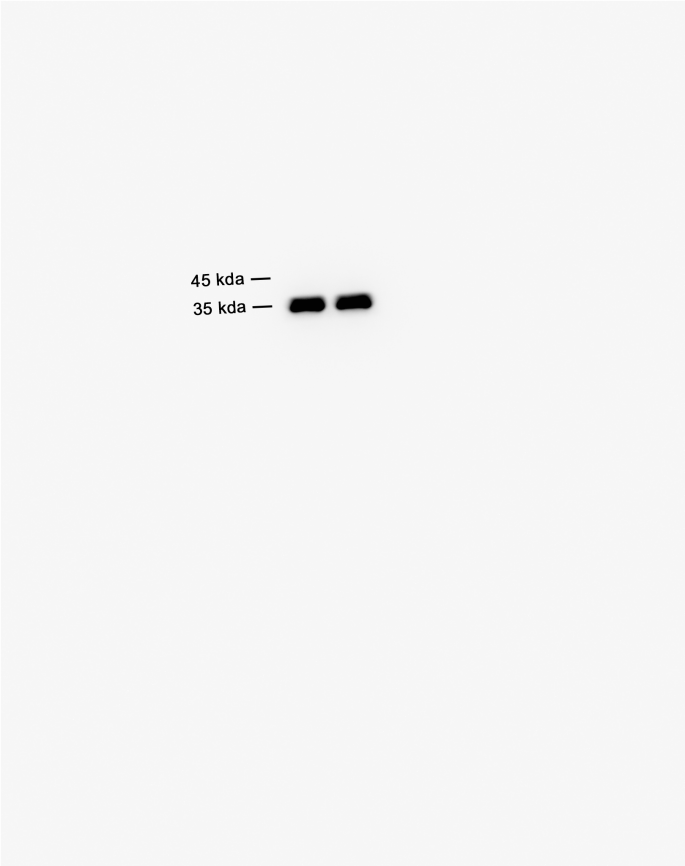
GAPDH

Figure 2d

U251


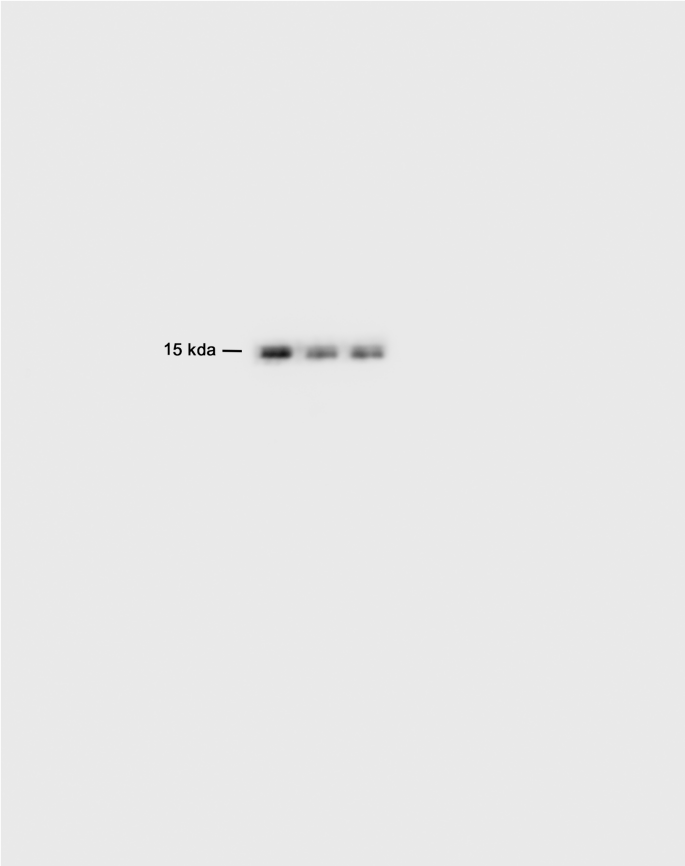
P4-135aa


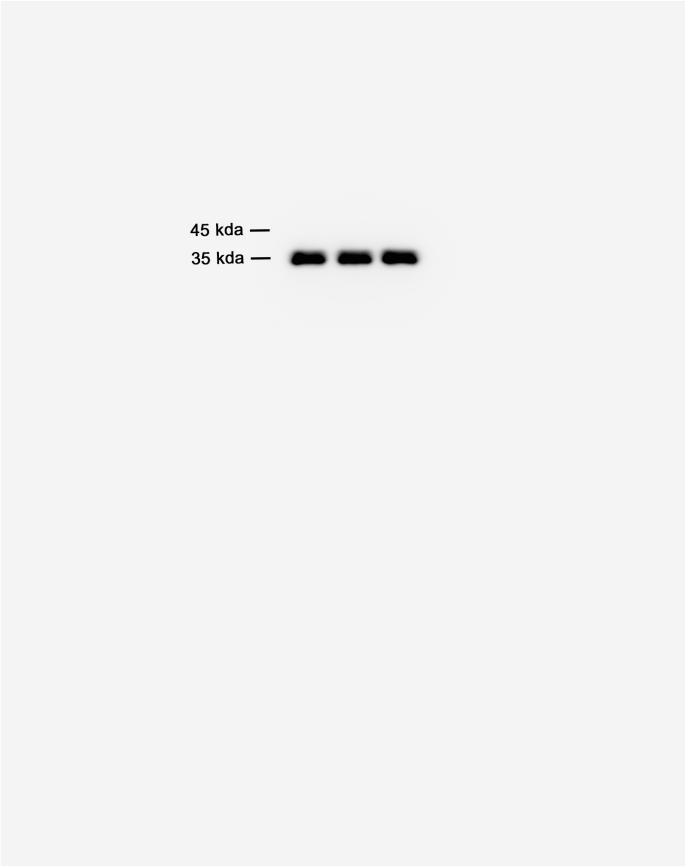
GAPDH

U373


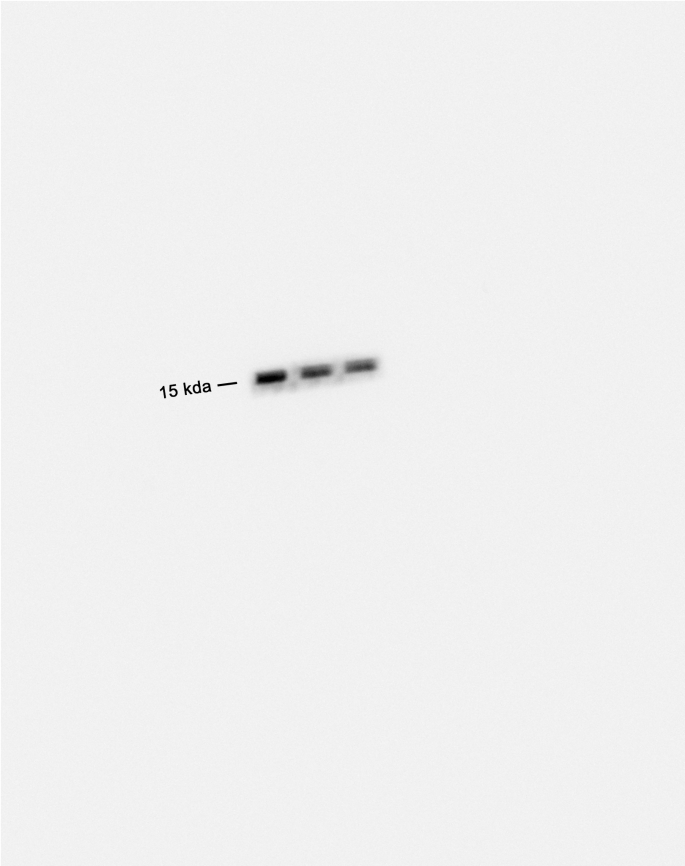
P4-135aa


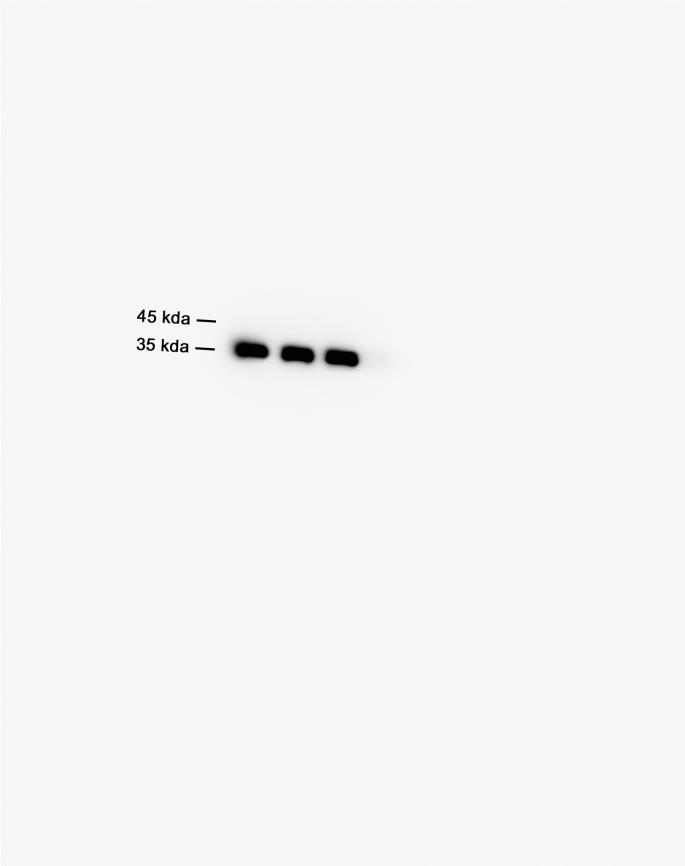
GAPDH

Figure 2e

U251


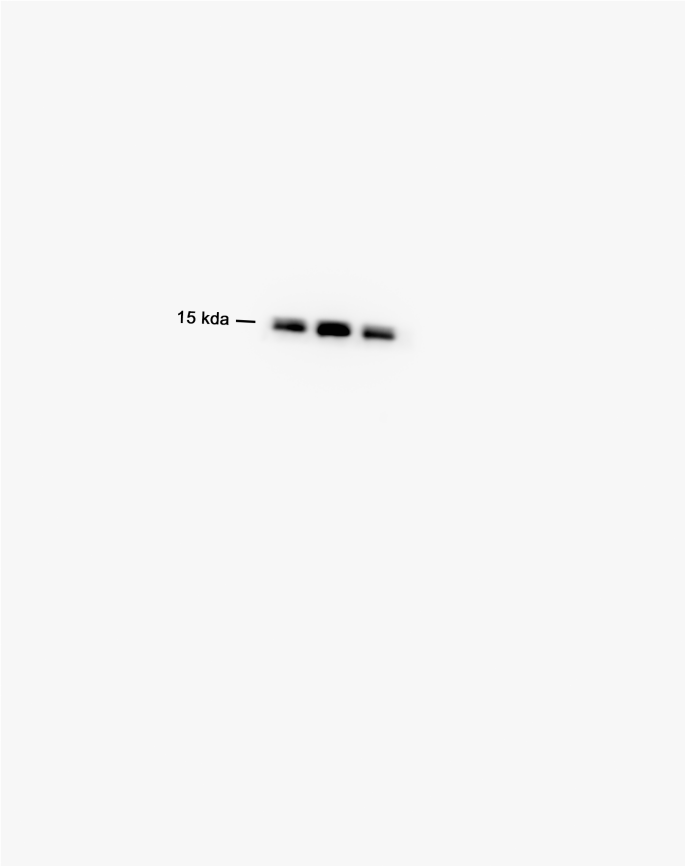
P4-135aa


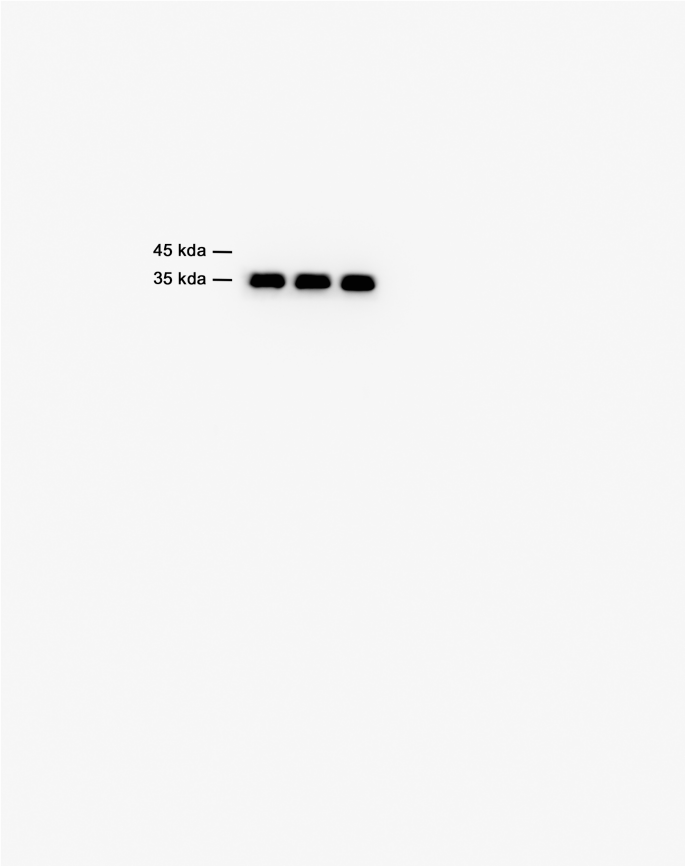
GAPDH

U373


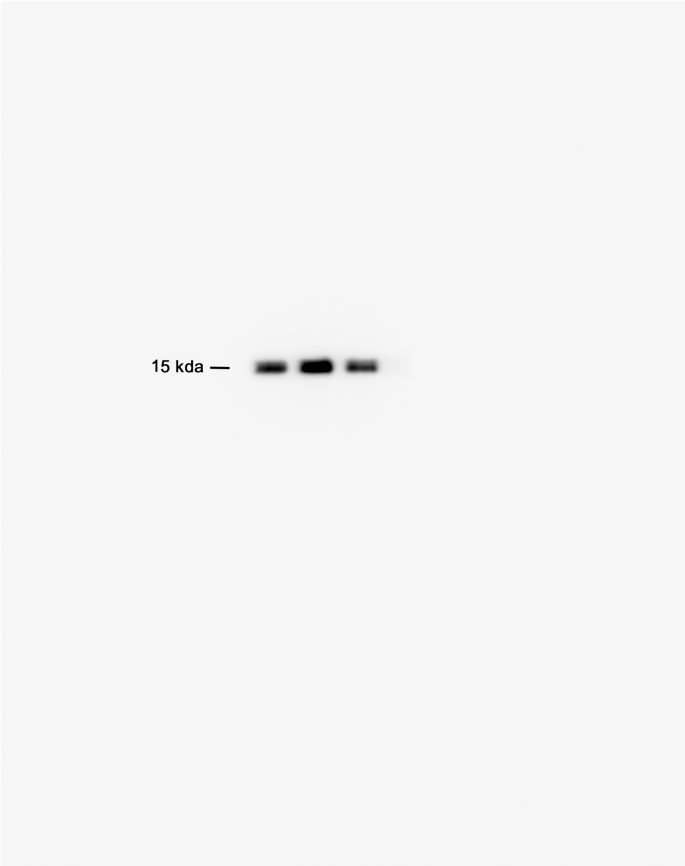
P4-135aa


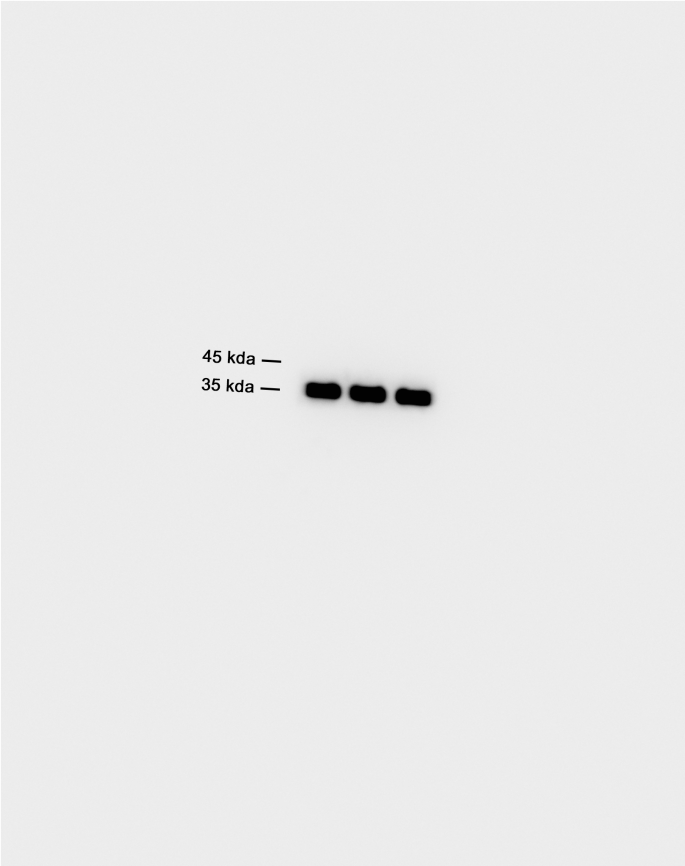
GAPDH

Figure 2f


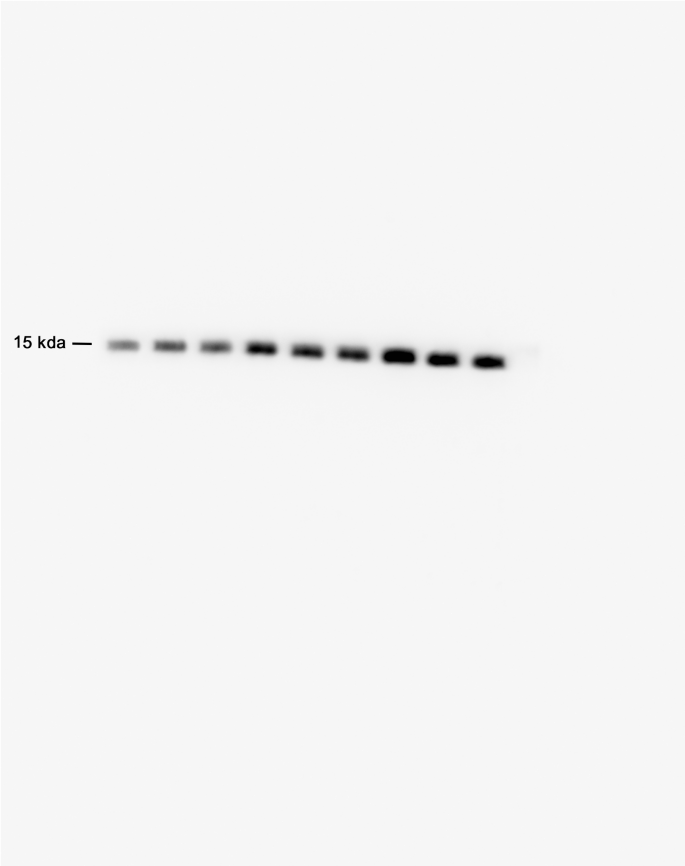
P4-135aa


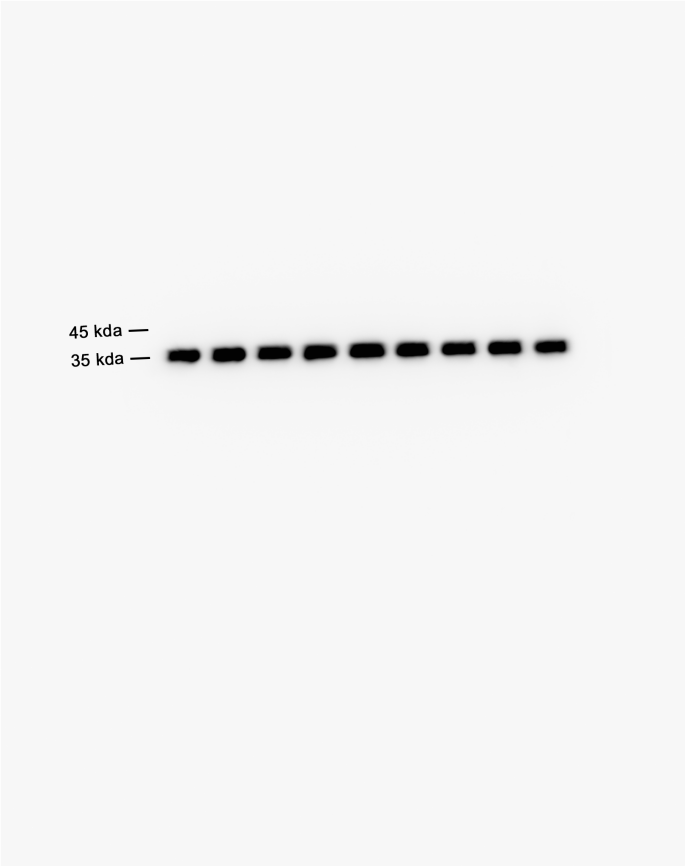
GAPDH


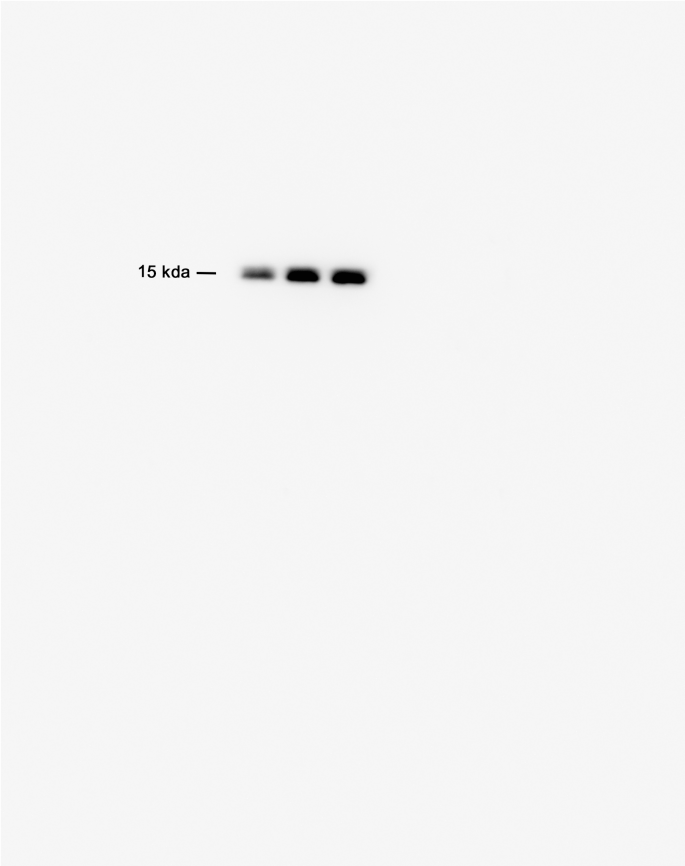
P4-135aa


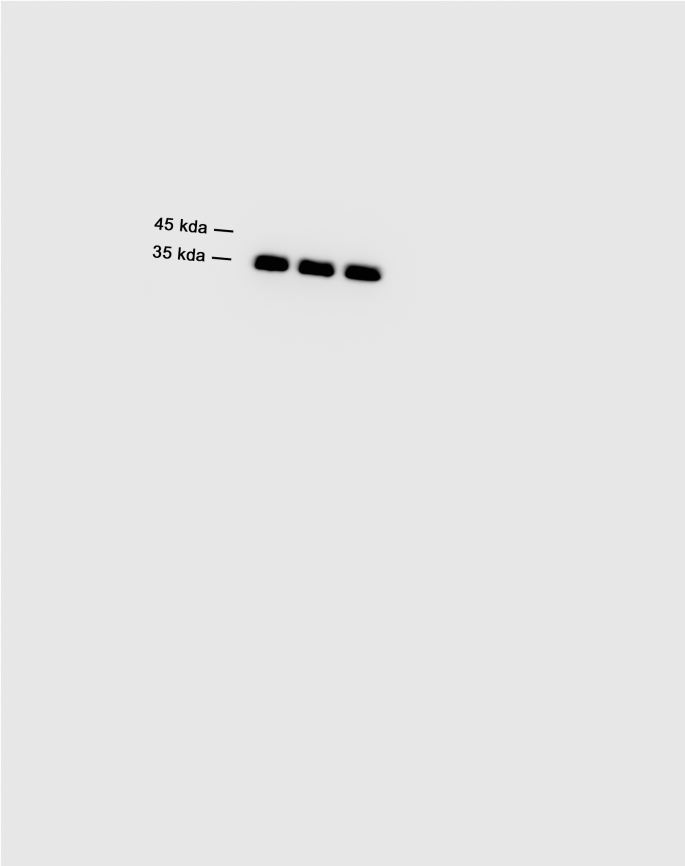
GAPDH

Figure 2g

U251


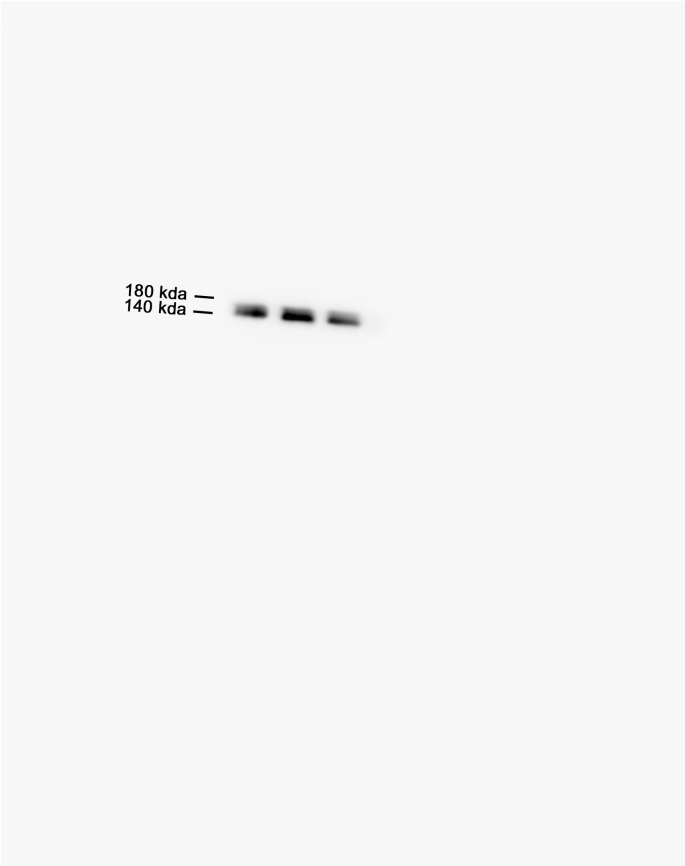
VEGFR2


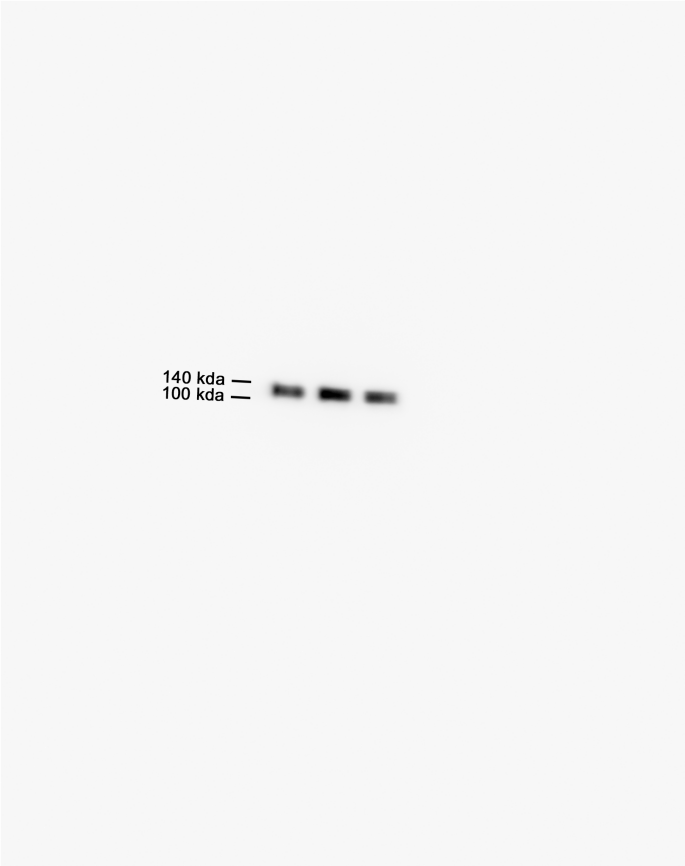
VE-cadherin


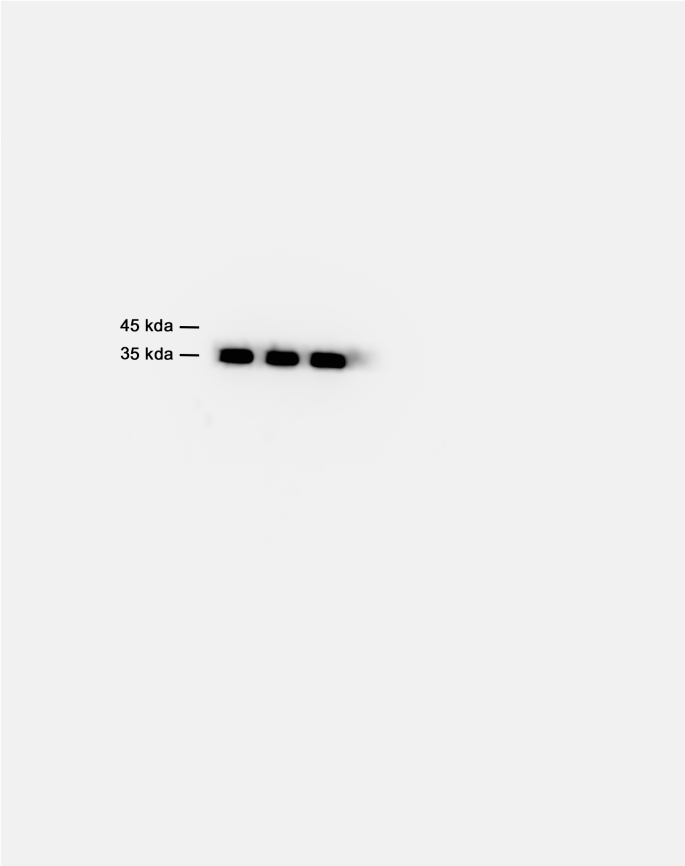
GAPDH

U373


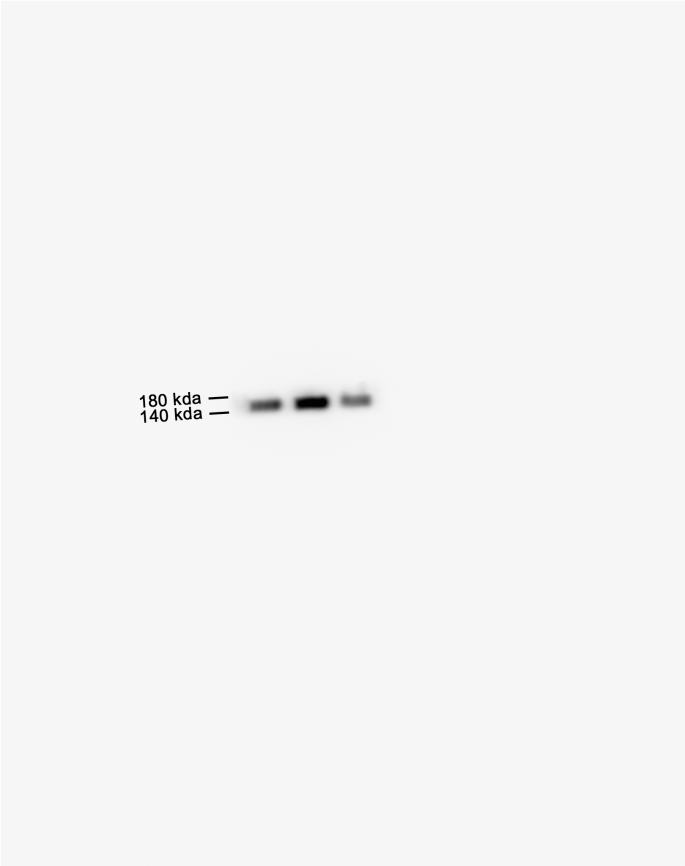
VEGFR2


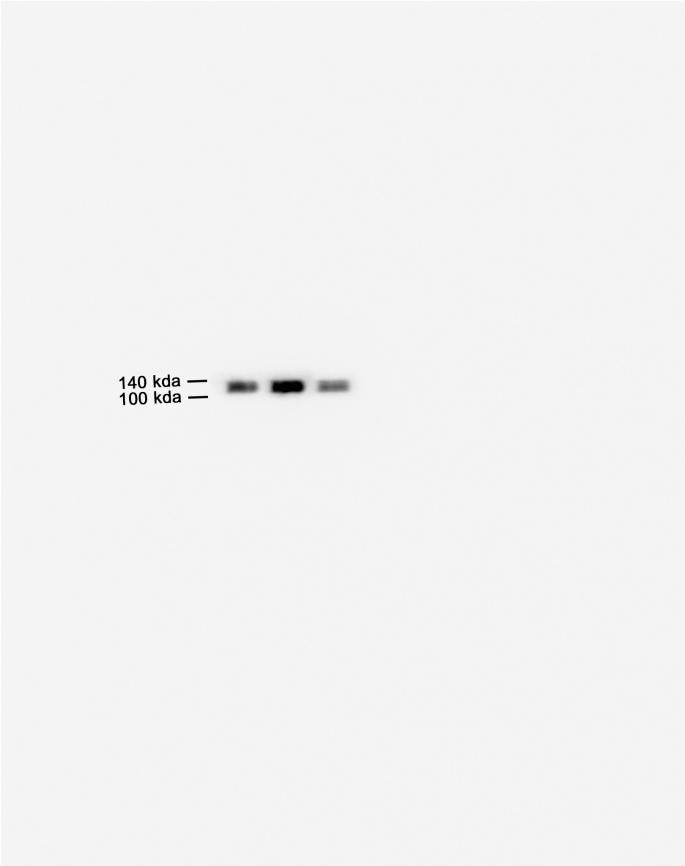
VE-cadherin


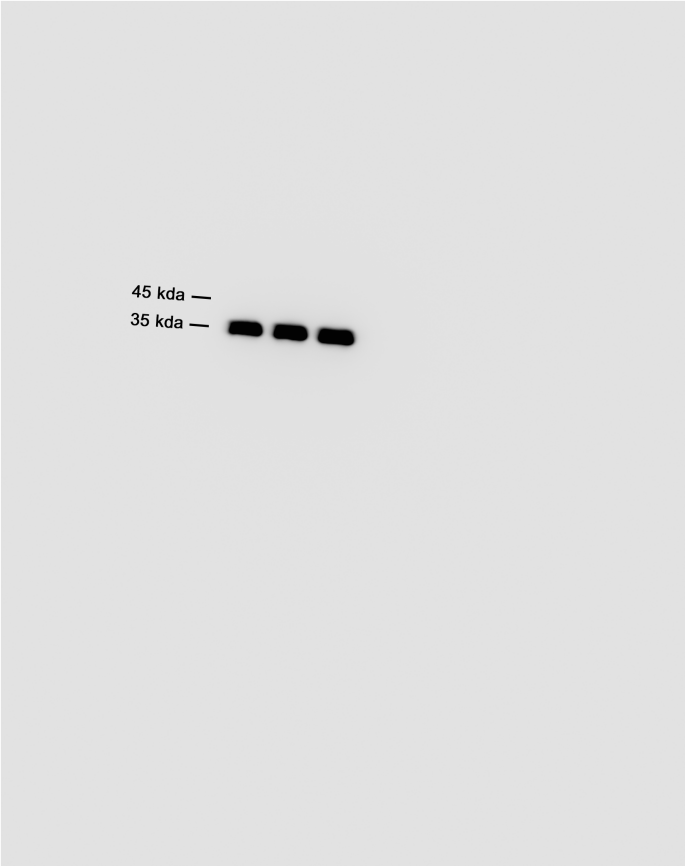
GAPDH

Figure 3c

U251


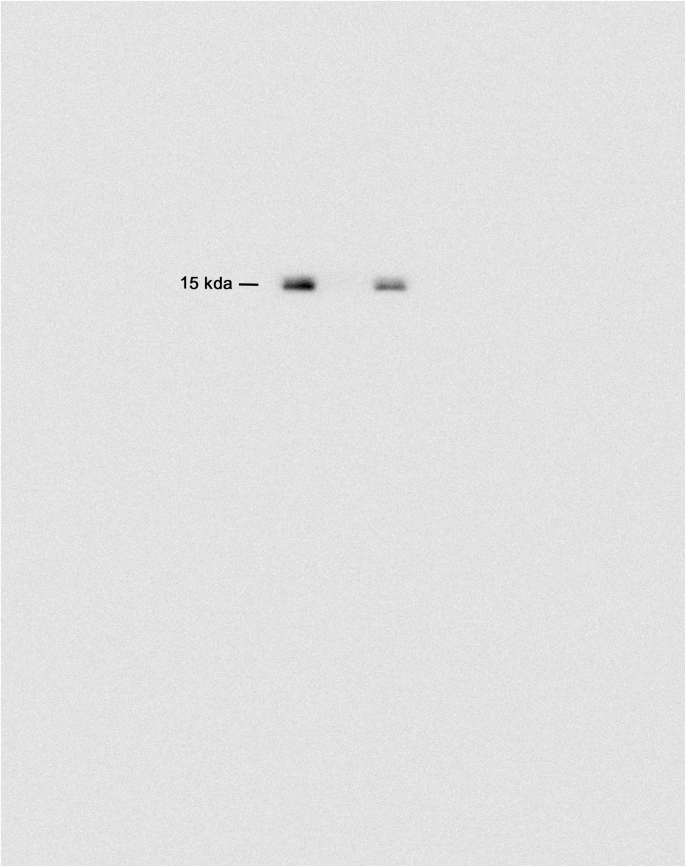
P4-135aa


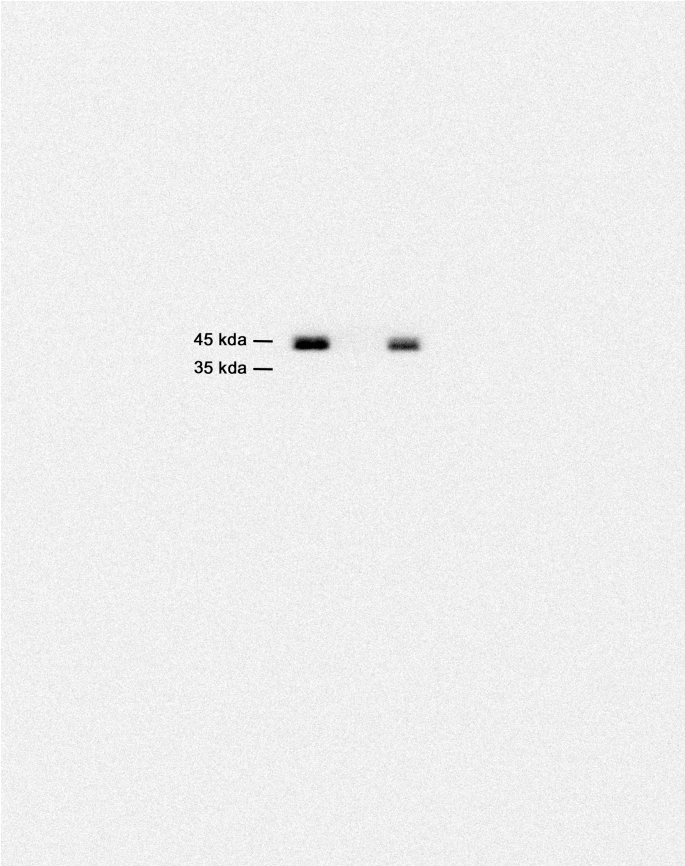
KLF15

U373


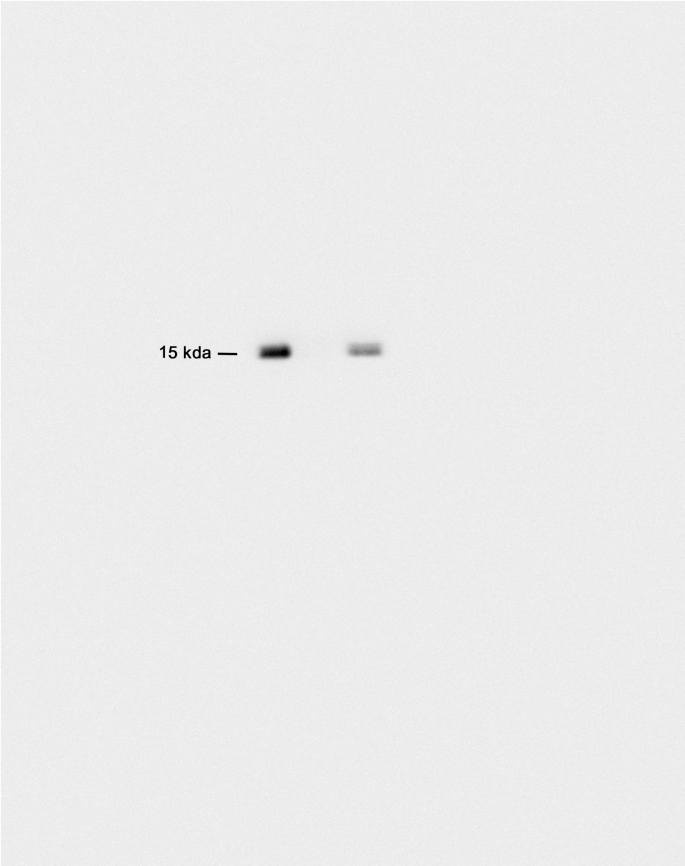
P4-135aa


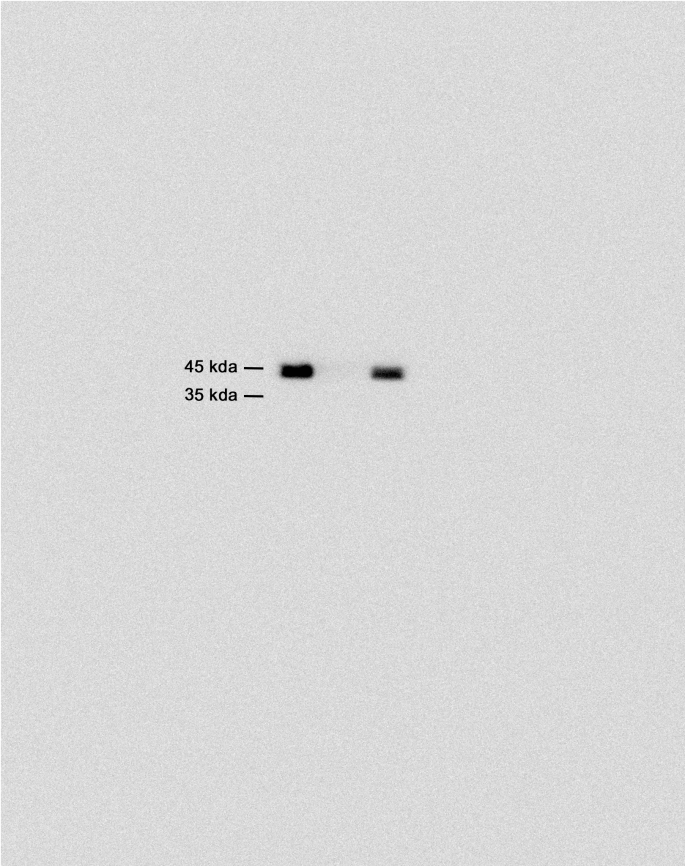
KLF15

Figure 3e

U251


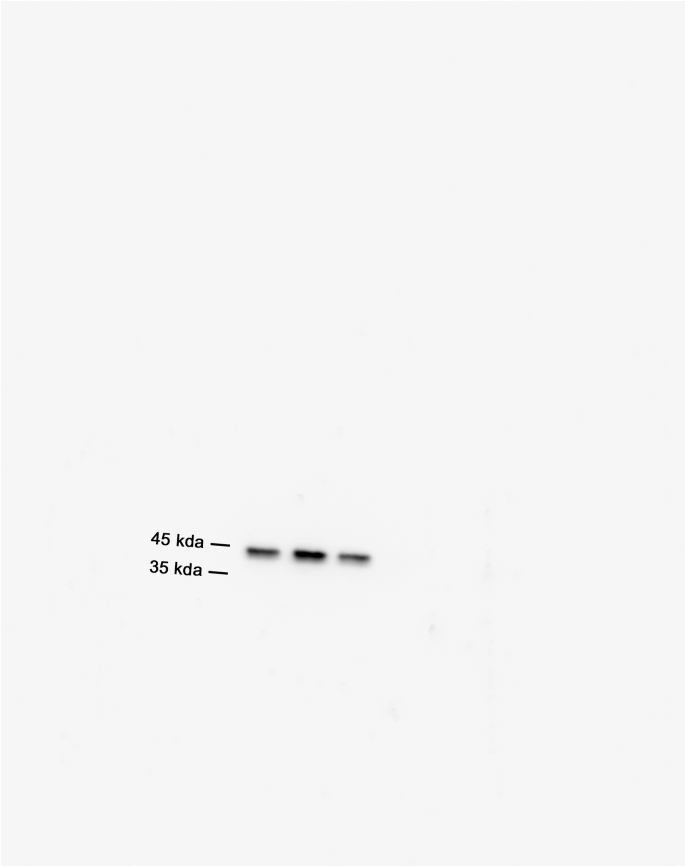
KLF15


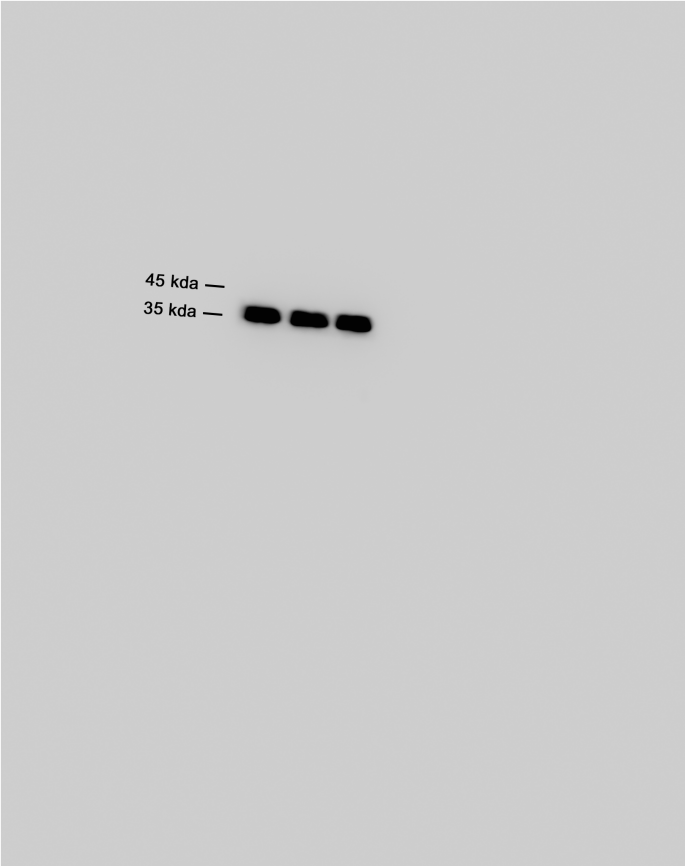
GAPDH

U373


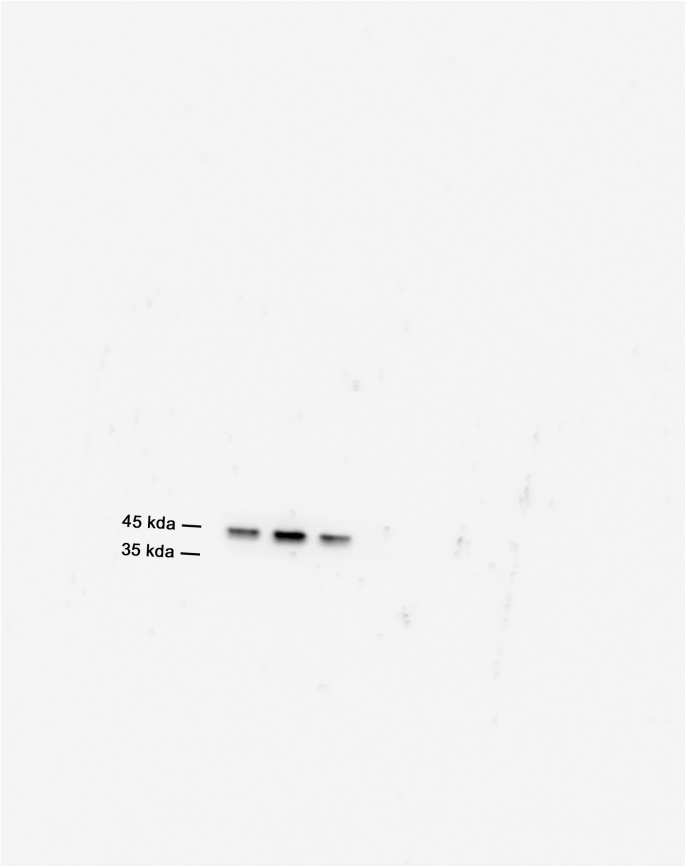
KLF15


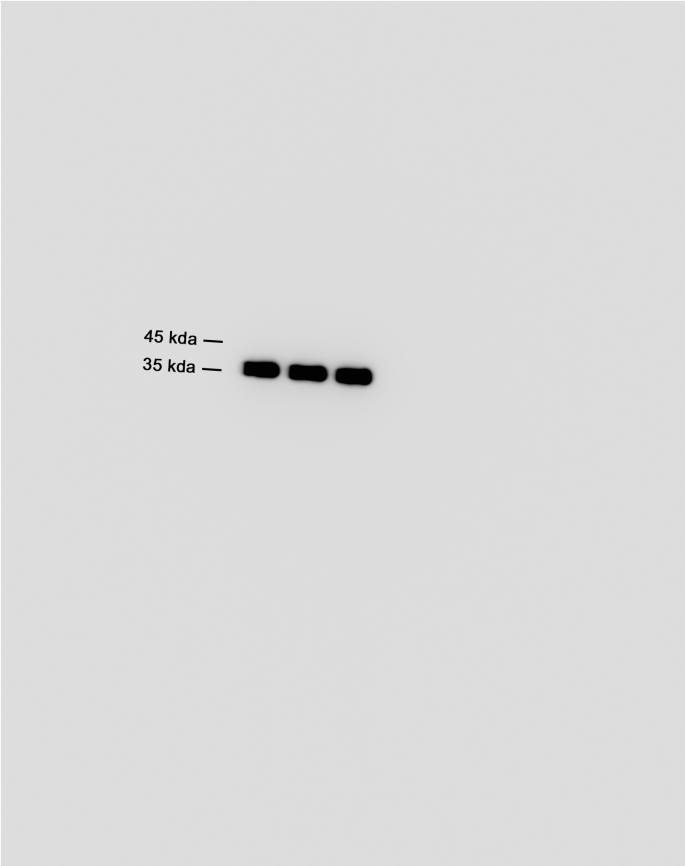
GAPDH

Figure 3g


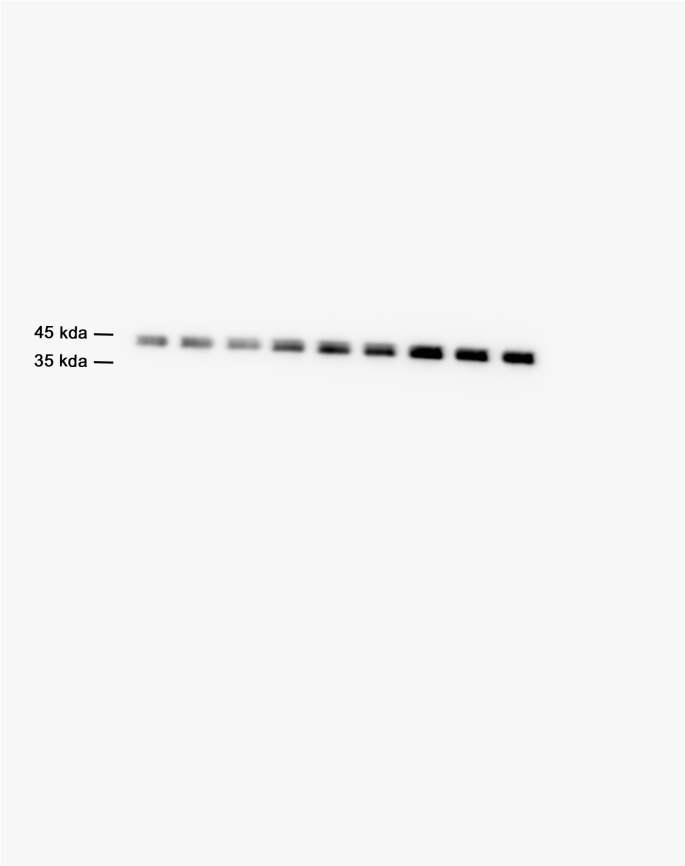
KLF15


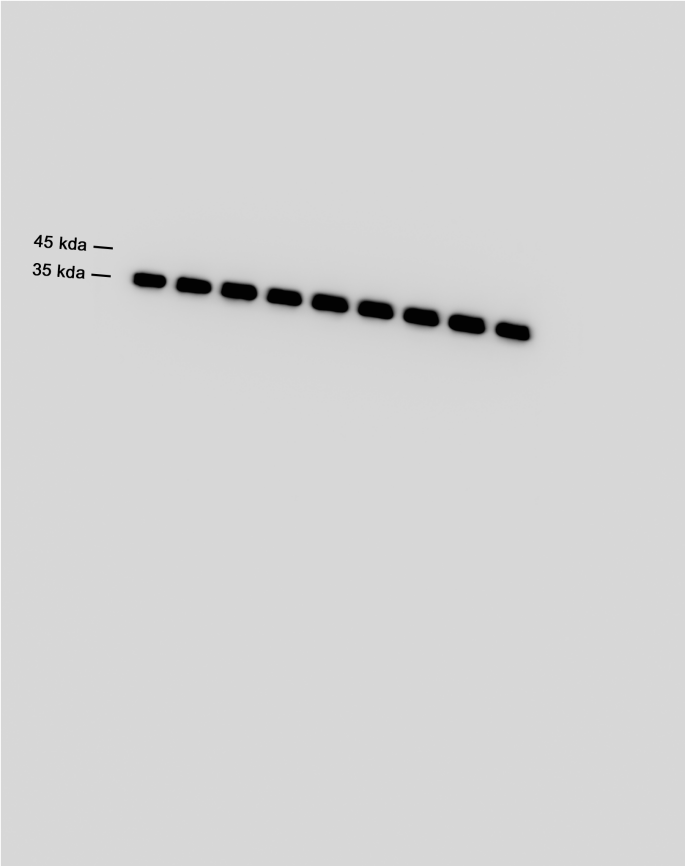
GAPDH


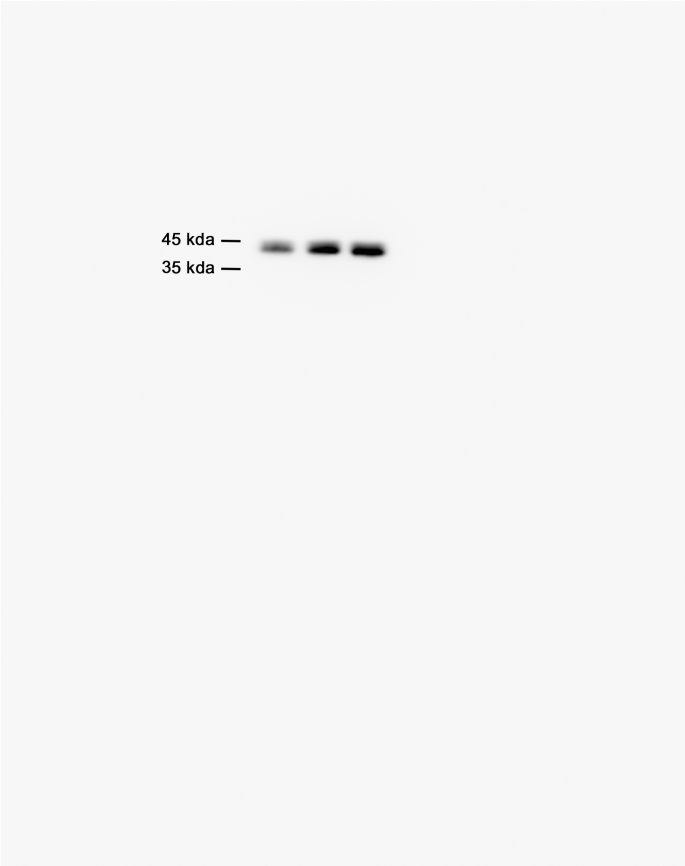
KLF15


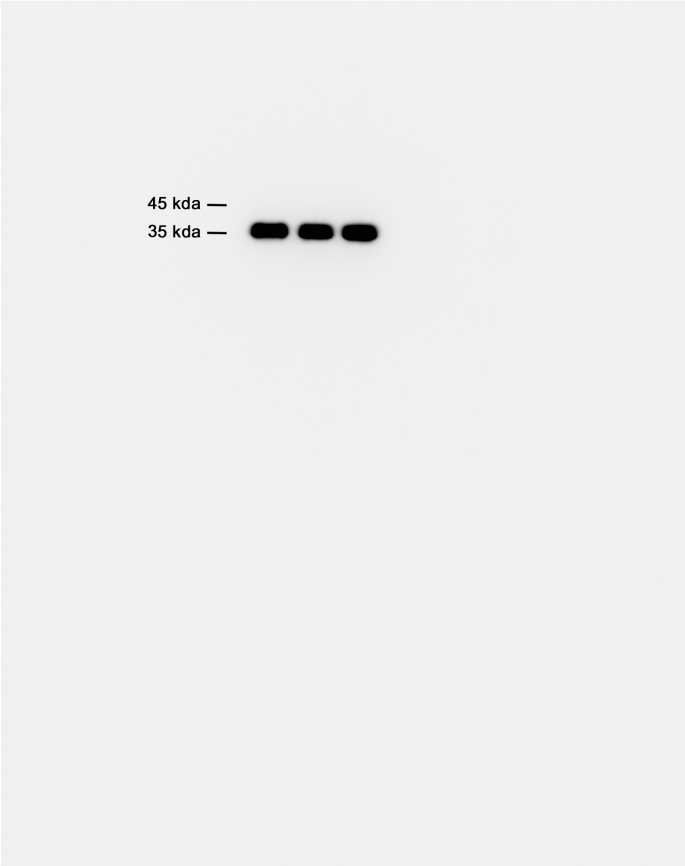
GAPDH

Figure 3h

U251


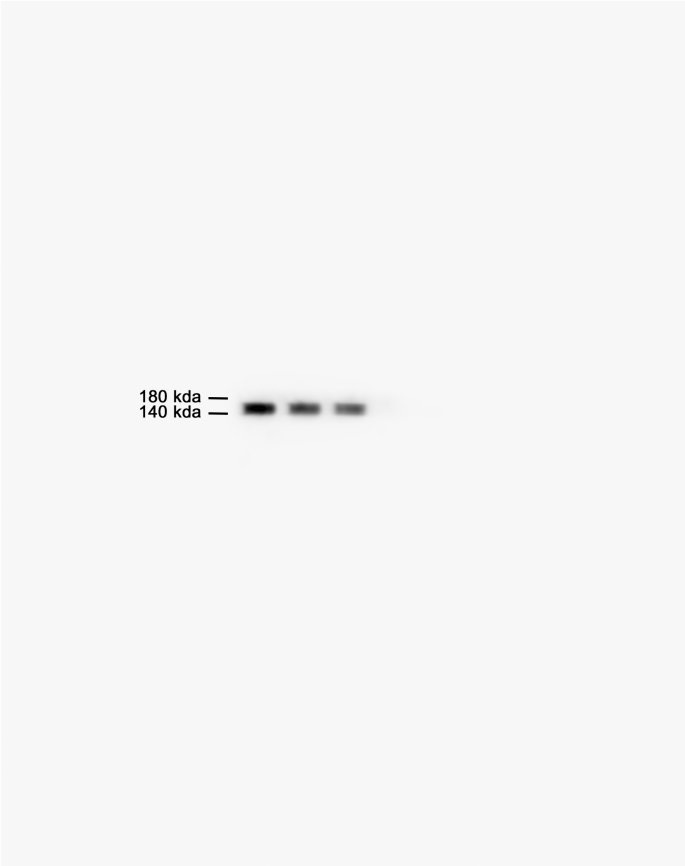
VEGFR2


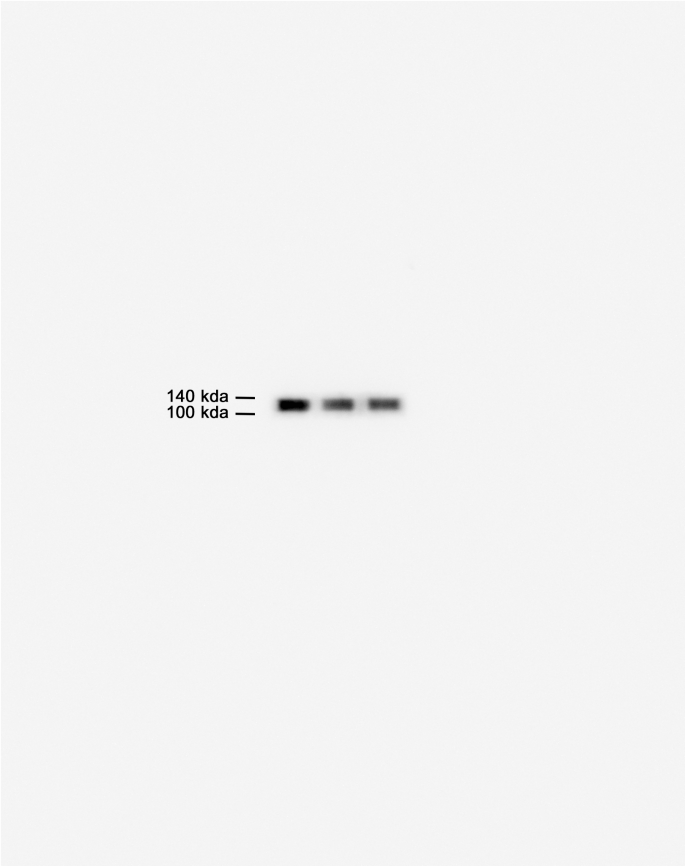
VE-cadherin


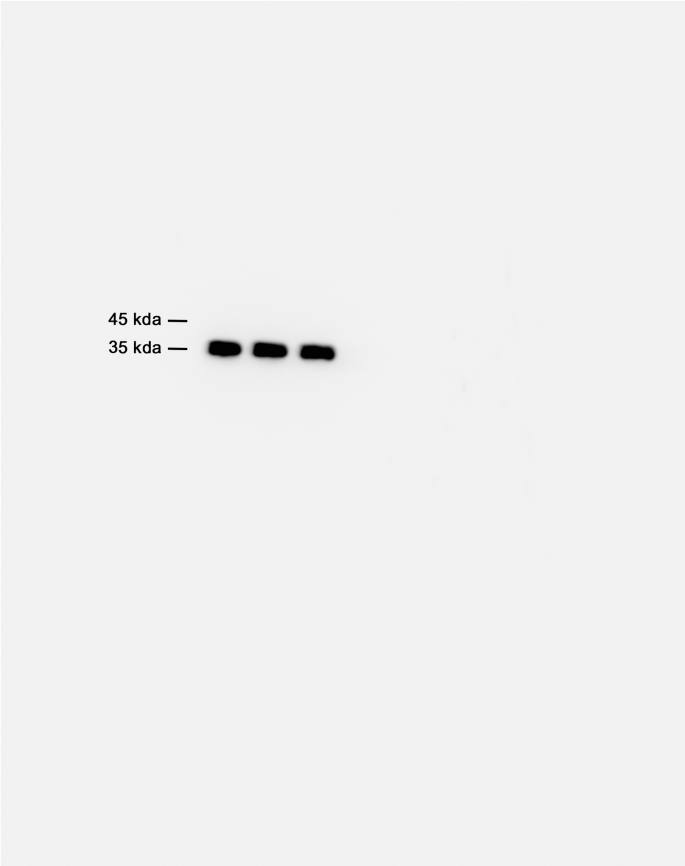
GAPDH

U373


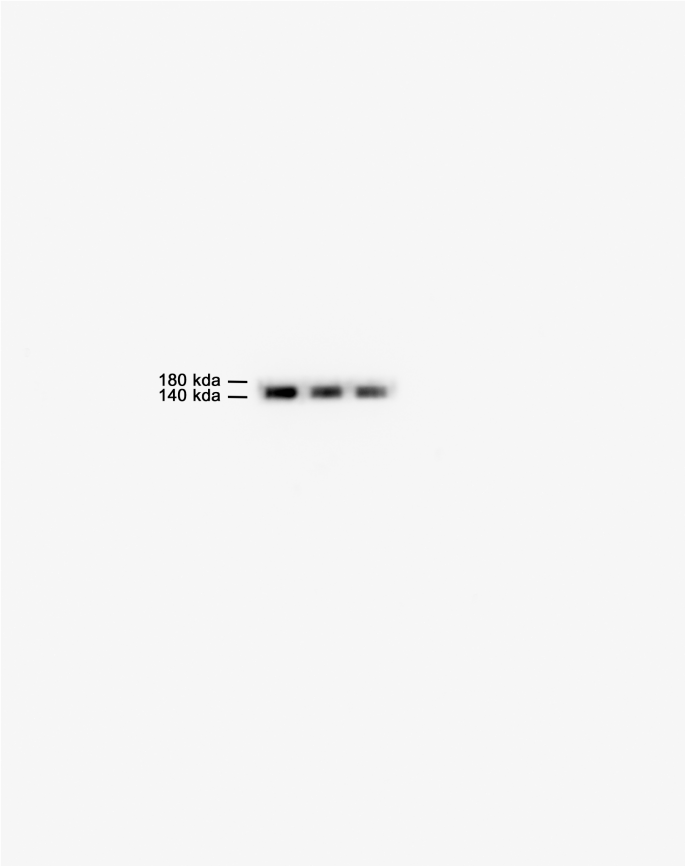
VEGFR2


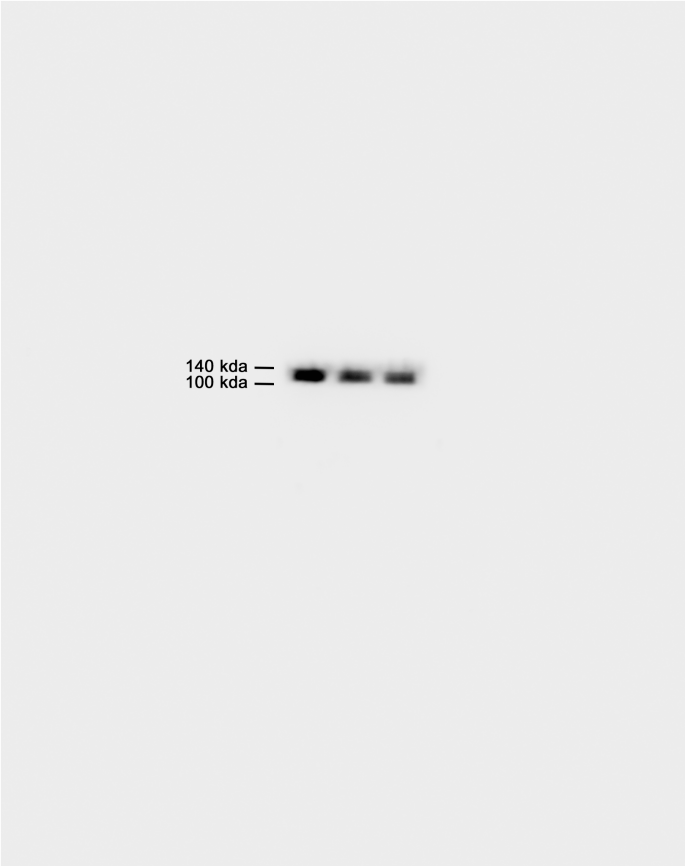
VE-cadherin


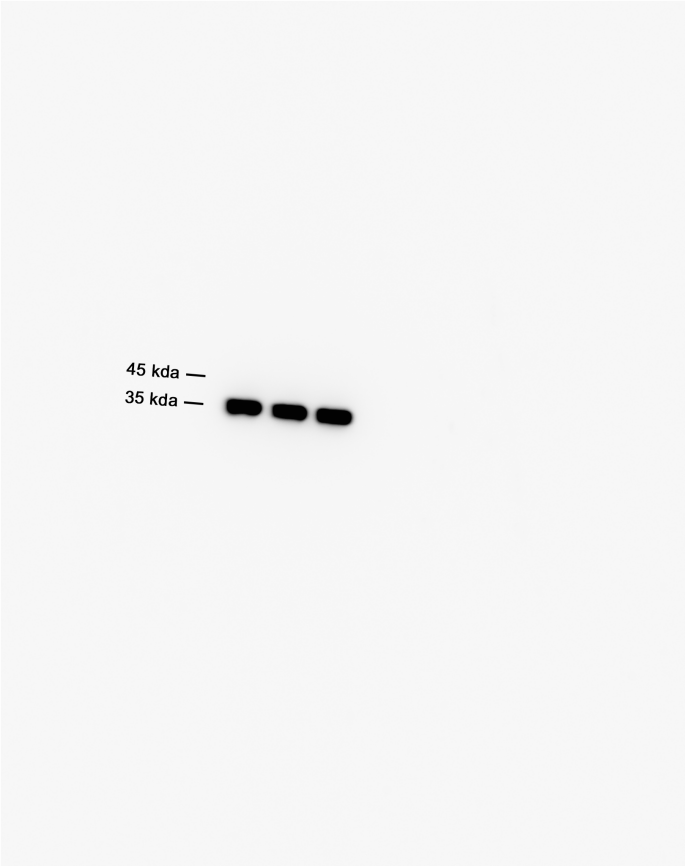
GAPDH

Figure 4a


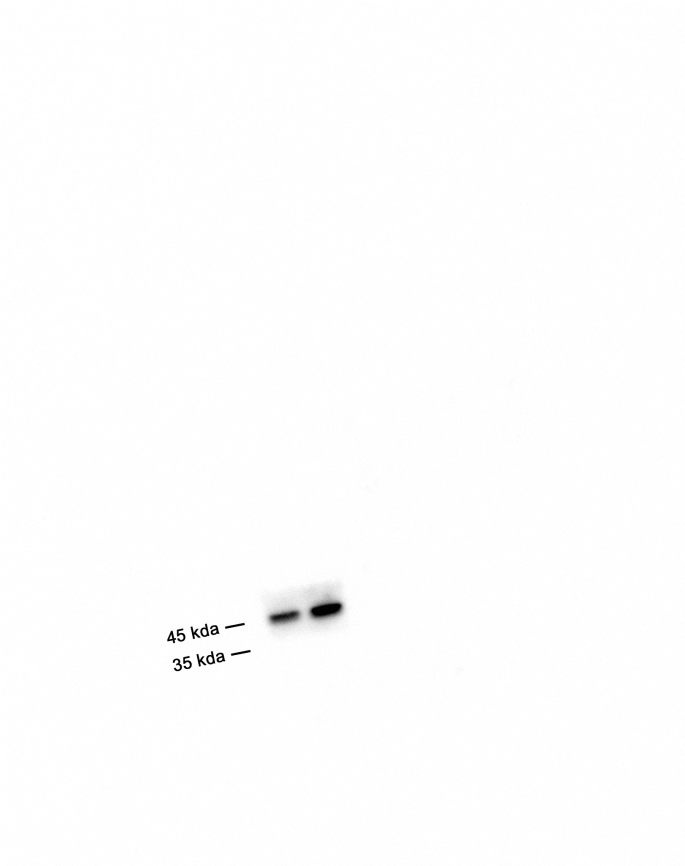
WT-p-Ser


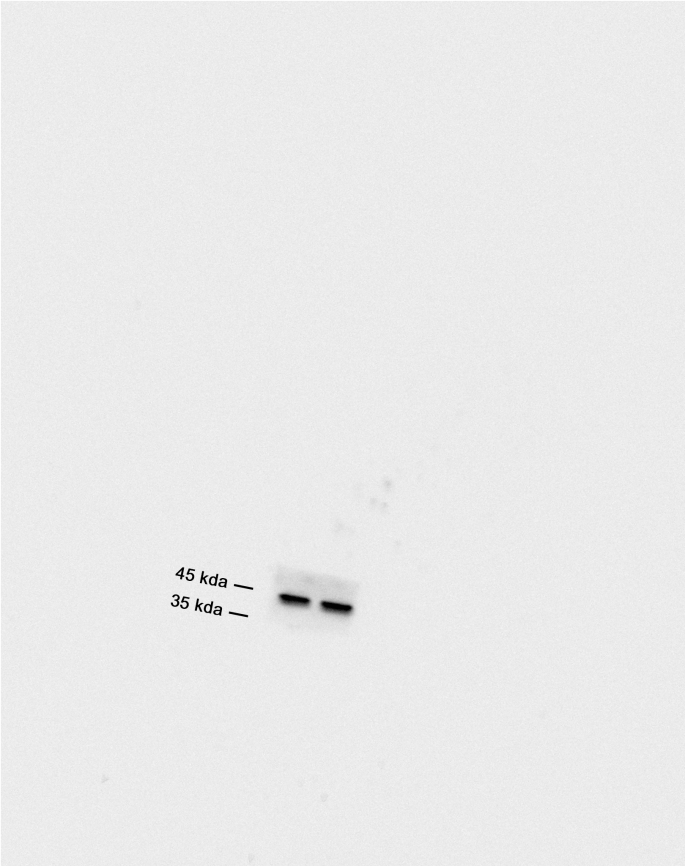
WT-KLF15-IP


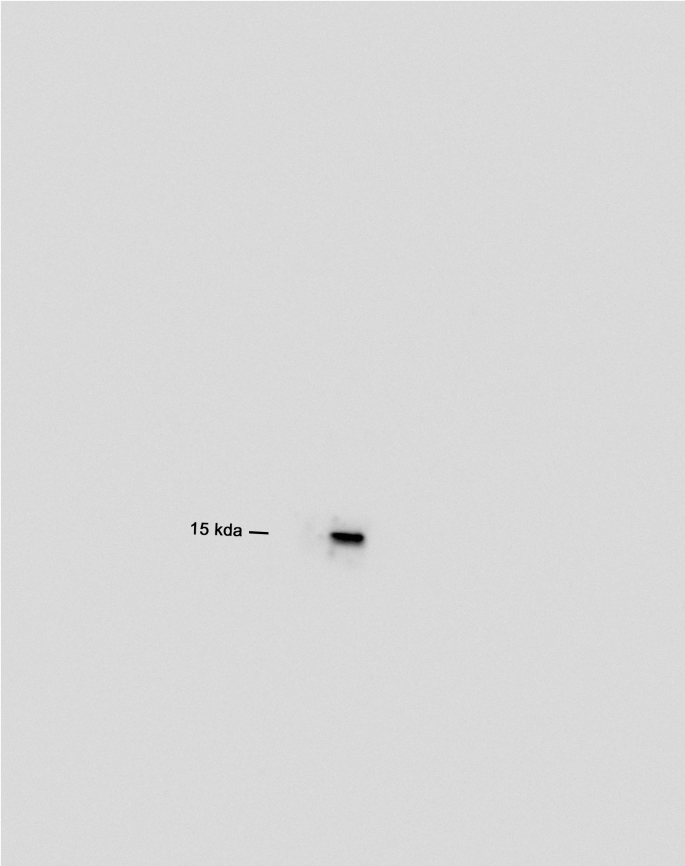
WT-P4-135aa


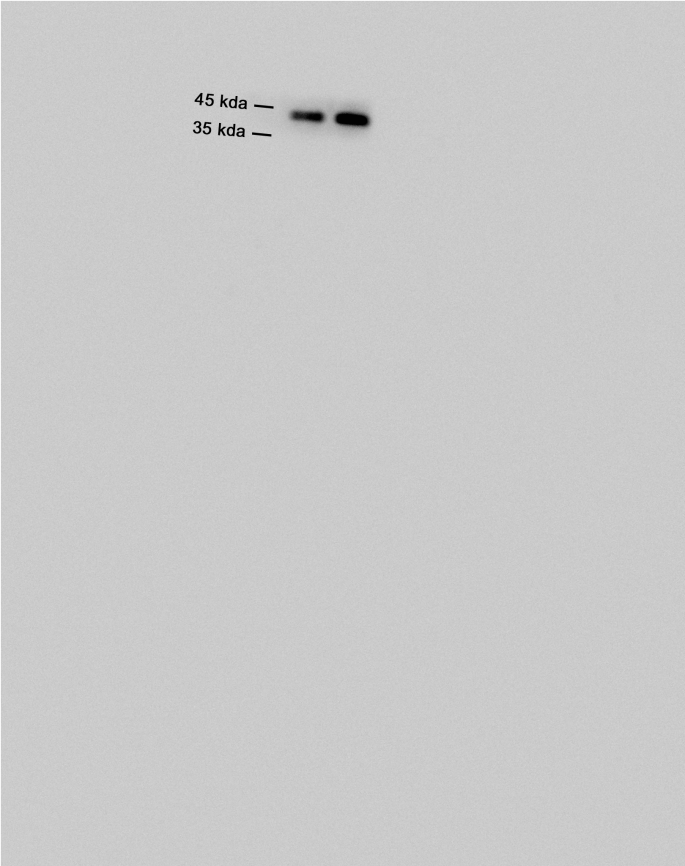
WT-KLF15-WCL


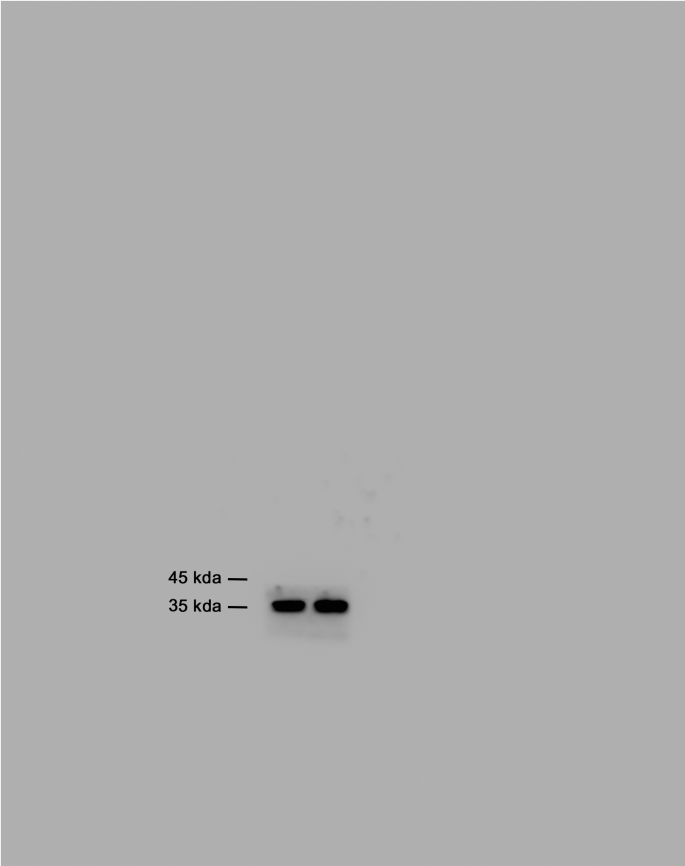
WT-GAPDH


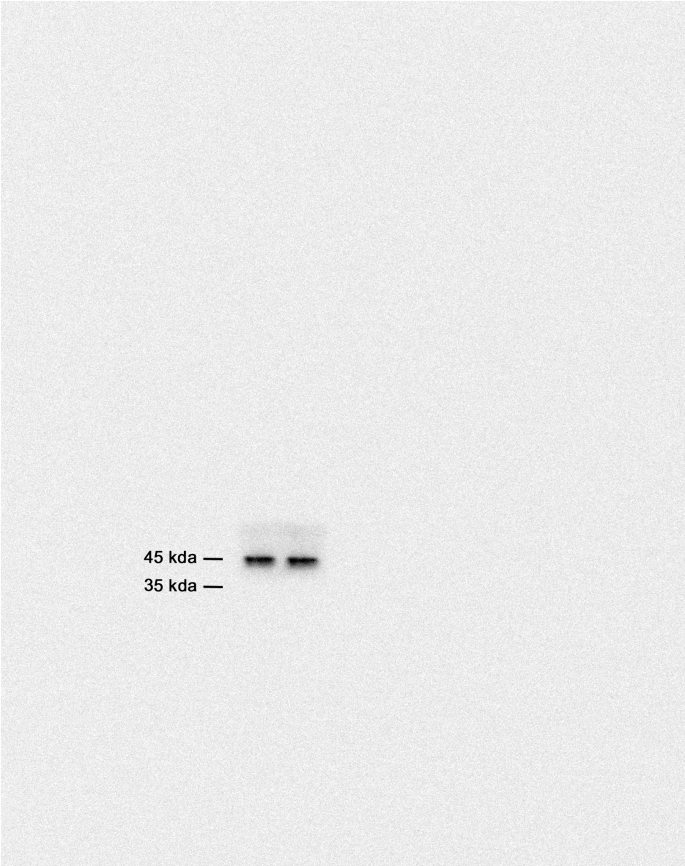
S238A-p-Ser


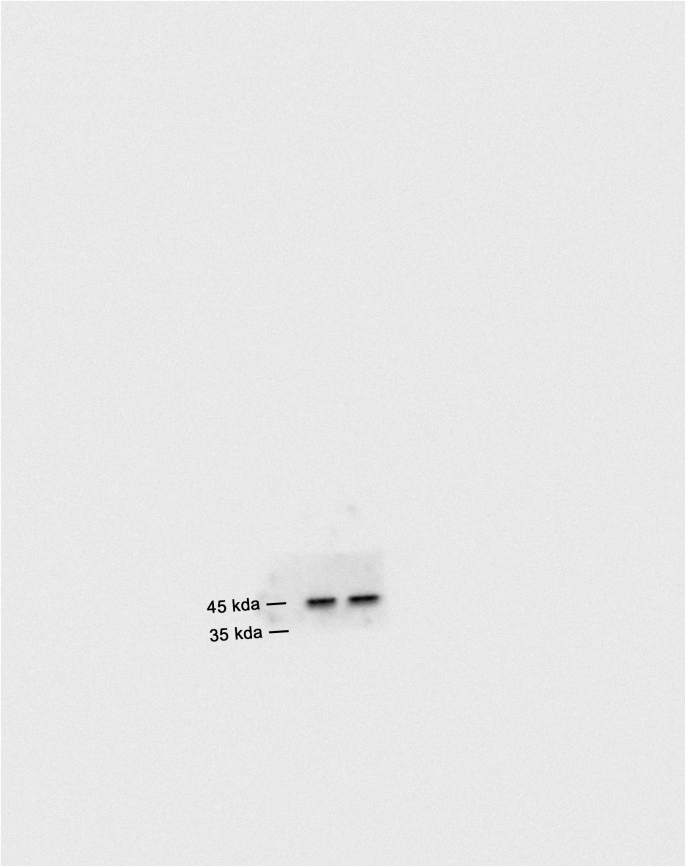
S238A-KLF15-IP


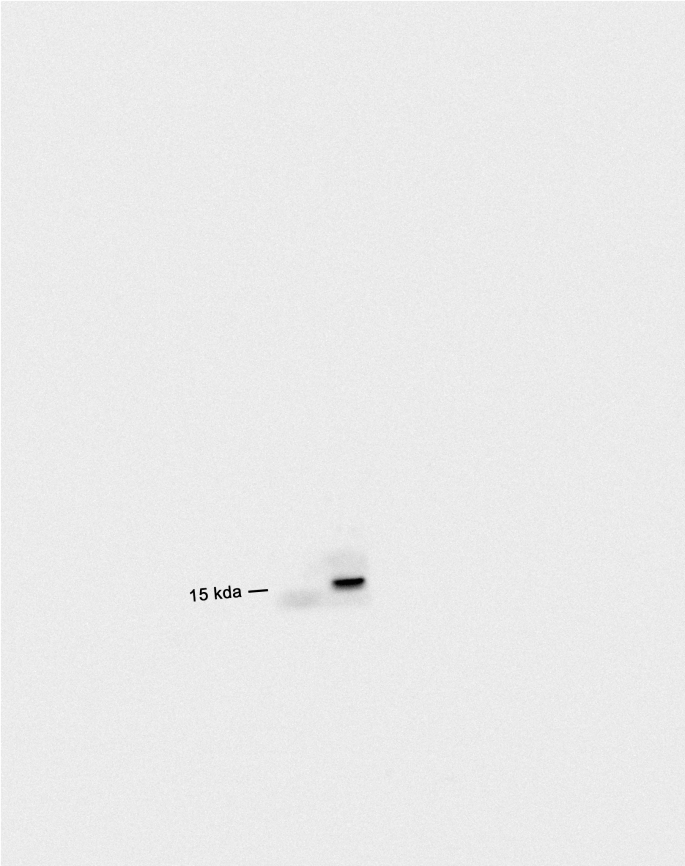
S238A-P4-135aa


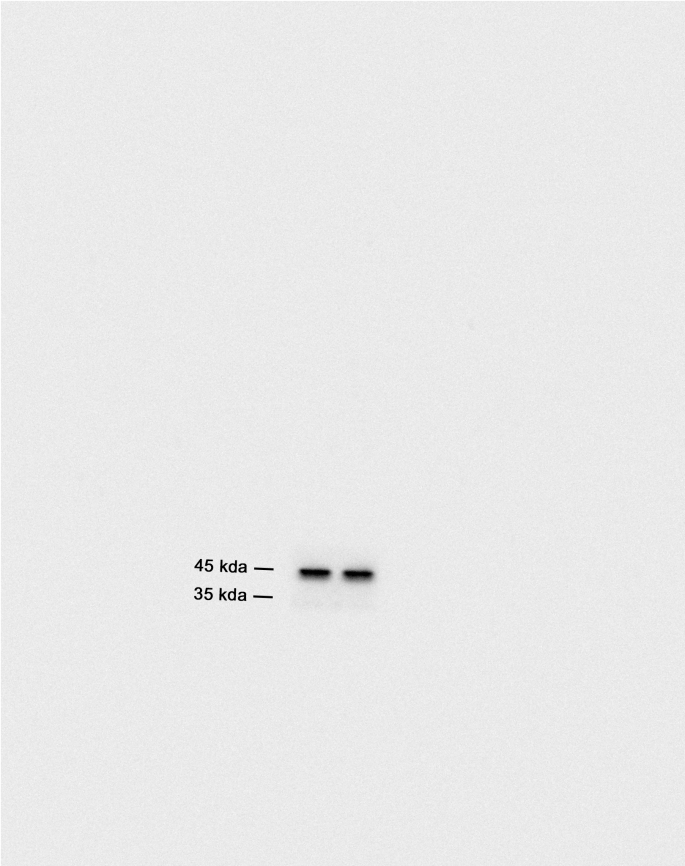
S238A-KLF15-WCL


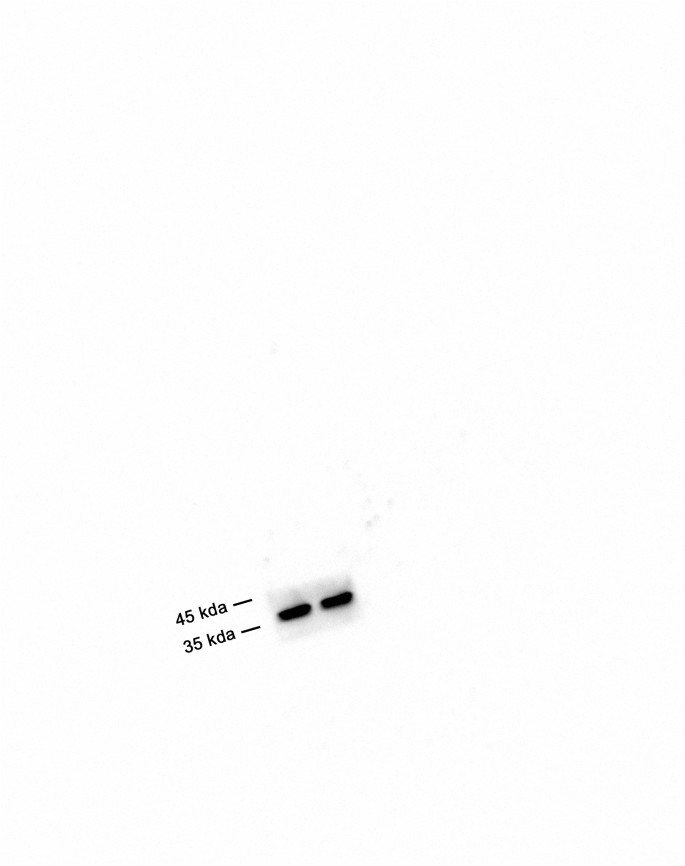
S238A-GAPDH

Figure 4b


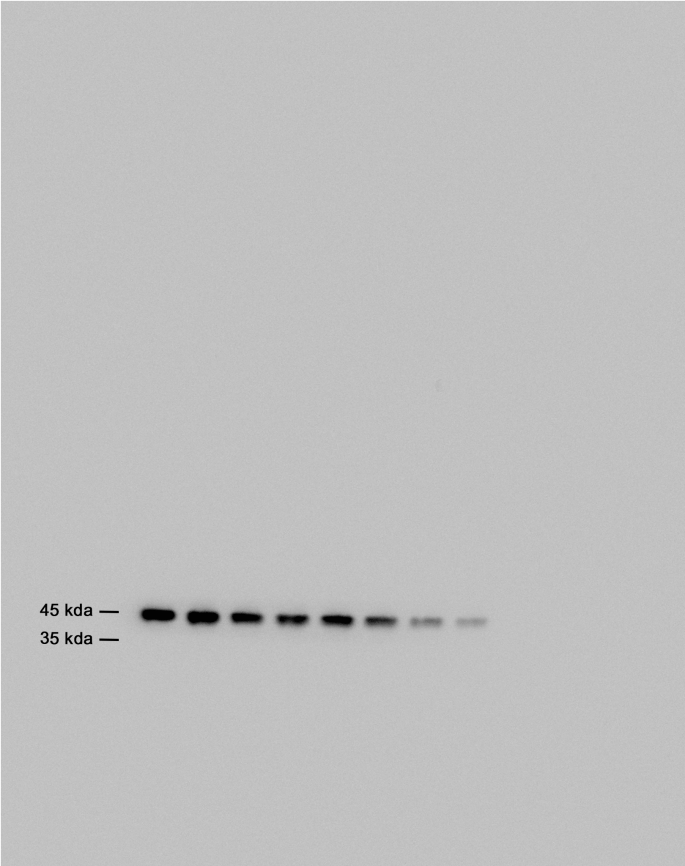
KLF15


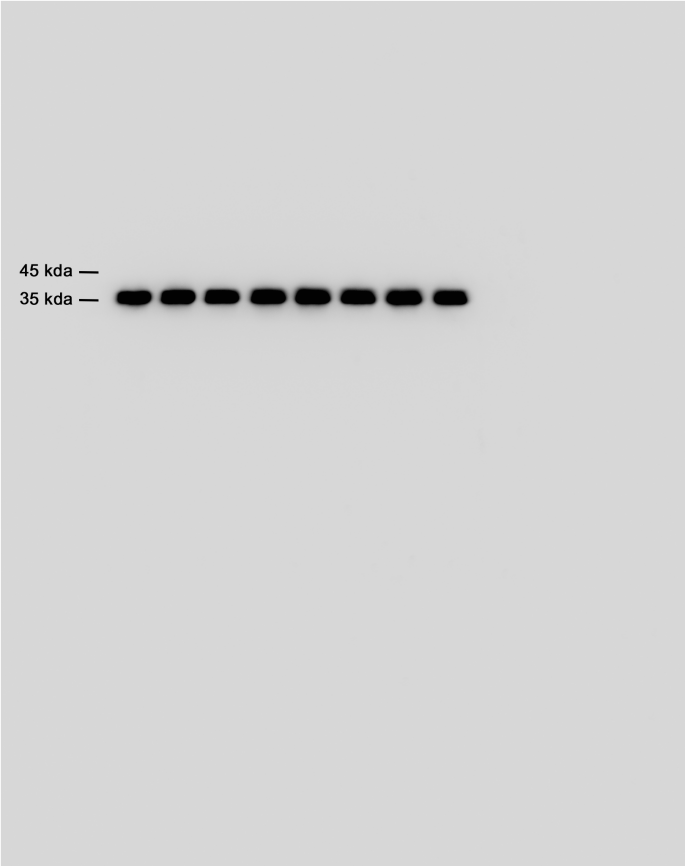
GAPDH

Figure 5b

U251


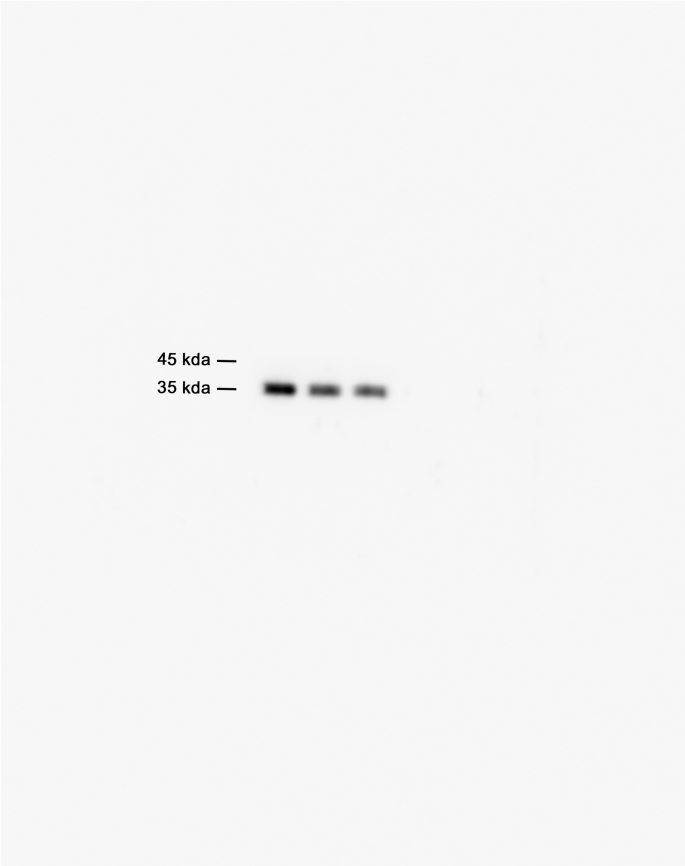
LDHA


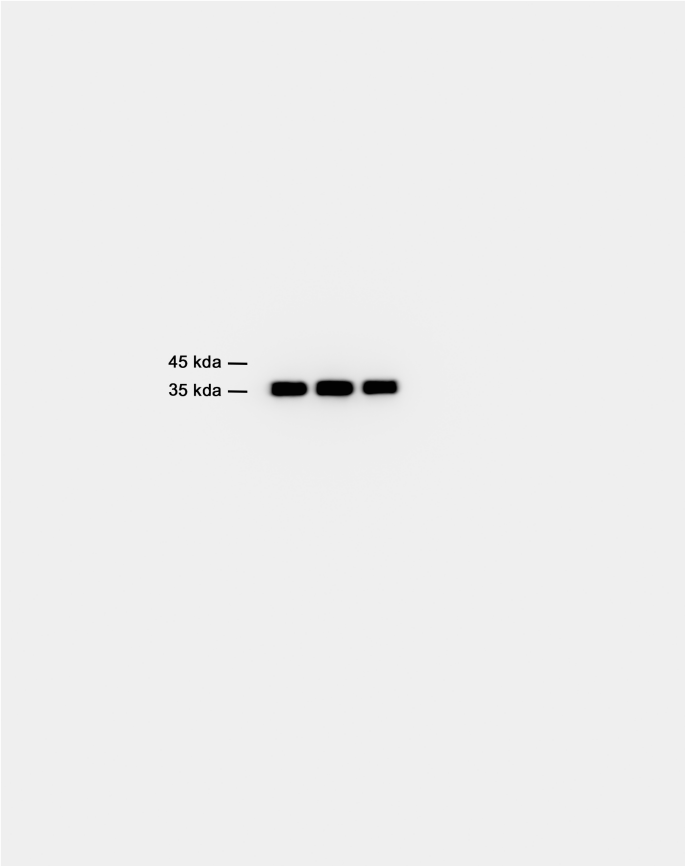
GAPDH

U373


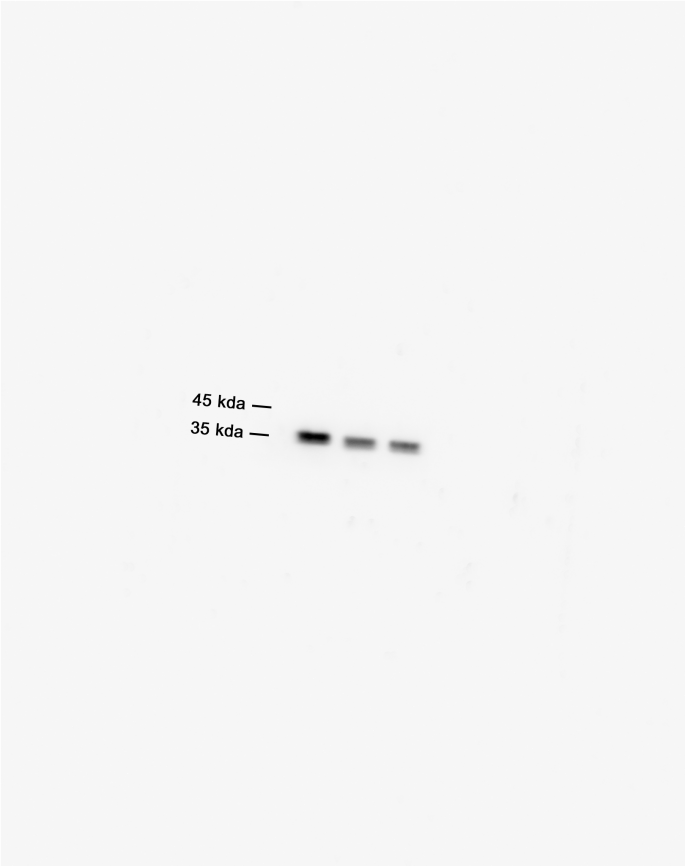
LDHA


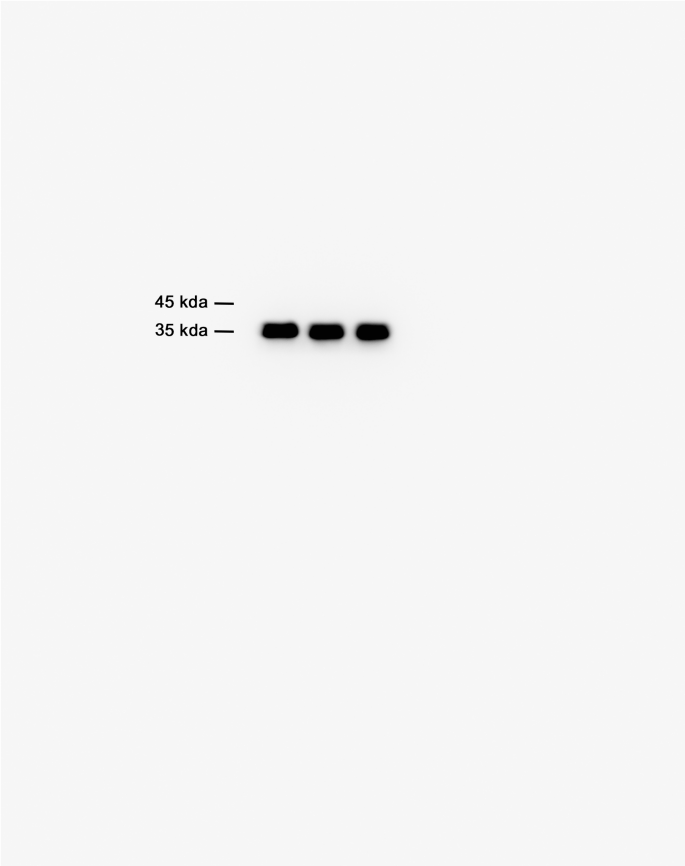
GAPDH

Figure 5c

U251


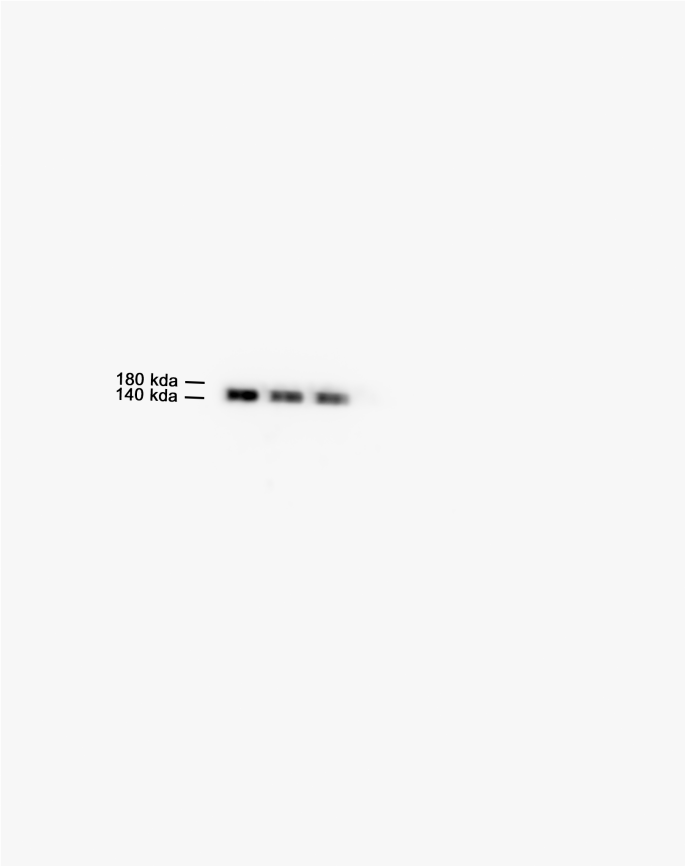
VEGFR2


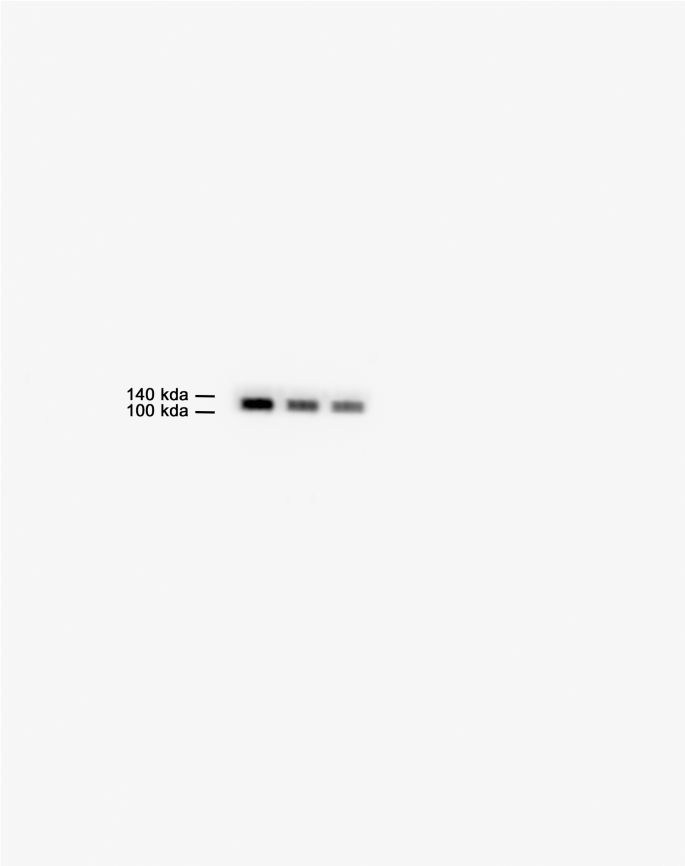
VE-cadherin


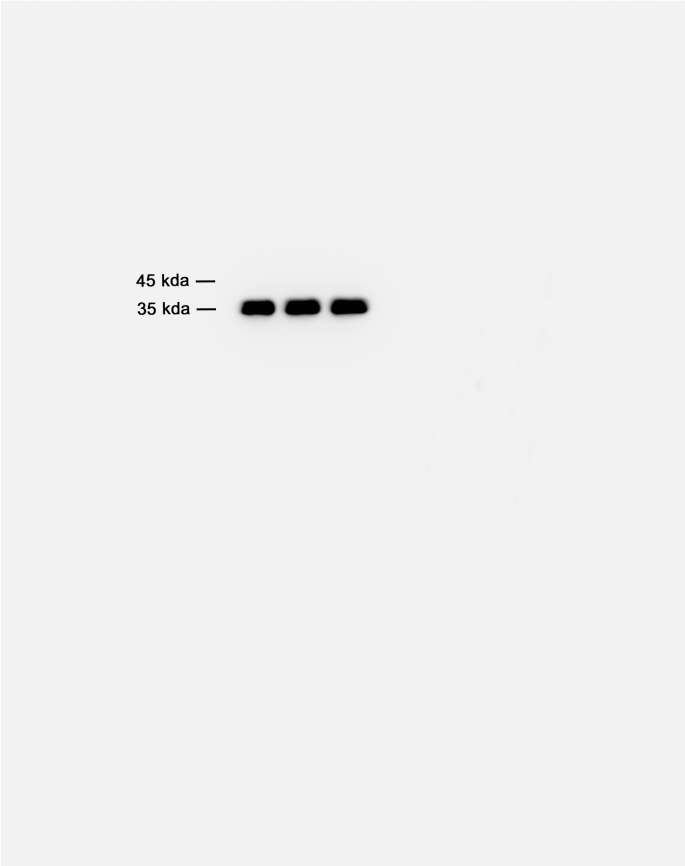
GAPDH

U373


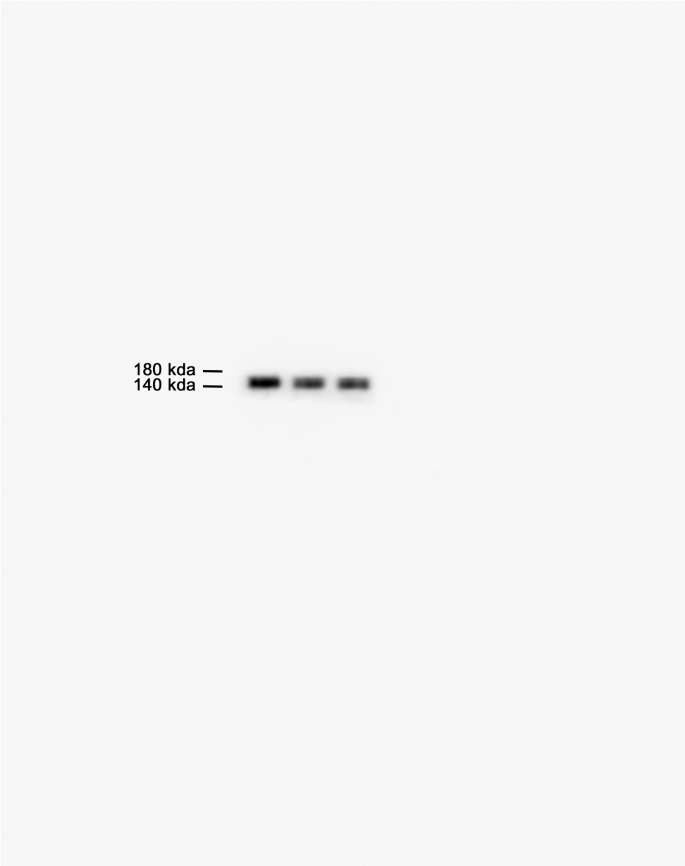
VEGFR2


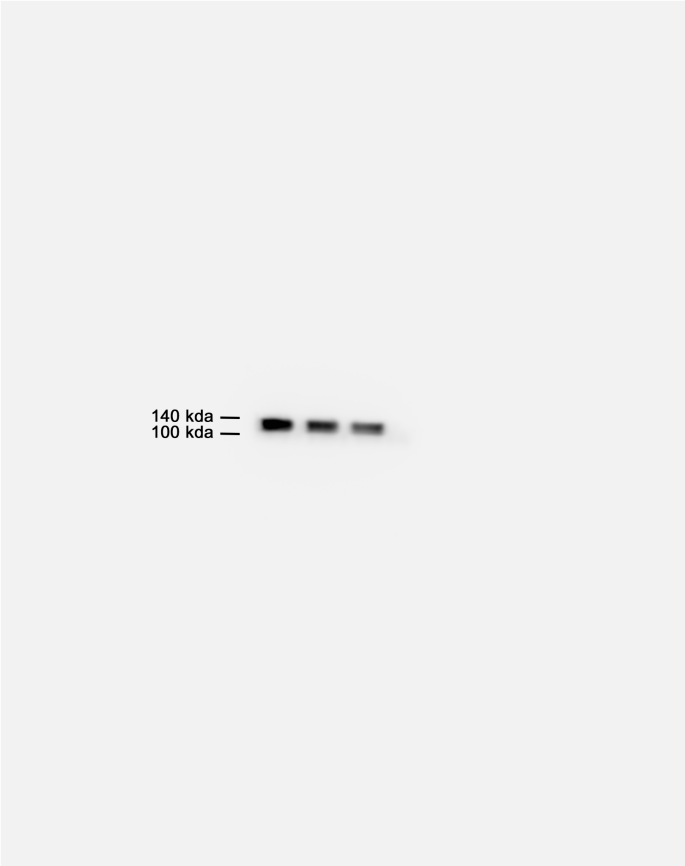
VE-cadherin


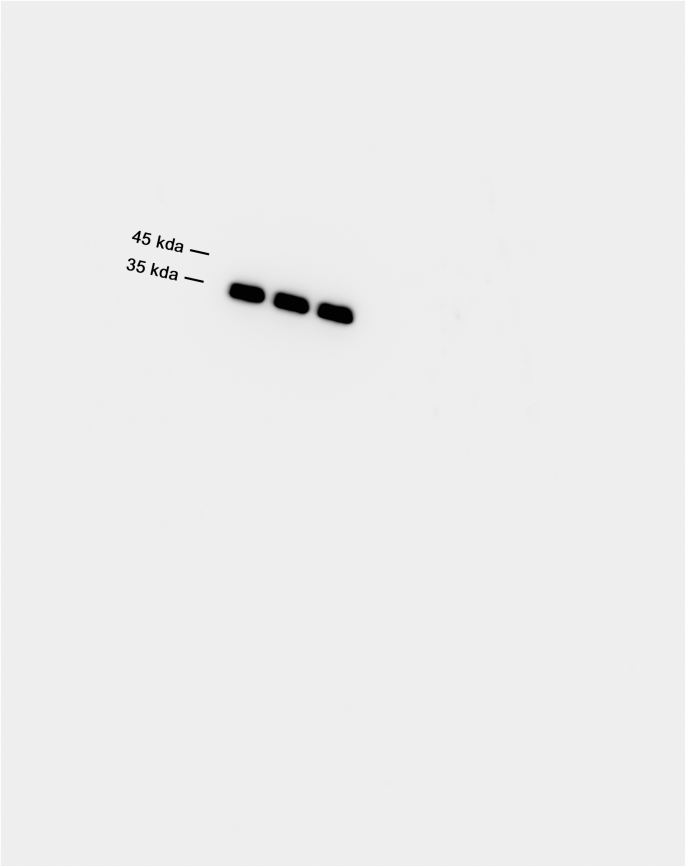
GAPDH

Figure 6c

U251


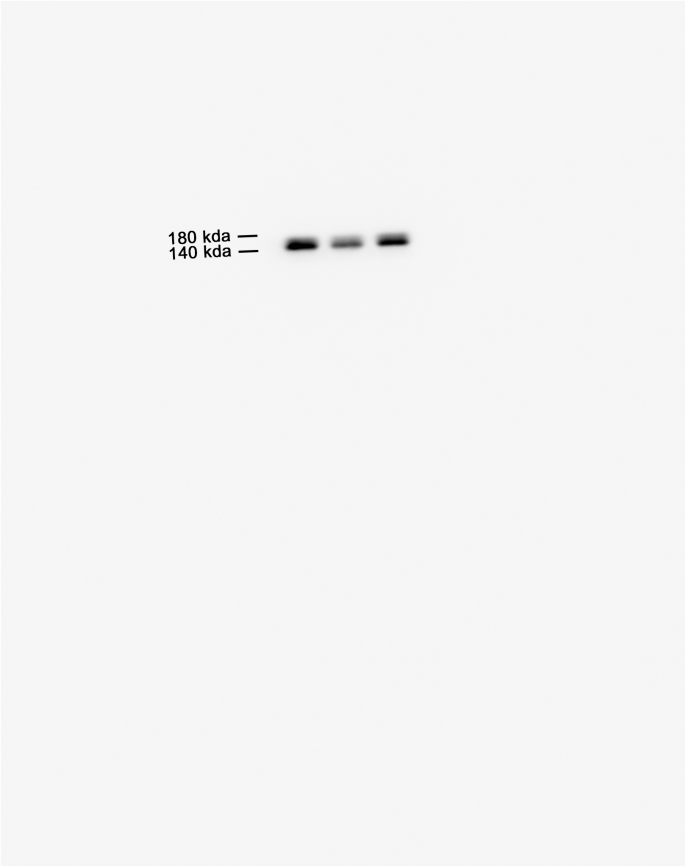
VEGFR2


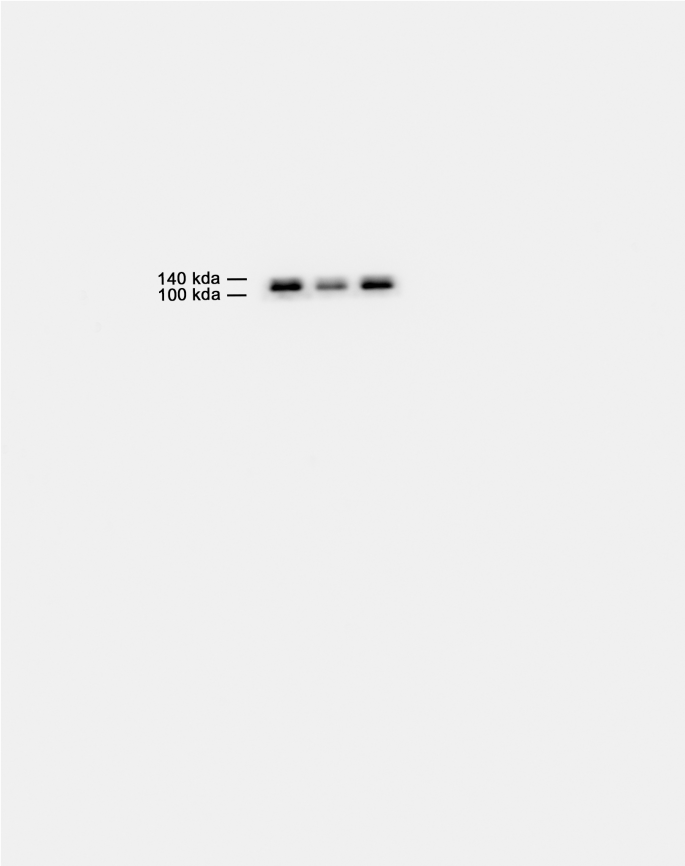
VE-cadherin


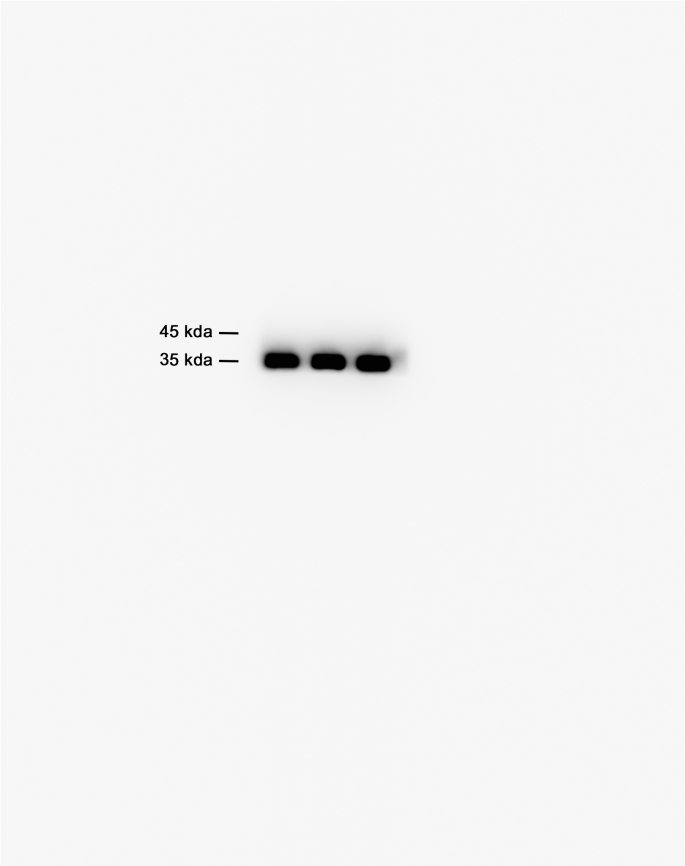
GAPDH

U373


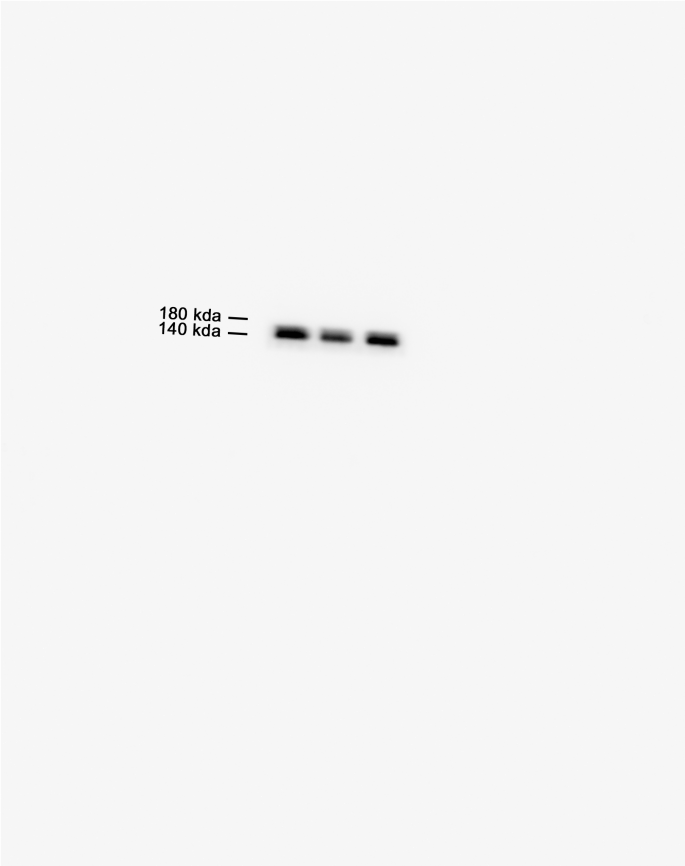
VEGFR2


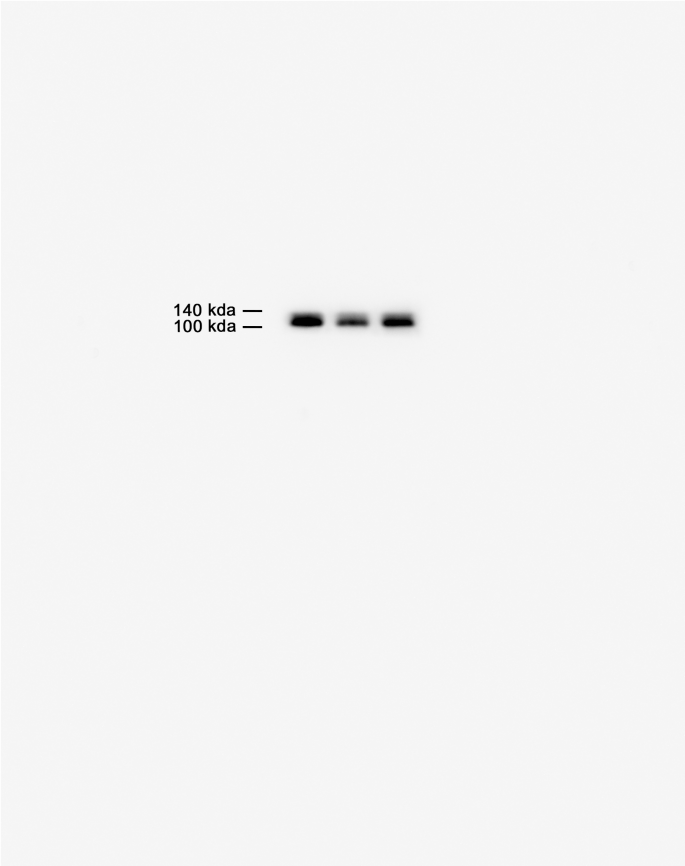
VE-cadherin


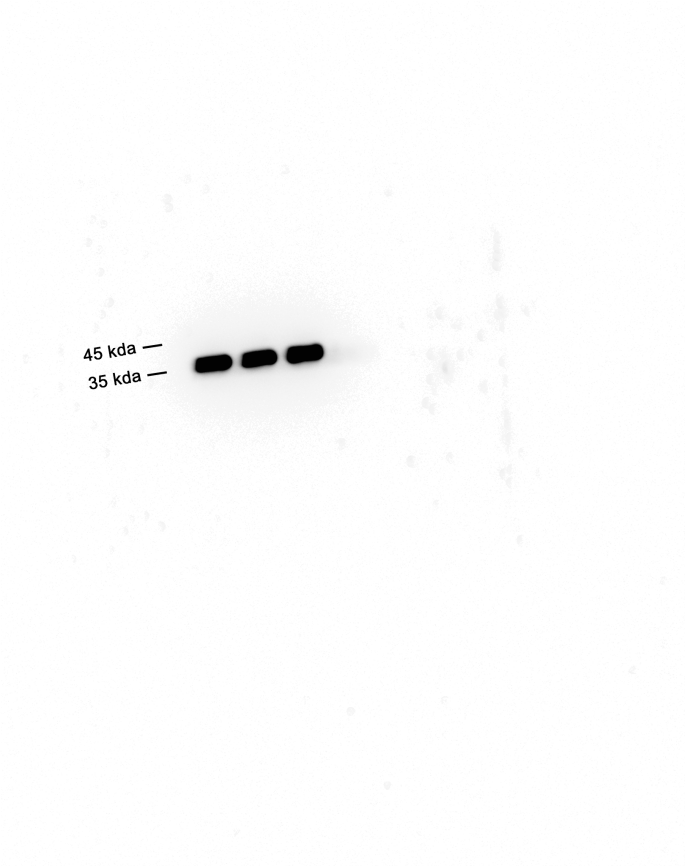
GAPDH

Figure S1.1 b


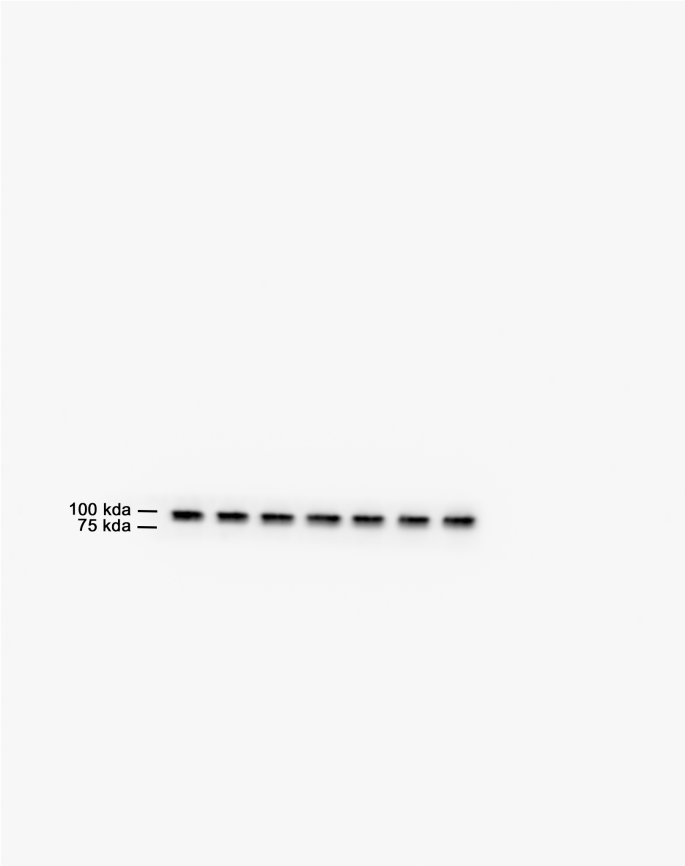
MAPK6


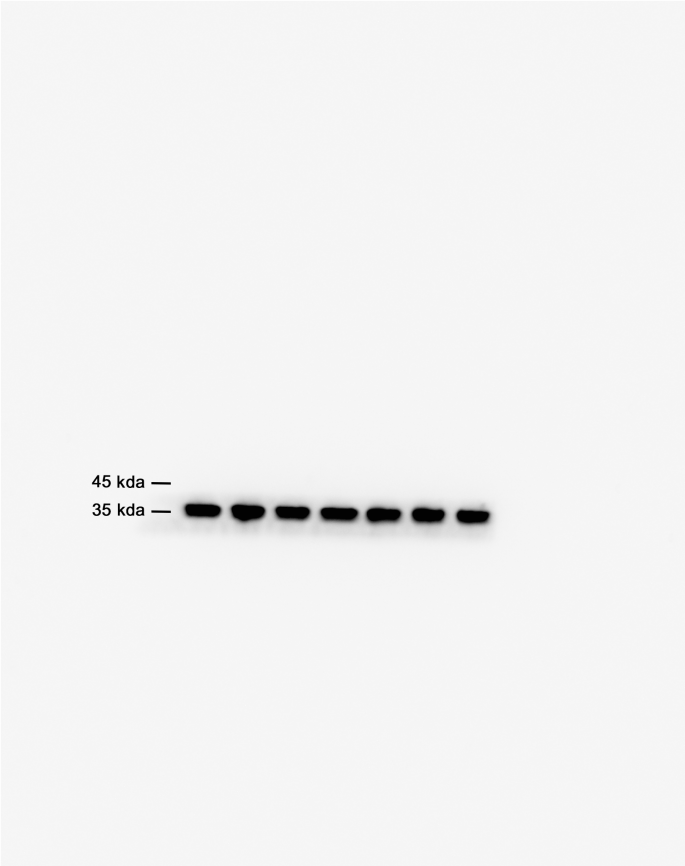
GAPDH

Figure S1.1 g

U251


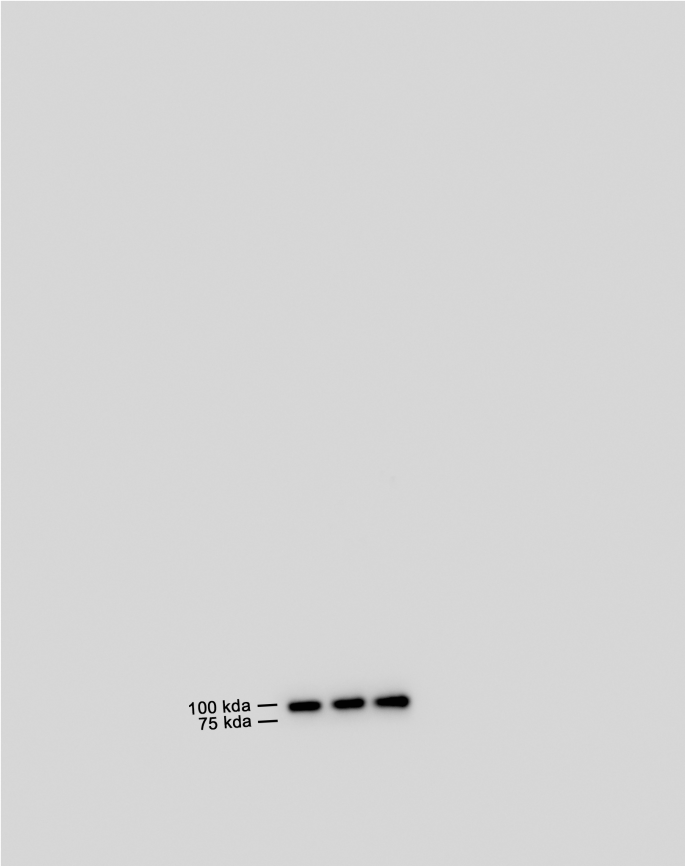
MAPK6


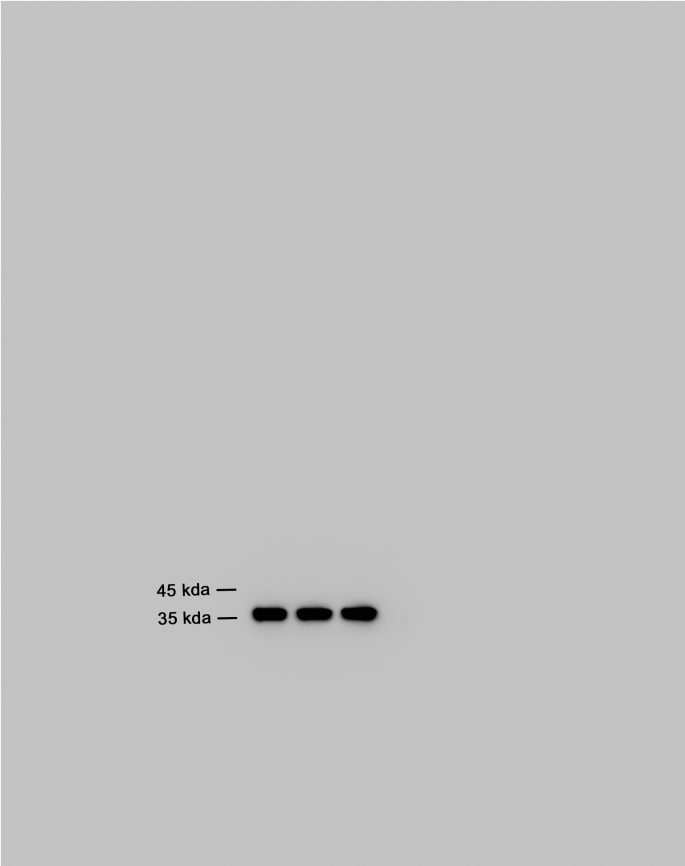
GAPDH

U373


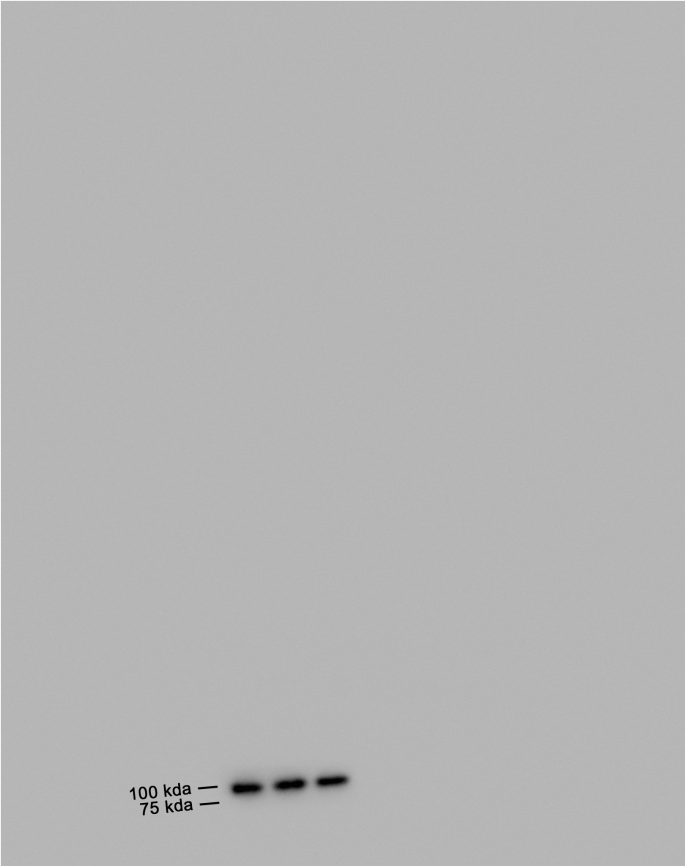
MAPK6


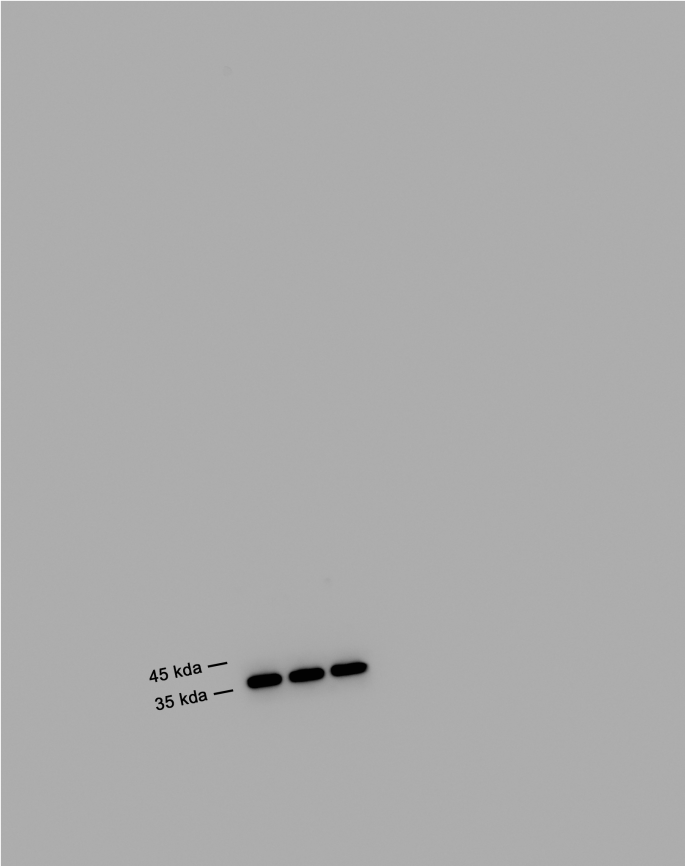
GAPDH

Figure S1.2 c


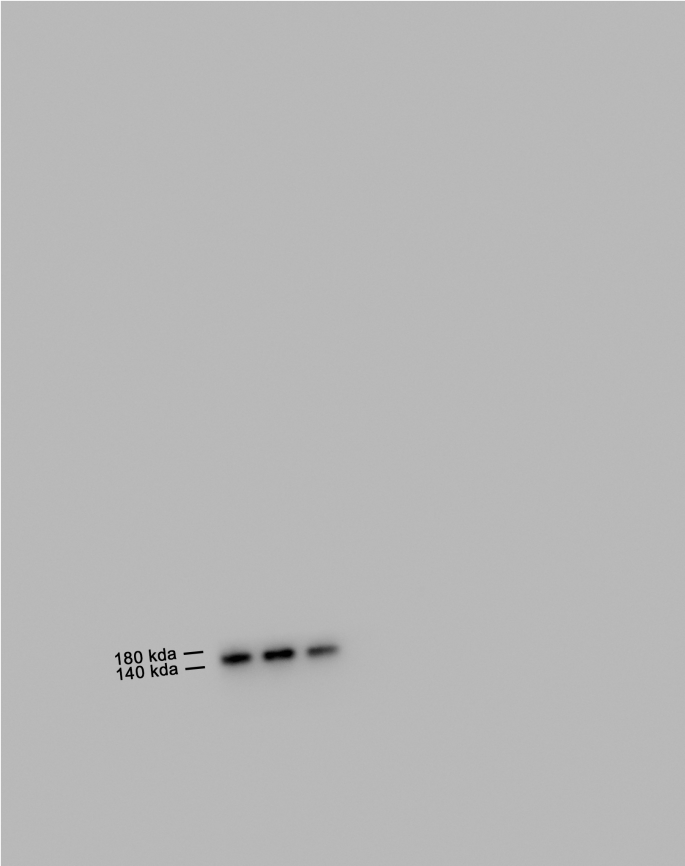
VEGFR2


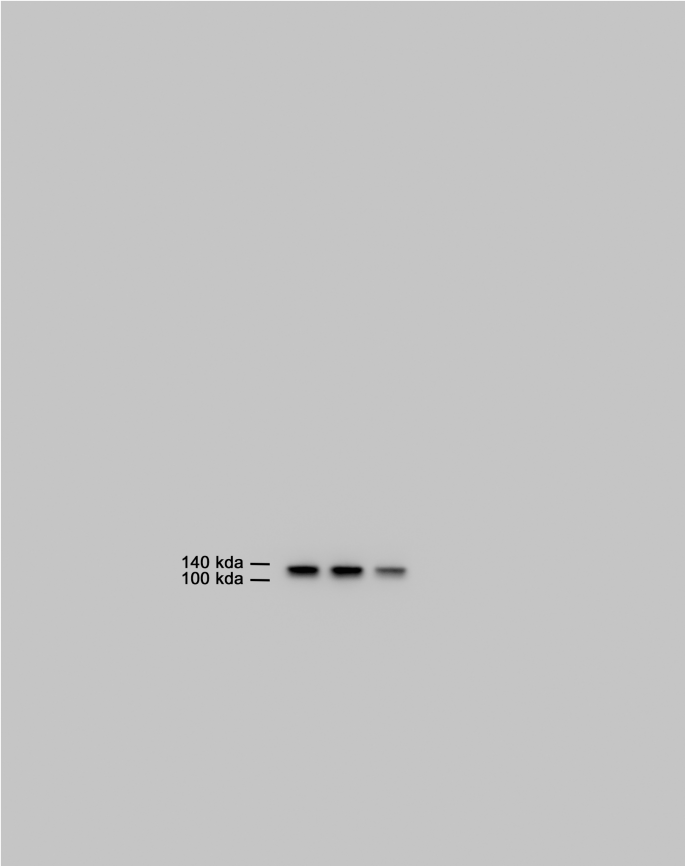
VE-cadherin


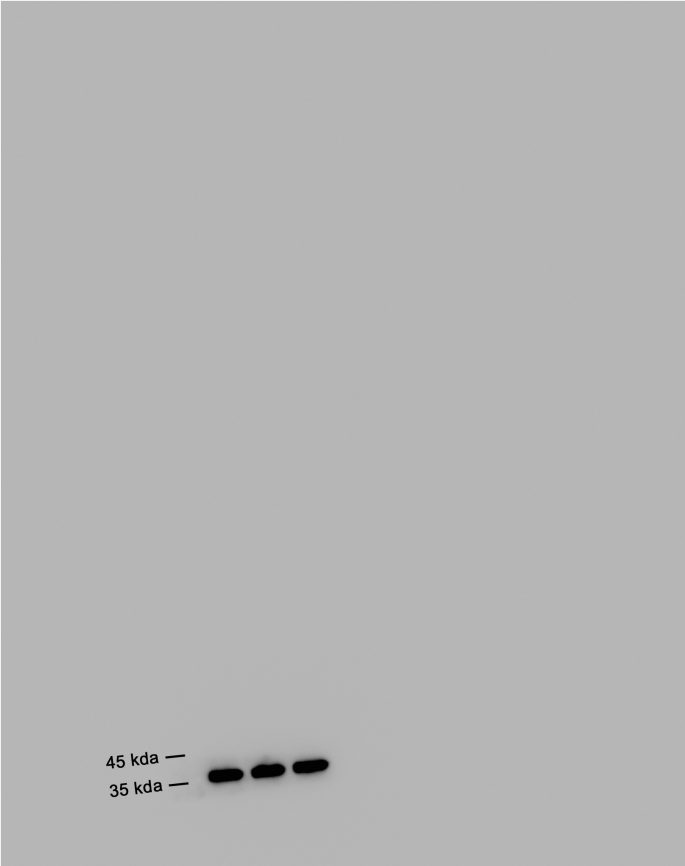
GAPDH

Figure S1.2 h


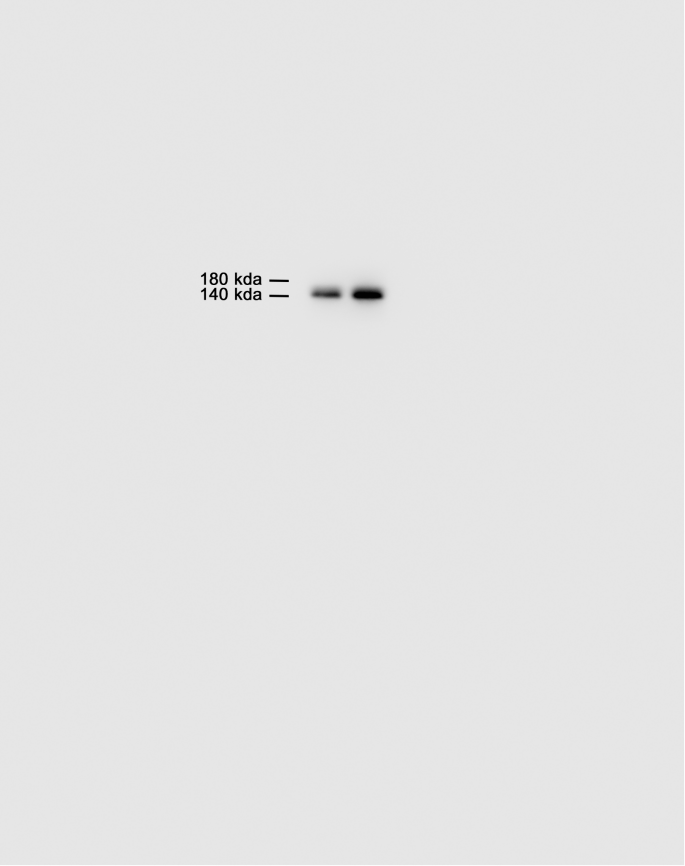
VEGFR2


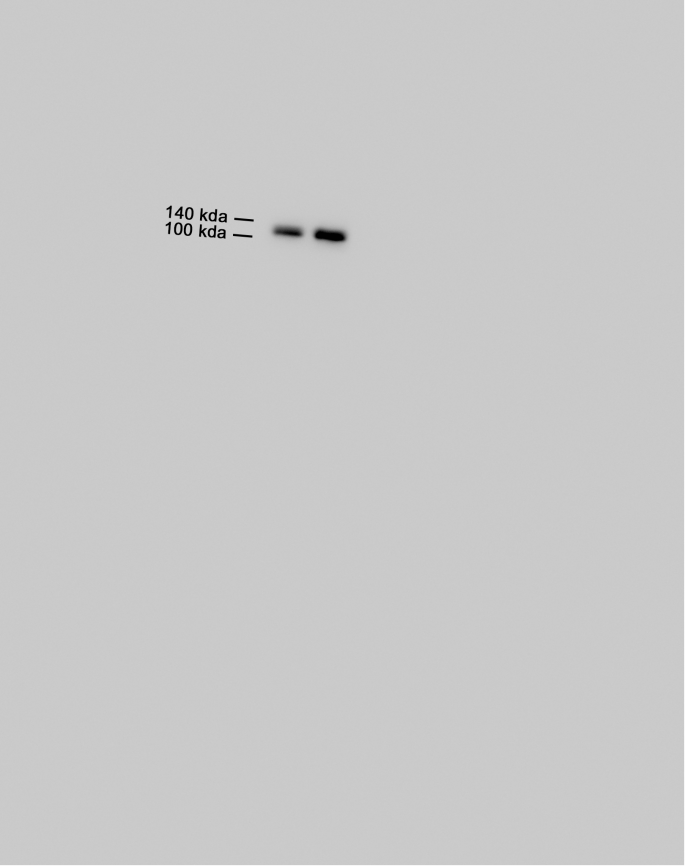
VE-cadherin


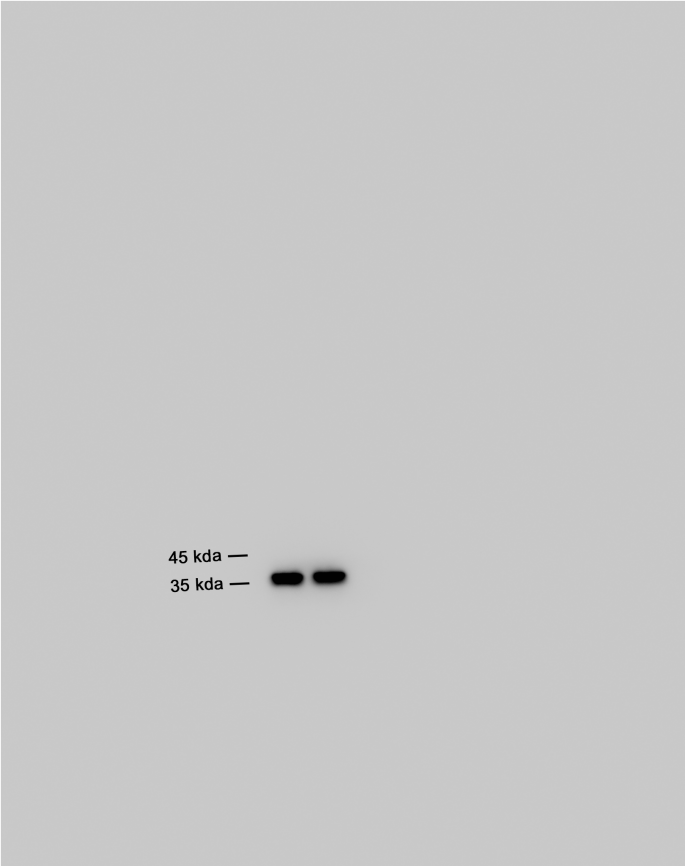
GAPDH

Figure S2.1 c

U251


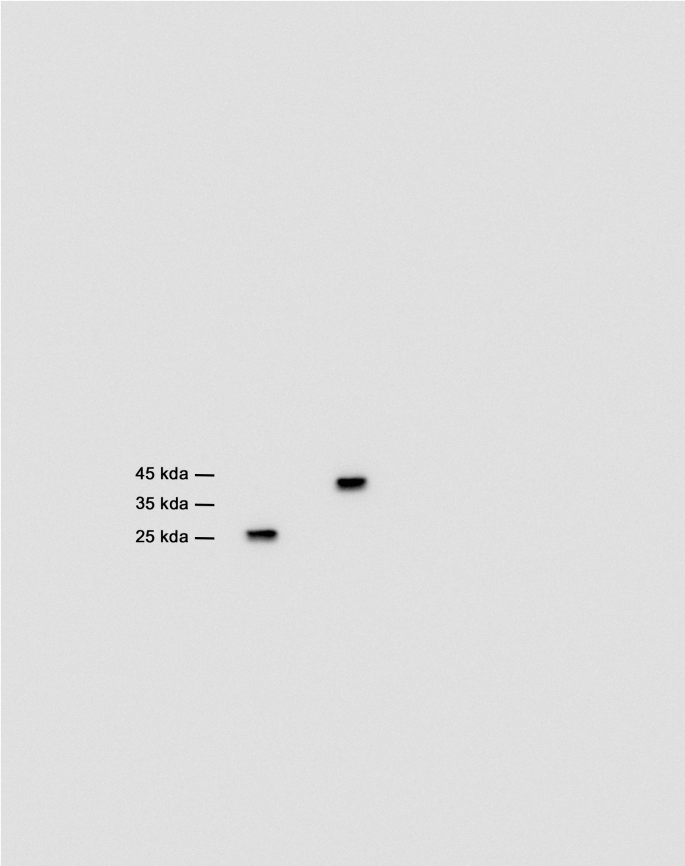
GFP


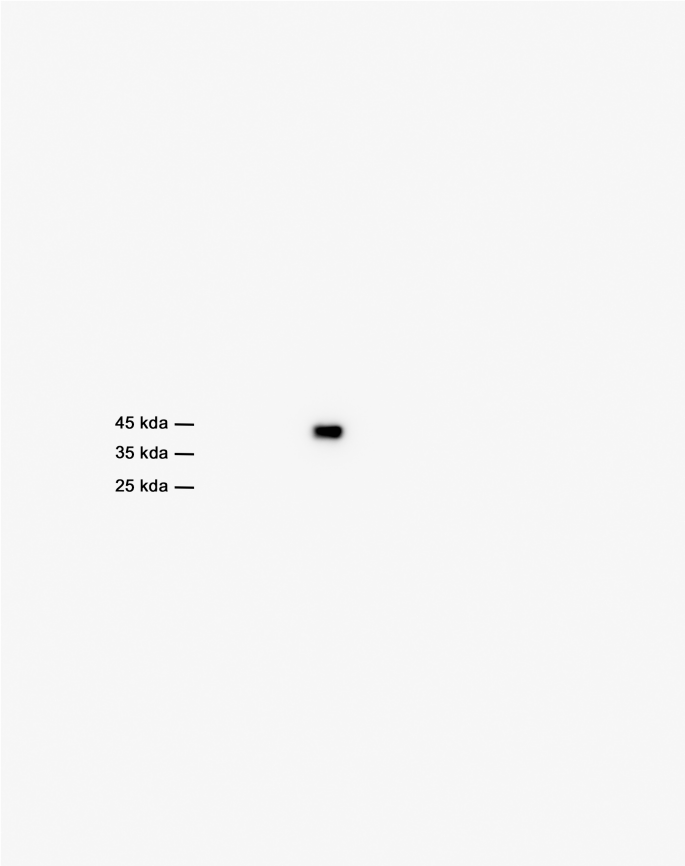
P4-135aa


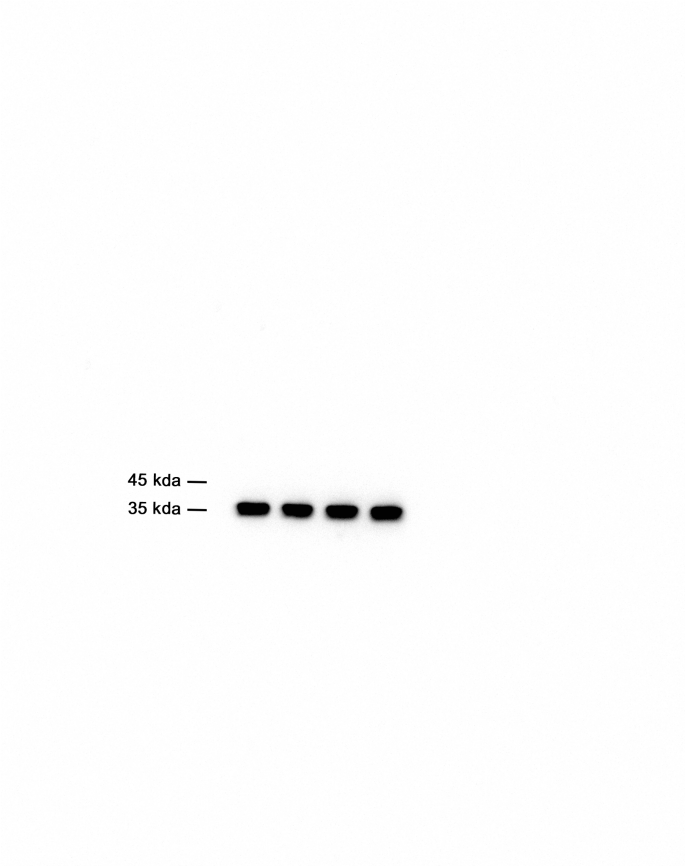
GAPDH

U373


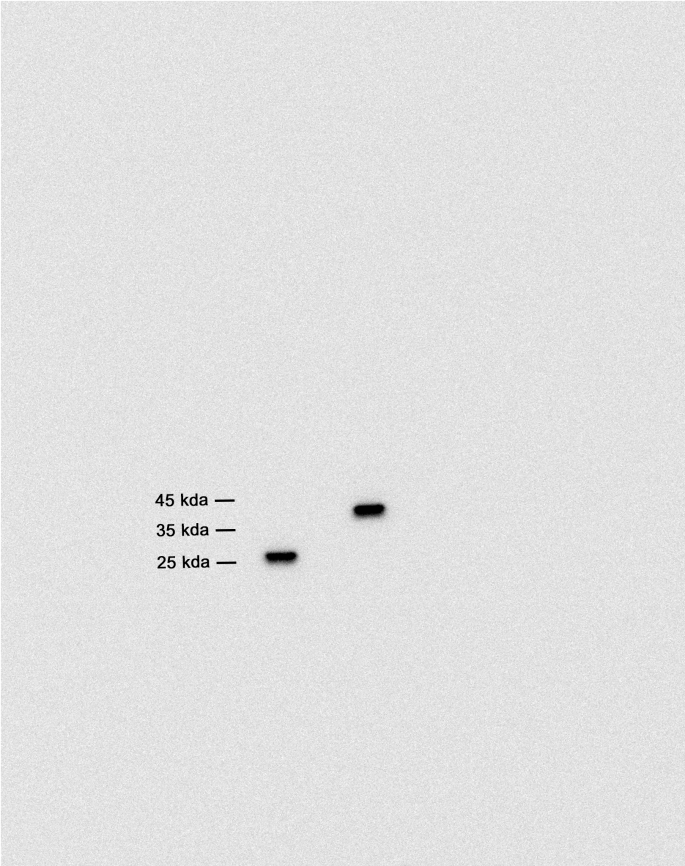
GFP


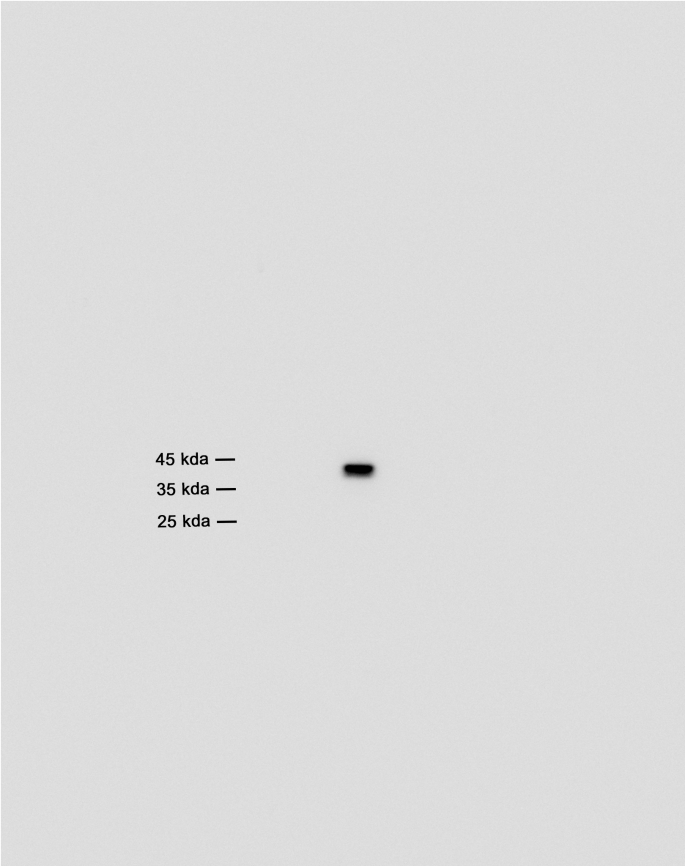
P4-135aa


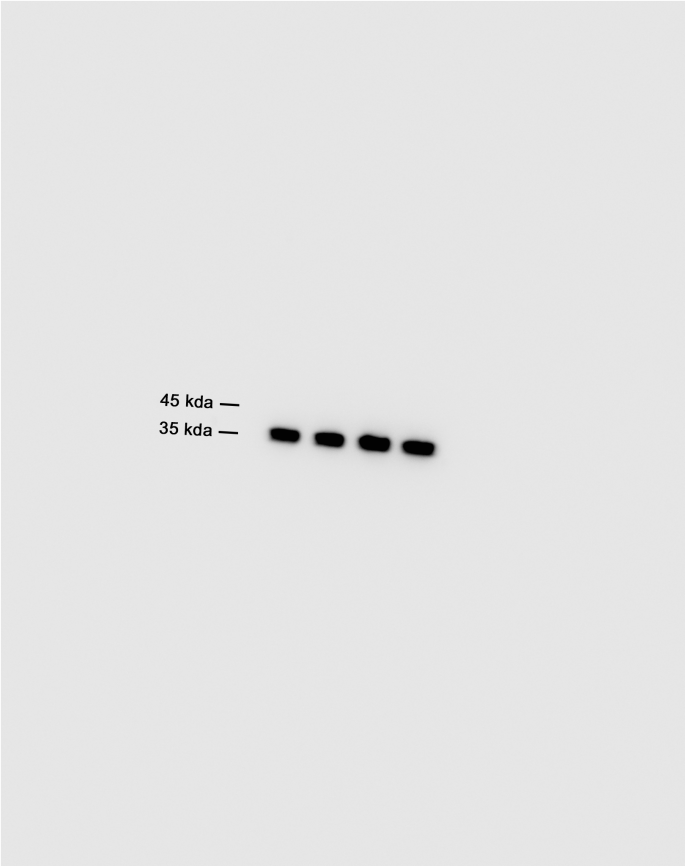
GAPDH

Figure S2.1 d

U251


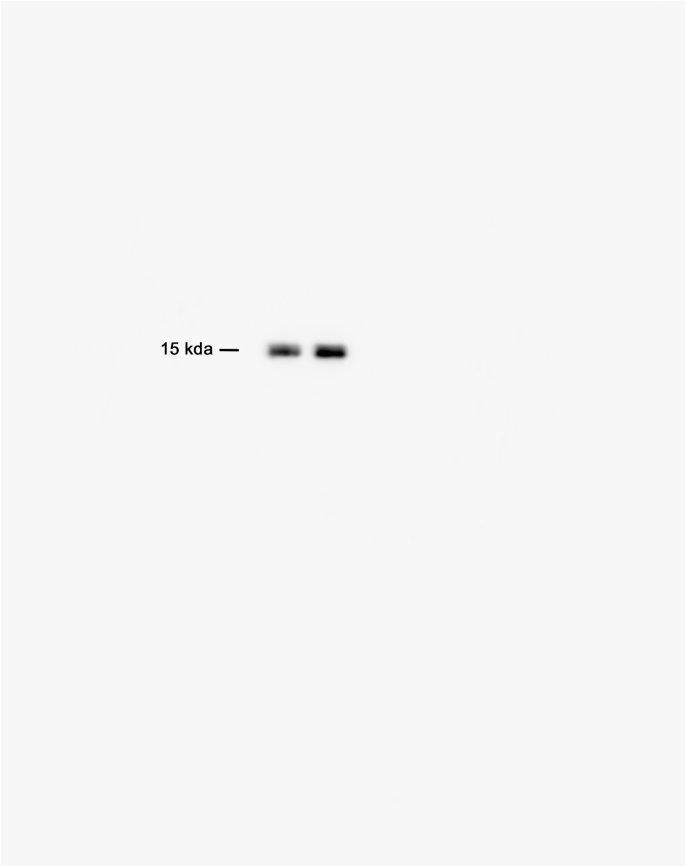
P4-135aa


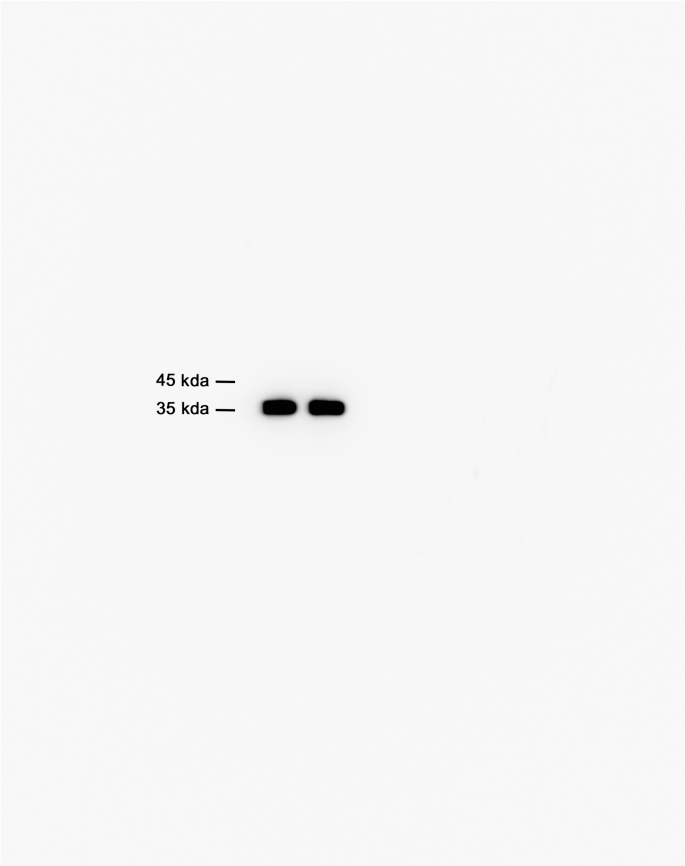
GAPDH

U373


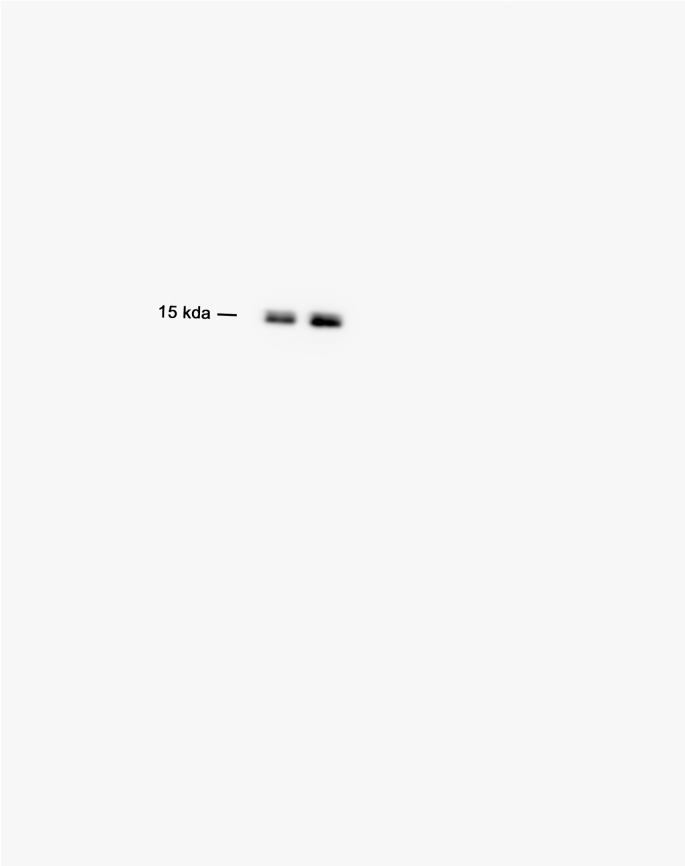
P4-135aa


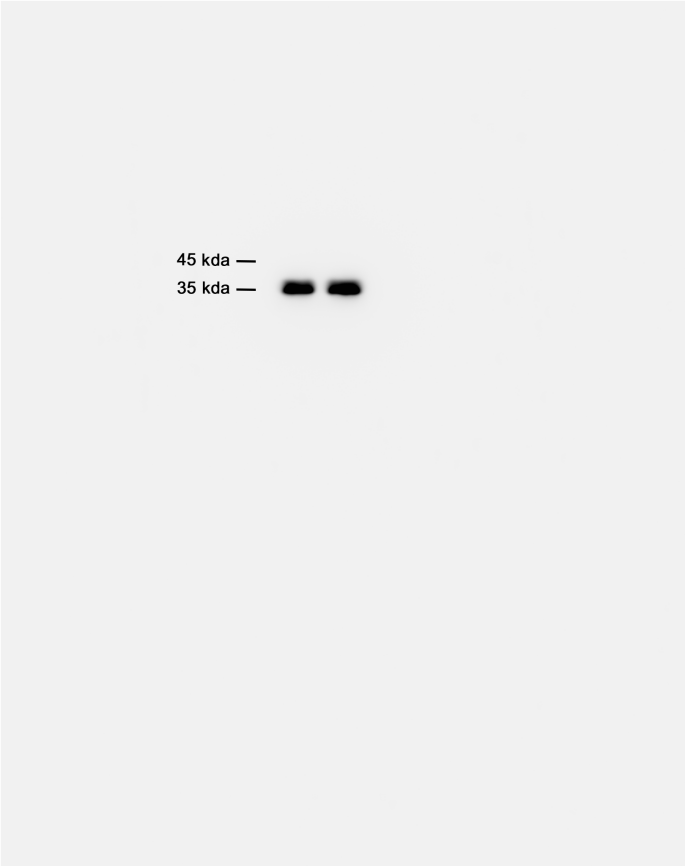
GAPDH

Figure S2.2 a

P4-135aa

GAPDH

Figure S2.2 b

P4-135aa

GAPDH

Figure S2.2 c

P4-135aa

GAPDH

Figure S2.2 d

VEGFR2

VE-cadherin

GAPDH

Figure S3.1 g

U251

VEGFR2

VE-cadherin

GAPDH

U373

VEGFR2

VE-cadherin

GAPDH

Figure S3.2 a

KLF15

GAPDH

Figure S3.2 c

VEGFR2

VE-cadherin

GAPDH

Figure S3.2 h

VEGFR2

VE-cadherin

GAPDH

Figure S4.1 a

U251

KLF15

GAPDH

U373

KLF15

GAPDH

Figure S4.1 b

U251

P4-135aa

KLF15

GAPDH

U373

P4-135aa

KLF15

GAPDH

Figure S4.1 c

Ubiquitin

KLF15-IP

P4-135aa-WCL

KLF15-WCL

GAPDH-WCL

Figure S4.1 e

U251

KLF15

GAPDH

Histone H3

U373

KLF15

GAPDH

Histone H3

Figure S4.2 a

VEGFR2

VE-cadherin

GAPDH

Figure S5.1 d

LDHA

GAPDH

LDHA

GAPDH

Figure S5.1 i

U251

VEGFR2

VE-cadherin

GAPDH

U373

VEGFR2

VE-cadherin

GAPDH

Figure S5.2 a

LDHA

GAPDH

Figure S5.2 b

LDHA

GAPDH

Figure S5.2 d

VEGFR2

VE-cadherin

GAPDH

Figure S5.2 i

VEGFR2

VE-cadherin

GAPDH

Figure S6c

VEGFR2

VE-cadherin

GAPDH

Figure S7c

U251

LDHA

VEGFR2

VE-cadherin

U373

LDHA

VEGFR2

VE-cadherin

Figure S7d

U251

VEGFR2

VE-cadherin

LDHA

GAPDH

U373

VEGFR2

VE-cadherin

LDHA

GAPDH
